# Electrochemically Induced Synthesis of Imidazoles from Vinyl Azides and Benzyl Amines

**DOI:** 10.3390/molecules27227721

**Published:** 2022-11-09

**Authors:** Vera A. Vil’, Sergei S. Grishin, Alexander O. Terent’ev

**Affiliations:** N. D. Zelinsky Institute of Organic Chemistry, Russian Academy of Sciences, 47 Leninsky Prospect, Moscow 119991, Russia

**Keywords:** electrochemistry, imidazoles, vinyl azides

## Abstract

An electrochemically induced synthesis of imidazoles from vinyl azides and benzyl amines was developed. A wide range of imidazoles were obtained, with yields of 30 to 64%. The discovered transformation is a multistep process whose main steps include the generation of electrophilic iodine species, 2*H*-azirine formation from the vinyl azide, followed by its reactions with benzyl amine and with imine generated from benzyl amine. The cyclization and aromatization of the obtained intermediate lead to the target imidazole. The synthesis proceeds under constant current conditions in an undivided cell. Despite possible cathodic reduction of various unsaturated intermediates with C=N bonds, the efficient electrochemically induced synthesis of imidazoles was carried out.

## 1. Introduction

**Scheme 1:**
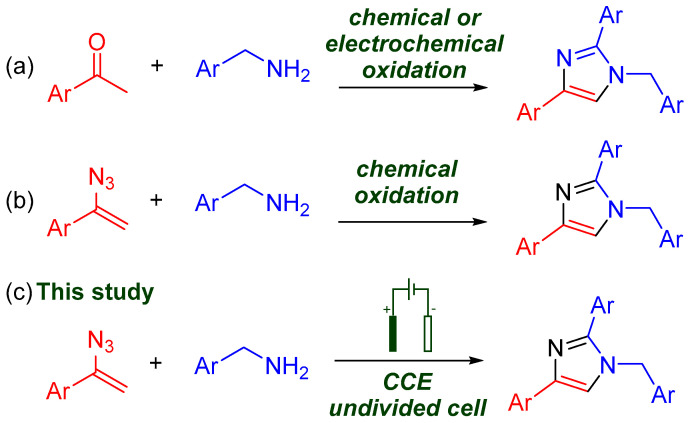
(**a**,**b**) Known methods for the synthesis of 1,2,4-trisubstituted imidazoles. (**c**) The presented herein method for imidazole synthesis.

The imidazole family of N-heterocycles is widely found in natural products and pharmaceutical compounds [1,2,3,4,5]. Additionally, imidazoles are used in organic chemistry and material design [6,7]. As a result, various methods have been developed to synthesize 1,2,4-trisubstituted-(1*H*)-imidazoles [8,9,10]. Substituted imidazoles were obtained from aryl ketones and benzylamines using N-heterocyclic carbene (NHC)/BF_3_·Et_2_O/*tert*-butyl hydroperoxide (TBHP) [11], CuI/BF_3_·Et_2_O/O_2_ [12], I_2_/HCl/O_2_ [13], and NaIO_4_/2,2,6,6-tetramethylpiperidin-1-yl)oxyl (TEMPO) [14] systems (Scheme 1a). The electrochemical cyclization of aryl ketones [15,16] with benzyl amines into 1,2,4-trisubstituted-(1*H*)-imidazoles was also reported (Scheme 1a). Approaches to 1,2,4-trisubstituted imidazoles via cyclization of enamides [17] and enamines [18] with benzyl amines were developed. However, there are some problems associated with these strategies, due to necessity of stoichiometric chemical reagents or heavy metal residues. Therefore, metal-free and stoichiometric-oxidant-free multicomponent strategies for the synthesis of imidazoles are highly desirable.

Vinyl azides have versatile reactivity: they can be nucleophiles, electrophiles, 1,3-dipoles, or radical acceptors [19,20]. Due to their ability to eliminate N_2_ during transformation, vinyl azides are convenient precursors in organic synthesis [21,22,23]. It is becoming increasingly popular to use vinyl azides as substrates for the preparation of N-heterocyclic compounds [24]. Oxidative cyclization of vinyl azides and benzyl amines into substituted imidazoles using I_2_/TBHP was reported (Scheme 1b) [25].

Nowadays, electro-organic synthesis is considered to be among the areas of organic chemistry with the most active development [26,27,28,29,30,31,32,33]. Currently, much attention is devoted to electrochemical synthesis of heterocyclic structures [34,35,36,37,38,39,40], which have always been essential scaffolds in organic chemistry. However, there are only a few examples of the electrochemically induced formation of the C−N bond for the synthesis of heterocyclic compounds such as imidazoles [15,41,42,43].

The electrolysis can be performed in an undivided or divided cell under controlled potential (CPE) or constant current conditions (CCE). Constant current (CCE) conditions benefit from the high current density, shorter process time, and technically convenient reaction setup. Using an undivided cell is more practical, but at the same time, undesirable processes connected to the counter-electrode action at reaction intermediates must be avoided.

As far as we know, the electrochemical method for the synthesis of substituted imidazoles using vinyl azides has not been disclosed yet. Inspired by the synthetic application of vinyl azides and our experience in the organic electrosynthesis [44], herein, we report an electrochemical approach to imidazoles from vinyl azides and benzyl amines (Scheme 1c).

## 2. Results and Discussion

We began testing our hypotheses using (1-azidovinyl)benzene **1a** and benzyl amine **2a** as the model substrates in N,N-dimethylformamide (DMF) with tetrabutylammonium iodide (TBAI) as the electrolyte under electrolysis at 30 mA (*j* = 10 mA/cm^2^) with 4.0 F/mol electricity passed, as shown in Table 1. To our delight, the expected 1-benzyl-2,4-diphenyl-1*H*-imidazole (**3a**) was isolated, with a yield of 24% from the crude mixture (entry 1, Table 1). We then investigated other reaction parameters. The detailed optimization of the imidazole electrosynthesis is presented in SI (Table S1).

Screening of electrolytes indicated that KI (37%, entry 2) was superior to TBAI and LiClO_4_, which gave lower yields (entries 1 and 3, 24% and 22%, respectively). The yield of **3a** has not risen with an increase in benzyl amine amount to 4.0 eq. (entry 4). The addition of *p*-TsOH·H_2_O improved the **3a** yield up to 48% (entry 5). The product (**3a**) was isolated, with a yield of 39% with the applied current density 20 mA/cm^2^ (entry 6). The yield increased as the amount of electricity passed achieved 6.0 F/mol (entry 7). When the electric current was absent, traces of the product formed (entry 8). Several acids were later screened. When H_2_SO_4_ or CH_3_SO_3_H were employed, the expected product (**3a**) was not detected (entries 9, 10); the replacement of *p*-TsOH·H_2_O with Amberlyst-15 dramatically lowered the yield (entry 11). To compare the influence of the cathode materials, the reaction was carried out with glassy carbon, stainless steel, and nickel cathodes (entries 12–14). Nickel cathode was almost as effective as platinum (48%, entries 7 and 14), the others were less. Product **3a** was obtained with a yield of 36% when a graphite plate was employed as anode (entry 15), and even lower yield was observed with platinum plate as anode (entry 16). Similar efficiency was obtained when dimethyl sulfoxide (DMSO) (34%, entry 17) was used as a solvent. However, the yield of product **3a** decreased to 18% when the reaction was performed in PhCl (entry 18). The reduction in the temperature to 70 °C led to the best **3a** yield (61%, entry 19). A further reduction in the temperature to 50 °C significantly drops the yield of the cyclization product, suggesting that the temperature also plays a crucial role in the electrochemical cyclization (entry 20). Under optimal conditions (yield **3a** 61%, entry 19), a complete conversion of **1a** was observed, with no evidence of byproducts that could be isolated.

**Scheme 2:**
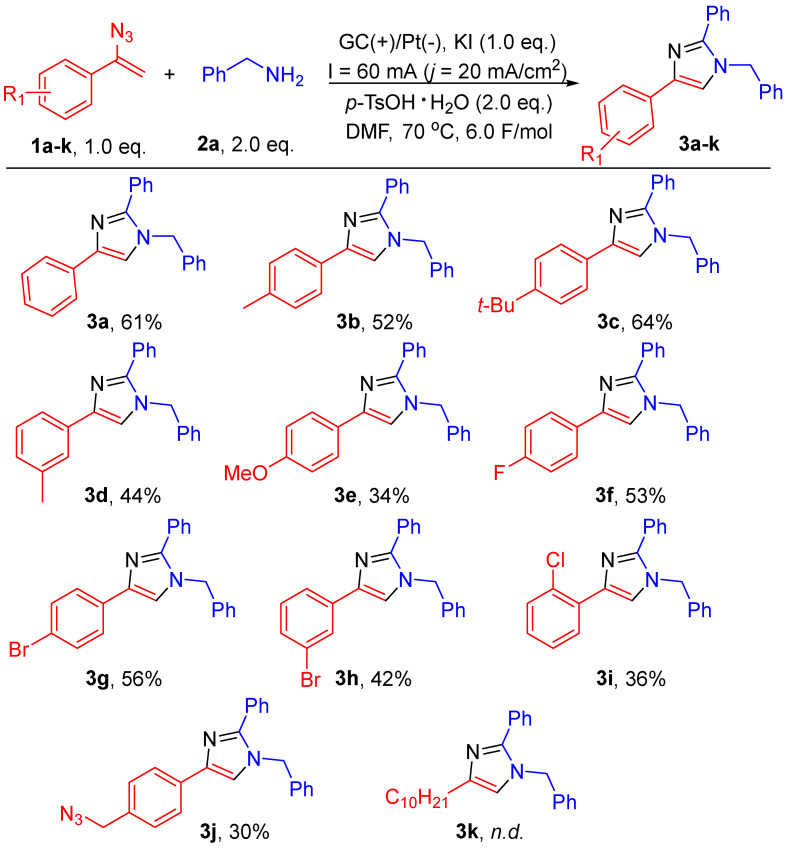
The scope of vinyl azides **1**.

With the best conditions in hand (Table 1, entry 19), we next turned our attention to the scope of various vinyl azides **1** as depicted in Scheme 2. Substituted (1-azidovinyl)benzenes **1** were subjected to transformation under the reaction conditions. All (1-azidovinyl)benzenes **1** containing electron-donating (e.g., CH_3_, *t*-Bu, and OCH_3_) as well as electron-withdrawing groups (e.g., F, Br, and Cl) worked well, affording the desired products **3a**–**3i** with yields of 34–64%. (1-Azidovinyl)benzene **1j** with the other azido-group gave the desired product **3j** with a yield of 30%. The aliphatic vinyl azide **1k** did not provide the cyclization product, and the possible reason is insufficient stabilization of the imine-enamine intermediates due to the lack of a conjugated bond system.

**Scheme 3:**
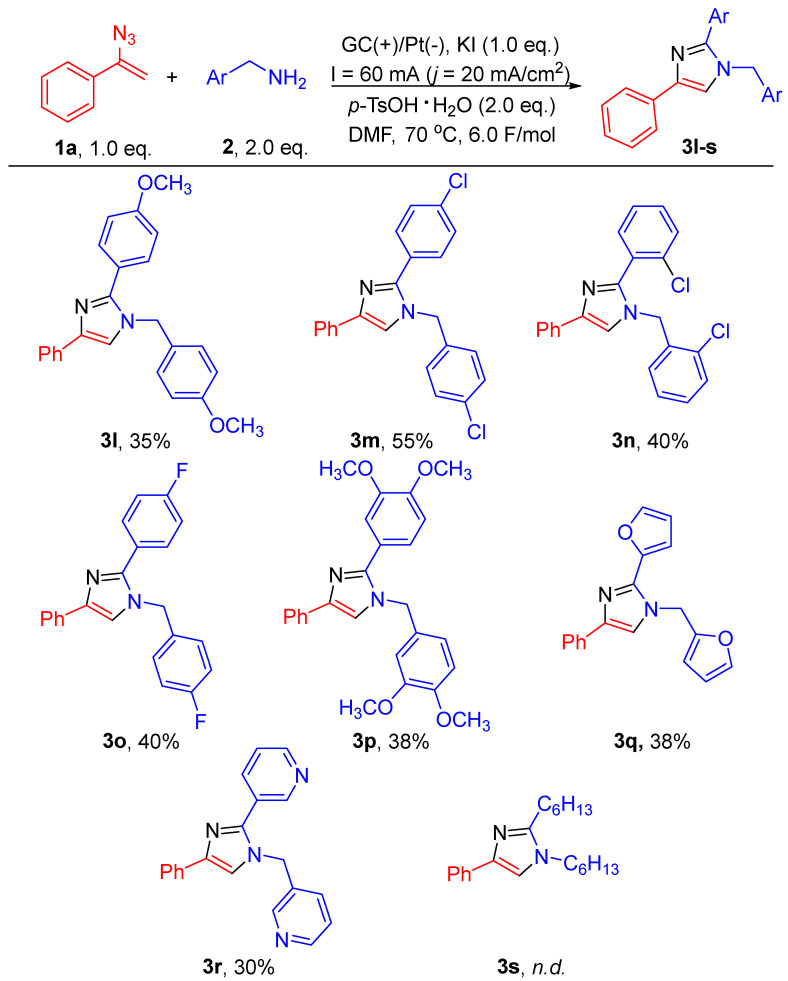
The scope of benzyl amines **2**.

Subsequently, representative amines **2** with electron-donating and electron-withdrawing groups were evaluated (Scheme 3). The various benzyl amines **2** were suitable for this transformation, giving the desired products **3l**–**p**, with yields of 35–55%. 2-(Aminomethyl)furan and 3-(aminomethyl)pyridine afforded the corresponding products **3q** and **3r** with yields of 38% and 30%, respectively. The application of 1-aminohexane did not lead to the cyclization product.

**Scheme 4:**
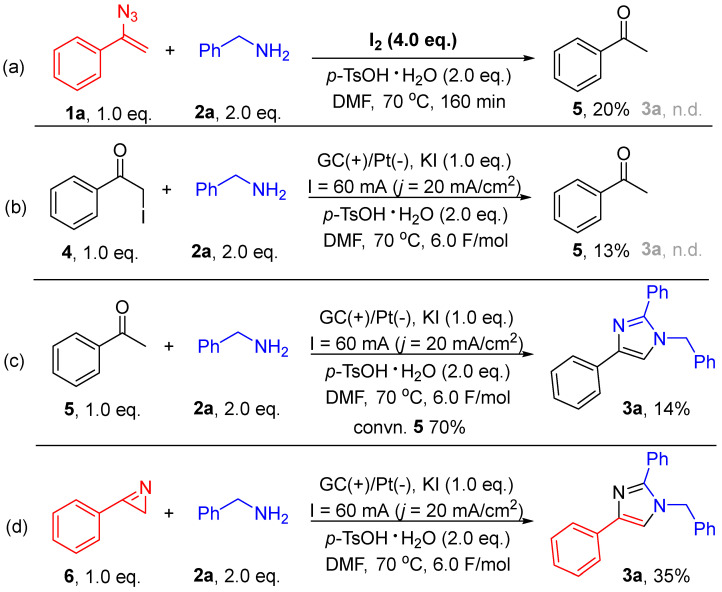
Control experiments. (**a**) The use of iodine as the oxidant. (**b**,**c**) The electrolysis of benzyl amine with ω-iodoacetophenone **4** and acetophenone **5**. (**d**) The electrolysis of benzyl amine with 3-phenyl-2*H*-azirine **6**.

In order to determine the reaction mechanism, we conducted a series of control experiments (Scheme 4). Firstly, **1a** and **2a** were placed with iodine (4.0 eq.) as the oxidant, so the target product **3a** was not observed, and acetophenone was isolated in a 20% yield (Scheme 4a). This result demonstrated the unique reactivity of the electrochemical system, which is far more complex than the iodine generation. Moreover, ω-iodoacetophenone **4** and acetophenone **5**, instead of **1a**, were investigated under electrochemical conditions (Scheme 4b,c). The reaction of ω-iodoacetophenone **4** with benzyl amine **2a** did not lead to the desired imidazole **3a**, unlike the reaction of acetophenone **5** with benzyl amine **2a**. The imidazole **3a** was synthesized from acetophenone, **5** with a low yield of 14% under optimal conditions (Scheme 4c). These results implied that the iodination of α-carbon in the vinyl substrate might not be the required reaction step. The substrate **2a** was employed to react with 3-phenyl-2*H*-azirine **6**; the product **3a** was obtained with a yield of 35%, thus 3-phenyl-2*H*-azirine **6** might be the intermediate in electrochemically induced synthesis of substituted imidazoles (Scheme 4d).

**Scheme 5:**
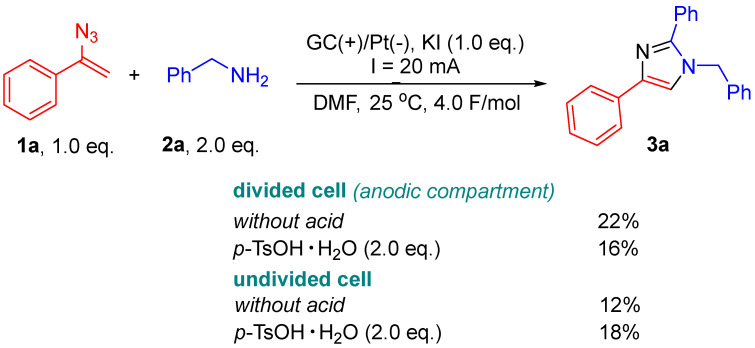
Comparing the divided and undivided electrochemical cells.

To understand the influence of the cathodic processes and *p*-TsOH on the yield of **3a**, we performed comparative electrochemical experiments in undivided and divided electrochemical cells (Scheme 5). There is no significant difference in **3a** yield between an undivided and divided electrochemical cells in the presence of *p*-TsOH. Without acid the reaction in the divided cell resulted in higher yields than in the undivided one. So, in the undivided cell, an acid is likely reduced on the cathode, preventing cathodic side processes.

**Figure 1:**
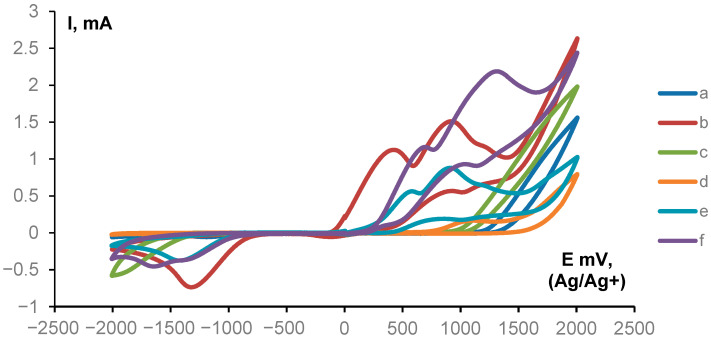
CV curves for the corresponding solutions on a working glassy-carbon electrode (d = 3 mm) under a scan rate of 0.1 V/s at 20 °C. (a) A 0.2 M solution of *p*-TsOH∙H_2_O in 0.1 M *n*-Bu_4_NBF_4_ solution in DMF; (b) a 0.1 M solution of KI in a 0.2 M solution of *p*-TsOH∙H_2_O in 0.1 M *n*-Bu_4_NBF_4_ solution in DMF; (c) mixture of vinyl azide **1a** and *p*-TsOH∙H_2_O in 0.1 M *n*-Bu_4_NBF_4_ solution in DMF; (d) mixture of amine **2a** and *p*-TsOH∙H_2_O in 0.1 M *n*-Bu_4_NBF_4_ solution in DMF; (e) mixture of amine **2a** and *p*-TsOH∙H_2_O in a 0.1 M solution of KI in 0.1 M *n*-Bu_4_NBF_4_ solution in DMF; (f) mixture of vinyl azide **1a**, amine **2a** and *p*-TsOH∙H_2_O in a 0.1 M solution of KI in 0.1 M *n*-Bu_4_NBF_4_ solution in DMF.

Cyclic voltammetry (CV) was used to study the redox potentials of the substrates (Figure 1). The mixture of DMF and *p*-TsOH∙H_2_O did not show considerable reactivity in anodic oxidation under the potential below 1.4 V (curve a). The KI demonstrated two reversible anodic waves at 0.4 and 0.9 V in the presence of the acid, which is in accordance with the triiodide (I_3_^−^) and iodine (I_2_) formation (curve b) [45]. The CV of vinyl azide **1a** and *p*-TsOH∙H_2_O exhibited a broad and reversible wave above 1.0 V (curve c). The mixture of benzyl amine **2a** and *p*-TsOH∙H_2_O turned out to be electrochemically inert under the potential below 1.5 V (curve d). The addition of KI to the mixture of benzyl amine **2a** and *p*-TsOH∙H_2_O led to decreased oxidation and reduction peaks of KI (curves b, d, e). The mixture vinyl azide **1a**, benzyl amine **2a**, KI, and *p*-TsOH∙H_2_O (curves e, f) demonstrated increased oxidation peaks, which may indicate the oxidation of the reaction products from vinyl azide **1a** with electrochemically generated intermediates from benzyl amine **2a**, KI, and *p*-TsOH∙H_2_O.

**Scheme 6:**
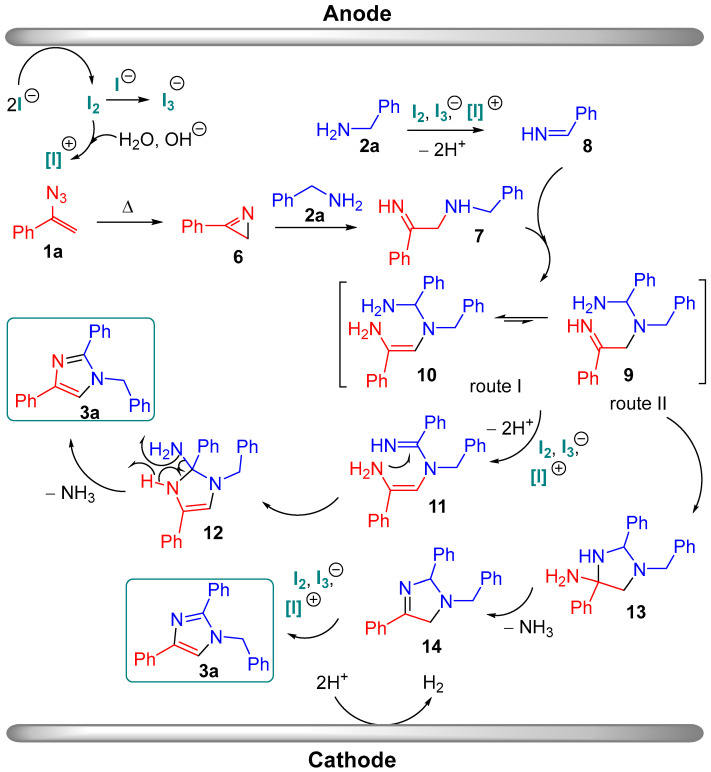
The proposed reaction mechanism.

Based on our experimental results and previous works [17,25,46], a plausible reaction mechanism for electrochemical transformation of vinyl azides and benzyl amines into imidazoles was proposed in Scheme 6. First, molecular iodine is generated via the anodic oxidation of I^−^ [15,16,22]. Generated I_2_ can further react with the I^−^ to result in I_3_^−^ formation or with traces of water from the solvent, as well as OH^−^ generated on the cathode, to give electrophilic iodine species [47,48]. Then, molecular iodine oxidizes **2a** to form imine **8**. The vinyl azide **1a** is converted to 2*H*-azirine **6** by thermal decomposition. A nucleophilic attack of starting benzyl amine **2a** to 2*H*-azirine **6** leads to intermediate **7** [25]. Subsequently, **7** reacts with imine **8** to provide intermediate **9**, which has imine-enamine tautomerism with intermediate **10** under optimized conditions. Due to this tautomerism, two routes are possible. The route I includes the oxidation of **10** into intermediate **11**, further cyclization into intermediate **12**, and ammonia elimination providing the target imidazole **3a**. According to route II, intermediate **9** cyclizes into imidazolidine **13**, which eliminates NH_3_ with the formation of intermediate **14**. The oxidation of **14** results in imidazole **3a**.

## 3. Materials and Methods

### 3.1. General Materials and Methods

^1^H and ^13^C NMR spectra were recorded on Bruker AVANCE II 300 spectrometer (300.13 and 75.48 MHz, respectively) in CDCl_3_. Chemical shifts were reported in parts per million (ppm), and the residual solvent peak was used as an internal reference: ^1^H (CDCl_3_ δ = 7.25 ppm), ^13^C (CDCl_3_ δ = 77.00 ppm). Multiplicity was indicated as follows: s (singlet), d (doublet), t (triplet), q (quartet), sept (septet), m (multiplet).

High-resolution mass spectra (HR-MS) were measured on a Bruker micrOTOF II instrument using electrospray ionization (ESI). The measurements were performed in a positive ion mode (interface capillary voltage—4500 V); mass range from *m*/*z* 50 to *m*/*z* 3000 Da; external calibration with Electrospray Calibrant Solution (Fluka). A syringe injection was used for all acetonitrile solutions (flow rate 3 µL/min). Nitrogen was applied as a dry gas; interface temperature was set at 180 °C.

FT-IR spectra were recorded on Bruker Alpha instrument.

The TLC analysis was carried out on standard silica gel chromatography plates (DC-Fertigfolien ALUGRAM^R^ Xtra SIL G/UV_254_). Column chromatography was performed using silica gel (0.040–0.060 mm, 60 A).

DMF, *p*-TsOH°H_2_O, TBAI, KI, NH_4_I, NH_4_Br, LiClO_4_, H_2_SO_4,_ CH_3_SO_3_H, Amberlyst-15, and chlorobenzene were purchased from commercial sources and were used as is. All solvents were distilled before use using standard procedures.

### 3.2. Synthesis of Starting Compounds

(1-Azidovinyl)benzene (**1a**), 1-(1-azidovinyl)-4-methylbenzene (**1b**), 1-(1-azidovinyl)-4-tertbutylbenzene (**1c**), 1-(1-azidovinyl)-3-methylbenzene (**1d**), 1-(1-azidovinyl)-4-methoxylbenzene (**1e**), 1-(1-azidovinyl)-4-fluorobenzene (**1f**), 1-(1-azidovinyl)-4-bromobenzene (**1g**), 1-(1-azidovinyl)-3-bromobenzene (**1h**), and 1-(1-azidovinyl)-2-chlorobenzene (**1i**) were synthesized according to the literature through the bromination of corresponding styrenes followed by the reaction of dibromides with NaN_3_ [49]. 1-(Azidomethyl)-4-(1-azidovinyl)benzene (**1j**) was synthesized according the same procedure from 1-(chloromethyl)-4-vinylbenzene as a result of simultaneous azidation of formed dibromide and nucleophilic substitution of chlorine atom [49]. 2-Azidododec-1-ene was synthesized according to the literature through the reaction between styrenes and I_2_/NaN_3_ system followed by dehydroiodination with *t*-BuOK [50].

Amines **2** were obtained from commercial suppliers and used without further purification.

### 3.3. Electrochemical Cell

For the electrosynthesis glassy carbon and platinum plates from Russian commercial suppliers were used as electrodes (glassy carbon: CУ-2000: TУ 1916-027-27208846-01; platinum grade: AISI 304). The reactions were performed in a common chemical tube. Undivided electrochemical cell equipped with glassy carbon plate anode and platinum plate cathode with reaction mixture during electrolysis under constant current conditions. The detailed electrochemical equipment was presented in our previous study [44].

Before all electrochemical reactions, the electrodes were placed into a 5 M solution of KOH and this mixture was electrolyzed for 10 min at *j* = 200 mA/cm^2^. After that, the polarity of electrodes was changed and the mixture was electrolyzed under these conditions again. After electrolysis, the electrodes were washed with running water and then with acetone. All these procedures help to clean the electrodes from the impurities from the previous electrolysis.

### 3.4. General Experimental Procedure for Scheme 2 and Scheme 3

An undivided cell was equipped with a glassy carbon anode (3 cm^2^) and a platinum plate cathode (3 cm^2^) and connected to a DC regulated power supply. The solution of **1a** (1.0 mmol, 1.0 eq.), **2a** (2.0 mmol, 2.0 eq.), *p*-TsOH°H_2_O (2.0 mmol, 380.0 mg, 2.0 eq.), and KI (1.0 mmol, 166.0 mg, 1.0 eq.) in 10 mL of DMF was electrolyzed using constant current conditions at 70 °C under magnetic stirring for 160 min with I = 60 mA (*j* = 20 mA/cm^2^). After that, the reaction mixture was diluted with H_2_O (30 ml) and washed with mixture of PE and ethyl acetate (1:1) (2 × 30 mL). The combined organic layer was washed with a 0.3 M solution of Na_2_S_2_O_3_ (2 × 10 mL), water (2 × 10 mL), dried over Na_2_SO_4_ and concentrated under reduced pressure using a rotary evaporator (15–20 mmHg), (bath temperature, ca. 30–40 °C). Product **3** was isolated by chromatography on SiO_2_.

#### 3.4.1. 1-Benzyl-2,4-diphenyl-1*H*-imidazole (**3a**)

Yellow solid. Yield 61% (189.7 mg, 0.61 mmol, PE/EtOAc = from 15:1 to 2:1 as eluent), mp = 123–124 °C (lit. [25] mp = 123–124 °C). R*_f_* = 0.36 (PE:EtOAc = 5:1). ^1^H NMR (300.13 MHz, CDCl_3_, δ): 7.88 (d, *J* = 7.4 Hz, 2H), 7.68–7.58 (m, 2H), 7.51–7.43 (m, 3H), 7.43–7.35 (m, 5H), 7.33–7.27 (m, 2H), 7.23–7.13 (m, 2H), 5.26 (s, 2H). ^13^C{^1^H} NMR (75.48 MHz, CDCl_3_, δ): 148.7, 141.6, 136.9, 134.2, 130.5, 129.1, 128.7, 128.6, 128.0, 126.9, 126.7, 125.0, 116.9, 50.5. HRMS (ESI-TOF) *m*/*z* [M + H]^+^. Calcd for [C_22_H_19_N_2_]^+^: 311.1543. Found: 311.1543. IR (KBr): 3469, 3034, 1651, 1474, 1446, 1398, 772, 736, 697 cm^−1^. The compound was previously described in [25].

#### 3.4.2. 1-Benzyl-2-phenyl-4-(p-tolyl)-1*H*-imidazole (**3b**)

Yellow solid. Yield 52% (168.5 mg, 0.52 mmol, PE/EtOAc = from 15:1 to 2:1 as eluent), mp = 140–142 °C (lit. [25] mp = 138–140 °C). R*_f_* = 0.37 (PE:EtOAc = 5:1). ^1^H NMR (300.13 MHz, CDCl_3_, δ): 7.75 (d, *J* = 8.0 Hz, 2H), 7.68–7.58 (m, 2H), 7.47–7.38 (m, 3H), 7.37–7.28 (m, 3H), 7.24–7.10 (m, 5H), 5.21 (s, 2H), 2.36 (s, 3H). ^13^C{^1^H} NMR (75.48 MHz, CDCl_3_, δ): 148.5, 141.6, 137.0, 136.6, 131.2, 130.5, 129.3, 129.2, 129.1, 128.7, 128.1, 126.8, 125.0, 116.5, 50.6, 21.3. HRMS (ESI-TOF) *m*/*z* [M + H]^+^. Calcd for [C_23_H_21_N_2_]^+^: 325.1699. Found: 325.1696. IR (KBr): 3542, 3498, 3468, 3438, 3066, 3029, 2957, 2924, 2855, 1729, 1644, 1646, 1273, 1178, 822, 763, 731, 698 cm^−1^. The compound was previously described in [25].

#### 3.4.3. 1-Benzyl-4-(4-(*tert*-butyl)phenyl)-2-phenyl-1*H*-imidazole (**3c**)

Yellow liquid. Yield 64% (234.6 mg, 0.64 mmol, PE/EtOAc = from 15:1 to 2:1 as eluent). R*_f_* = 0.25 (PE:EtOAc = 5:1). ^1^H NMR (300.13 MHz, CDCl_3_, δ): 7.80 (d, *J* = 8.3 Hz, 2H), 7.68–7.58 (m, 2H), 7.46–7.37 (m, 5H), 7.37–7.29 (m, 3H), 7.23 (s, 1H), 7.13 (d, *J* = 6.6 Hz, 2H), 5.22 (s, 2H), 1.36 (s, 9H). ^13^C{^1^H} NMR (75.48 MHz, CDCl_3_, δ): 149.9, 148.5, 141.7, 137.1, 131.3, 130.6, 129.2, 129.1, 129.0, 128.7, 128.0, 126.7, 125.5, 124.8, 116.6, 50.6, 34.6, 31.5. HRMS (ESI-TOF) *m*/*z* [M + H]^+^. Calcd for [C_26_H_27_N_2_]^+^: 367.2169. Found: 367.2165. IR (KBr): 3465, 3444, 3142, 3116, 3064, 3029, 2957, 2866, 1604, 1495, 1470, 1451, 1415, 1365, 1202, 836, 773, 730, 699, 522 cm^−1^. The compound was previously described in [25].

#### 3.4.4. 1-Benzyl-2-phenyl-4-(m-tolyl)-1*H*-imidazole (**3d**)

Yellow solid. Yield 44% (142.7 mg, 0.44 mmol, PE/EtOAc = from 15:1 to 2:1 as eluent), mp = 124–126 °C (lit. [15] mp = 125–126 °C). R*_f_* = 0.23 (PE:EtOAc = 5:1). ^1^H NMR (300.13 MHz, CDCl_3_, δ): 7.78 (s, 1H), 7.69–7.59 (m, 3H), 7.47–7.38 (m, 3H), 7.39–7.27 (m, 4H), 7.24 (s, 1H), 7.17–7.07 (m, 3H), 5.18 (s, 2H), 2.41 (s, 3H). ^13^C{^1^H} NMR (75.48 MHz, CDCl_3_, δ): 148.5, 141.5, 138.1, 136.9, 133.9, 130.4, 128.98, 128.95, 128.6, 128.4, 127.9, 127.6, 126.6, 125.6, 122.0, 116.9, 50.4, 21.5. HRMS (ESI-TOF) *m*/*z* [M + H]^+^. Calcd for [C_23_H_21_N_2_]^+^: 325.1699. Found: 325.1695. IR (KBr): 3471, 3134, 3030, 2952, 2918, 1604, 1449, 1402, 1359, 753, 696 cm^−1^. The compound was previously described in [15].

#### 3.4.5. 1-Benzyl-4-(4-methoxyphenyl)-2-phenyl-1*H*-imidazole (**3e**)

Yellow liquid. Yield 34% (115.6 mg, 0.34 mmol, PE/EtOAc = from 10:1 to 2:1 as eluent). R*_f_* = 0.13 (PE:EtOAc = 5:1). ^1^H NMR (300.13 MHz, CDCl_3_, δ): 7.78 (d, *J* = 8.6 Hz, 2H), 7.67–7.57 (m, 2H), 7.47–7.39 (m, 3 H), 7.38–7.30 (m, 3H), 7.19–7.10 (m, 3H), 6.92 (d, *J* = 8.6 Hz, 2H), 5.21 (s, 2H), 3.82 (s, 3H). ^13^C{^1^H} NMR (75.48 MHz, CDCl_3_, δ): 159.0, 148.4, 141.3, 136.9, 130.2, 129.21, 129.15, 128.8, 128.1, 126.8, 126.7, 126.4, 115.9, 114.1, 55.4, 50.7. HRMS (ESI-TOF) *m*/*z* [M + H]^+^. Calcd for [C_23_H_21_N_2_O]^+^: 341.1648. Found: 341.1649. IR (KBr): 3446, 3427, 3126, 3103, 3060, 3030, 2959, 2835, 1612, 1559, 1497, 1453, 1248, 1172, 1024, 833, 765, 698, 526 cm^−1^. The compound was previously described in [15].

#### 3.4.6. 1-Benzyl-4-(4-fluorophenyl)-2-phenyl-1*H*-imidazole (**3f**)

Yellow solid. Yield 53% (174.0 mg, 0.53 mmol, PE/EtOAc = from 10:1 to 2:1 as eluent), mp = 105–107 °C (lit. [25] mp = 106–107 °C). R*_f_* = 0.67 (PE:EtOAc = 2:1). ^1^H NMR (300.13 MHz, CDCl_3_, δ): 7.87–7.75 (m, 2H), 7.66–7.57 (m, 2H), 7.46–7.39 (m, 3H), 7.38–7.31 (m, 3H), 7.19 (s, 1H), 7.17–7.10 (m, 2H), 7.09–7.00 (m, 2H), 5.20 (s, 2H). ^13^C{^1^H} NMR (75.48 MHz, CDCl_3_, δ): 162.0 (d, *J* = 245.4 Hz), 148.7, 140.7, 136.8, 130.4 (d, *J* = 2.8 Hz), 129.12, 129.07, 129.0, 128.7, 128.1, 126.7, 126.60 (d, *J* = 7.9 Hz), 116.5, 115.4 (d, *J* = 21.5 Hz), 50.55. HRMS (ESI-TOF) *m*/*z* [M + H]^+^. Calcd for [C_22_H_18_FN_2_]^+^: 329.1449. Found: 329.1443. IR (KBr): 3458, 3059, 3032, 2357, 1644, 1495, 1348, 1218, 1156, 840, 761, 731, 695, 583, 519 cm^−1^. The compound was previously described in [25].

#### 3.4.7. 1-Benzyl-4-(4-bromophenyl)-2-phenyl-1*H*-imidazole (**3g**)

Yellow solid. Yield 56% (218.0 mg, 0.56 mmol, PE/EtOAc = from 10:1 to 2:1 as eluent), mp = 166–167 °C (lit. [25] mp = 164–166 °C). R*_f_* = 0.71 (PE:EtOAc = 2:1). ^1^H NMR (300.13 MHz, CDCl_3_, δ): 7.72 (d, *J* = 8.4 Hz, 2H), 7.66–7.57 (m, 2H), 7.48 (d, *J* = 8.4 Hz, 2H), 7.46–7.40 (m, 3H), 7.39–7.30 (m, 3H), 7.22 (s, 1H), 7.17–7.08 (m, 2H), 5.19 (s, 2H). ^13^C{^1^H} NMR (75.48 MHz, CDCl_3_, δ): 148.8, 140.4, 136.7, 133.1, 131.6, 130.2, 129.2, 129.1, 129.0, 128.7, 128.1, 126.8, 126.6, 120.5, 117.1, 50.6. HRMS (ESI-TOF) *m*/*z* [M + H]^+^. Calcd for [C_22_H_18_BrN_2_]^+^: 389.0648, 391.0628. Found: 389.0645, 391.0628. IR (KBr): 3472, 3129, 3059, 3025, 2977, 2952, 1599, 1550, 1478, 1413, 1188, 1070, 946, 830, 767, 701, 506 cm^−1^. The compound was previously described in [25].

#### 3.4.8. 1-Benzyl-4-(3-bromophenyl)-2-phenyl-1*H*-imidazole (**3h**)

Yellow solid. Yield 42% (163.5 mg, 0.42 mmol, PE/EtOAc = from 10:1 to 2:1 as eluent), mp = 104–106 °C. R*_f_* = 0.69 (PE:EtOAc = 2:1). ^1^H NMR (300.13 MHz, CDCl_3_, δ): 8.02 (s, 1H), 7.75 (d, *J* = 7.7 Hz, 1H), 7.66–7.56 (m, 2 H), 7.48–7.39 (m, 3H), 7.38–7.29 (m, 4H), 7.25–7.17 (m, 2H), 7.17–7.09 (m, 2H), 5.21 (s, 2H). ^13^C{^1^H} NMR (75.48 MHz, CDCl_3_, δ): 148.9, 140.2, 136.7, 136.3, 130.3, 130.2, 129.7, 129.3, 129.2, 129.1, 128.8, 128.2, 128.0, 126.8, 123.5, 122.9, 117.5, 50.7. HRMS (ESI-TOF) *m*/*z* [M + H]^+^. Calcd for [C_22_H_18_BrN_2_]^+^: 389.0648. Found: 389.0645. IR (KBr): 3477, 3129, 3062, 3031, 2951, 1600, 1566, 1468, 1450, 1205, 1072, 956, 872, 735, 697, 461 cm^−1^.

#### 3.4.9. 1-Benzyl-4-(2-chlorophenyl)-2-phenyl-1*H*-imidazole (**3i**)

Yellow liquid. Yield 36% (124.2 mg, 0.36 mmol, PE/EtOAc = from 15:1 from 2:1 as eluent). R*_f_* = 0.31 (PE:EtOAc = 5:1). ^1^H NMR (300.13 MHz, CDCl_3_, δ): 8.35 (dd, *J* = 7.9, 1.6 Hz, 1H), 7.77 (s, 1H), 7.67–7.58 (m, 2H), 7.46–7.39 (m, 4H), 7.38–7.30 (m, 4H), 7.22–7.10 (m, 3H), 5.26 (s, 2H). ^13^C{^1^H} NMR (75.48 MHz, CDCl_3_, δ): 147.8, 137.6, 136.9, 132.5, 130.9, 130.4, 130.2, 129.8, 129.12, 129.07, 128.7, 128.0, 127.5, 126.9, 126.6, 121.7, 50.6. HRMS (ESI-TOF) *m*/*z* [M + H]^+^. Calcd for [C_22_H_18_ClN_2_]^+^: 345.1153. Found: 345.1149. IR (KBr): 3449, 3147, 3057, 3031, 1473, 1451, 1426, 1351, 1182, 1047, 764, 736, 700, 560 cm^−1^. The compound was previously described in [25].

#### 3.4.10. 4-(4-(Azidomethyl)phenyl)-1-benzyl-2-phenyl-1*H*-imidazole (**3j**)

Yellow liquid. Yield 30% (109.6 mg, 0.30 mmol, PE/EtOAc = from 15:1 to 2:1 as eluent). R*_f_* = 0.55 (PE:EtOAc = 2:1). ^1^H NMR (300.13 MHz, CDCl_3_, δ): 7.86 (d, *J* = 8.2 Hz, 2H), 7.66–7.56 (m, 2H), 7.48–7.39 (m, 3H), 7.38–7.28 (m, 5H), 7.26 (s, 1H), 7.18–7.08 (m, 2H), 5.20 (s, 2H), 4.32 (s, 2H). ^13^C{^1^H} NMR (75.48 MHz, CDCl_3_, δ): 148.8, 141.0, 136.8, 134.2, 133.7, 130.4, 129.14, 129.07, 128.7, 128.6, 128.1, 126.8, 125.4, 117.2, 54.8, 50.6. HRMS (ESI-TOF) *m*/*z* [M + H]^+^. Calcd for [C_23_H_20_N_5_]^+^: 366.1713. Found: 366.1712. IR (KBr): 3108, 3063, 3031, 2929, 2875, 2098, 1613, 1498, 1471, 1452, 1422, 1355, 1249, 1181, 1075, 1021, 948, 848, 771, 732, 699 cm^−1^.

#### 3.4.11. 1-(4-Methoxybenzyl)-2-(4-methoxyphenyl)-4-phenyl-1*H*-imidazole (**3l**)

Yellow liquid. Yield 35% (129.7 mg, 0.35 mmol, PE/EtOAc = 5:1 as eluent). R*_f_* = 0.38 (PE:EtOAc = 2:1). ^1^H NMR (300.13 MHz, CDCl_3_, δ): 7.80 (d, *J* = 7.3 Hz, 2H), 7.57–7.47 (m, 2H), 7.33 (t, *J* = 7.6 Hz, 2H), 7.24–7.14 (m, 2H), 7.04 (d, *J* = 8.6 Hz, 2H), 6.96–6.89 (m, 2H), 6.88–6.78 (m, 2H), 5.09 (s, 2H), 3.81 (s, 3H), 3.77 (s, 1H). ^13^C{^1^H} NMR (75.48 MHz, CDCl_3_, δ): 160.3, 159.4, 148.5, 141.3, 134.3, 130.5, 129.0, 128.6, 128.2, 126.8, 125.0, 123.2, 116.5, 114.5, 114.1, 55.44, 55.42, 50.1. HRMS (ESI-TOF) *m*/*z* [M + H]^+^. Calcd for [C_24_H_23_N_2_O_2_]^+^: 371.1754. Found: 371.1751. IR (KBr): 3130, 3060, 3033, 3002, 2957, 2935, 2834, 1611, 1514, 1485, 1457, 1295, 1253, 1177, 1029, 838, 735, 697, 611, 518 cm^−1^. The compound was previously described in [25].

#### 3.4.12. 1-(4-Chlorobenzyl)-2-(4-chlorophenyl)-4-phenyl-1*H*-imidazole (**3m**)

Yellow liquid. Yield 55% (208.6 mg, 0.55 mmol, PE/EtOAc = from 15:1 to 2:1 as eluent). R*_f_* = 0.29 (PE:EtOAc = 5:1). ^1^H NMR (300.13 MHz, CDCl_3_, δ): 7.80 (d, *J* = 7.5 Hz, 2H), 7.54–7.44 (m, 2H), 7.42–7.35 (m, 4H), 7.34–7.27 (m, 2H), 7.26–7.18 (m, 2H), 7.01 (d, *J* = 8.3 Hz, 2H), 5.12 (s, 2H). ^13^C{^1^H} NMR (75.48 MHz, CDCl_3_, δ): 147.4, 142.0, 135.3, 135.1, 134.1, 133.8, 130.2, 129.4, 129.0, 128.8, 128.7, 128.0, 127.2, 125.0, 117.1, 50.0. HRMS (ESI-TOF) *m*/*z* [M + H]^+^. Calcd for [C_22_H_17_Cl_2_N_2_]^+^: 379.0763. Found: 379.0760. IR (KBr): 3130, 3062, 3032, 2931, 1896, 1606, 1489, 1450, 1411, 1180, 1092, 1014, 947, 910, 837, 732. 696, 488 cm^−1^. The compound was previously described in [25].

#### 3.4.13. 1-(2-Chlorobenzyl)-2-(2-chlorophenyl)-4-phenyl-1*H*-imidazole (**3n**)

Yellow solid. Yield 40% (151.7 mg, 0.40 mmol, PE/EtOAc = from 15:1 to 2:1 as eluent), mp = 134–136 °C (lit. [15] mp = 135–136 °C). R*_f_* = 0.22 (PE:EtOAc = 5:1). ^1^H NMR (300.13 MHz, CDCl_3_, δ): 7.81 (d, *J* = 7.4 Hz, 2H), 7.51–7.42 (m, 2H), 7.40–7.27 (m, 5H), 7.25–7.11 (m, 4H), 6.97–6.87 (m, 1H), 5.08 (s, 2H). ^13^C{^1^H} NMR (75.48 MHz, CDCl_3_, δ): 145.9, 141.5, 134.7, 133.9, 133.8, 133.1, 132.8, 131.1, 129.9, 129.7, 129.5, 129.3, 128.6, 127.3, 127.0, 126.9, 124.9, 115.9, 48.2. HRMS (ESI-TOF) *m*/*z* [M + H]^+^. Calcd for [C_22_H_17_Cl_2_N_2_]^+^: 379.0763. Found: 379.0761. IR (KBr): 3138, 3057, 2937, 2854, 1604, 1446, 1405, 1382, 1336, 1192, 1028, 948, 915, 753, 697, 506 cm^−1^. The compound was previously described in [15].

#### 3.4.14. 1-(4-Fluorobenzyl)-2-(4-fluorophenyl)-4-phenyl-1*H*-imidazole (**3o**)

Yellow liquid. Yield 40% (138.6 mg, 0.40 mmol, PE/EtOAc = from 10:1 to 2:1 as eluent). R*_f_* = 0.62 (PE:EtOAc = 2:1). ^1^H NMR (300.13 MHz, CDCl_3_, δ): 7.87–7.77 (m, 2H), 7.60–7.49 (m, 2H), 7.36 (t, *J* = 7.6 Hz, 2H), 7.28–7.22 (m, 1H), 7.21 (s, 1H), 7.16–7.09 (m, 1H), 7.09–6.97 (m, 5H), 5.12 (s, 2H). ^13^C{^1^H} NMR (75.48 MHz, CDCl_3_, δ): 164.5 (d, *J* = 247.0 Hz), 161.24 (d, *J* = 245.2Hz), 147.6, 141.7, 133.9, 132.4 (d, *J* = 3.3 Hz), 131.0 (d, *J* = 8.4 Hz), 128.7, 128.5 (d, *J* = 8.2 Hz), 127.1, 126.6 (d, *J* = 3.7 Hz), 125.0, 116.8, 116.1 (d, *J* = 19.4 Hz), 115.8 (d, *J* = 19.7 Hz), 49.9. ^19^F NMR (282 MHz, CDCl_3_) δ -112.31, -114.50. HRMS (ESI-TOF) *m*/*z* [M + H]^+^: Calcd for [C_22_H_17_F_2_N_2_]^+^: 347.1354. Found: 347.1354. IR (KBr): 3129, 3065, 3038, 2932, 1607, 1511, 1485, 1451, 1419, 1226, 1159, 1097, 1015, 947, 910, 843, 733, 697, 607, 505 cm^−1^. The compound was previously described in [15].

#### 3.4.15. 1-(3,4-Dimethoxybenzyl)-2-(3,4-dimethoxyphenyl)-4-phenyl-1*H*-imidazole (**3p**)

Yellow solid. Yield 38% (163.6 mg, 0.38 mmol, PE/EtOAc = 3:1 as eluent), mp = 181–183 °C (lit. [25] mp = 182–184 °C). R*_f_* = 0.15 (PE:EtOAc = 2:1). ^1^H NMR (300.13 MHz, CDCl_3_, δ): 7.84 (d, *J* = 7.4 Hz, 2H), 7.36 (t, *J* = 7.4 Hz, 2 H), 7.28–7.17 (m, 3H), 7.13 (d, *J* = 8.6 Hz, 1H), 6.94–6.79 (m, 2H), 6.74–6.66 (m, 1H), 6.63 (s, 1H), 5.16 (s, 2H), 3.90 (s, 3H), 3.87 (s, 3H), 3.82 (s, 3H), 3.80 (s, 3H). ^13^C{^1^H} NMR (75.48 MHz, CDCl_3_, δ): 149.9, 149.5, 149.1, 148.9, 148.4, 141.1, 133.9, 129.4, 128.6, 126.9, 125.0, 123.0, 121.6, 119.1, 116.7, 112.5, 111.6, 111.1, 109.9, 56.0, 55.9, 50.4. HRMS (ESI-TOF) *m*/*z* [M + H]^+^. Calcd for [C_26_H_27_N_2_O_4_]^+^: 431.1965. Found: 431.1969. IR (KBr): 3453, 3130, 3099, 3012, 2959,2936,2835, 1606, 1515, 1442, 1320, 1261, 1244, 1141, 1025, 812, 765, 723, 696 cm^−1^. The compound was previously described in [25].

#### 3.4.16. 2-(Furan-2-yl)-1-(furan-2-ylmethyl)-4-phenyl-1*H*-imidazole (**3q**)

Yellow liquid. Yield 38% (110.3 mg, 0.38 mmol, PE/EtOAc = from 10:1 to 2:1 as eluent). R*_f_* = 0.55 (PE:EtOAc = 2:1). ^1^H NMR (300.13 MHz, CDCl_3_, δ): 7.81 (d, *J* = 7.4 Hz, 2H), 7.58–7.51 (m, 1H), 7.40–7.30 (m, 3H), 7.25–7.20 (m, 2H), 6.96 (d, *J* = 3.4 Hz, 1H), 6.58–6.49 (m, 1H), 6.36–6.31 (m, 1H), 6.31–6.26 (m, 1H), 5.36 (s, 2H). ^13^C{^1^H} NMR (75.48 MHz, CDCl_3_, δ): 149.3, 145.4, 143.1, 142.9, 141.7, 139.1, 133.7, 128.6, 127.0, 125.1, 116.7, 111.7, 110.7, 110.4, 109.1, 44.0. HRMS (ESI-TOF) *m*/*z* [M + H]^+^. Calcd for [C_18_H_15_N_2_O_2_]^+^: 291.1128. Found: 291.1125. IR (KBr): 3124, 3061, 3032, 2928, 2853, 1678, 1606, 1482, 1446, 1343, 1222, 1185, 1149, 1073, 1011, 948, 909, 885, 816, 736, 696, 596, 504 cm^−1^. The compound was previously described in [15].

#### 3.4.17. 3-(4-Phenyl-1-(Pyridin-3-Ylmethyl)-1*H*-Imidazol-2-yl)Pyridine (**3r**)

Yellow liquid. Yield 30% (93.7 mg, 0.30 mmol, PE/EtOAc = 1:1 as eluent). R*_f_* = 0.10 (PE:EtOAc = 1:1). ^1^H NMR (300.13 MHz, CDCl_3_, δ): 8.80 (s, 1H), 8.61 (d, *J* = 4.7, 1H), 8.52 (d, *J* = 4.7, 1H), 8.40 (s, 1H), 7.88 (d, *J* = 7.8 Hz, 1H), 7.78 (d, *J* = 7.9 Hz, 2H), 7.40–7.29 (m, 4H), 7.29–7.19 (m, 3H), 5.20 (s, 2H). ^13^C{^1^H} NMR (75.48 MHz, CDCl_3_, δ): 150.1, 149.7, 149.3, 148.2, 145.4, 142.6, 136.4, 134.3, 133.4, 131.9, 128.7, 127.3, 126.5, 125.0, 124.0, 123.6, 117.3, 48.4. HRMS (ESI-TOF) *m*/*z* [M + H]^+^. Calcd for [C_20_H_17_N_4_]^+^: 313.1448. Found: 313.1440. IR (KBr): 3386, 3127, 3059, 3035, 2934, 2219, 1606, 1575, 1481, 1450, 1426, 1193, 1090, 1027, 912, 816, 731, 644, 507 cm^−1^.

## 4. Conclusions

In summary, we have disclosed the electrochemical synthesis of imidazoles from vinyl azides and benzyl amines in moderate to high yields. Application of an electric current makes it possible to conduct the reaction without application of unrecoverable chemical oxidants. The process was carried out under constant current conditions in an experimentally simple undivided electrochemical cell equipped with a platinum cathode and a glassy carbon anode. Potassium iodide served as both a supporting electrolyte and a redox catalyst. With the use of cyclic voltammetry and control experiments, a possible reaction pathway was proposed. Presumably, during the reaction, 2*H*-azirine is generated from the vinyl azide followed by its reaction with benzyl amine and the corresponding imine. The cyclization and aromatization of the obtained intermediate lead to the target imidazole.

## Figures and Tables

**Table 1:**
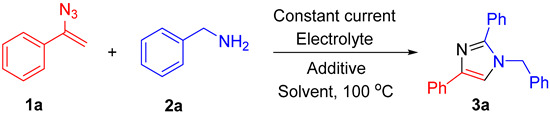
Optimization of imidazole electrosynthesis ^1^.

**Entry**	Cathode/Anode	Electrolyte (eq)	Additive (eq.)	Solvent	Current Density, mA/cm^2^	Electricity Passed per 1a, F/mol	Yield 3a, %
1	Pt/GC	TBAI (1.0)	-	DMF	10.0	4.0	24
2	Pt/GC	KI (1.0)	-	DMF	10.0	4.0	37
3	Pt/GC	LiClO_4_ (1.0)	-	DMF	10.0	4.0	22
4 ^2^	Pt/GC	KI (1.0)	-	DMF	10.0	4.0	33
5	Pt/GC	KI (1.0)	*p*-TsOH·H_2_O (2.0)	DMF	10.0	4.0	48
6	Pt/GC	KI (1.0)	*p*-TsOH·H_2_O (2.0)	DMF	20.0	4.0	39
7	Pt/GC	KI (1.0)	*p*-TsOH·H_2_O (2.0)	DMF	20.0	6.0	55
8	Pt/GC	KI (1.0)	*p*-TsOH·H_2_O (2.0)	DMF	-	-	7
9	Pt/GC	KI (1.0)	H_2_SO_4_ (2.0)	DMF	20.0	6.0	-
10	Pt/GC	KI (1.0)	CH_3_SO_3_H (2.0)	DMF	20.0	6.0	-
11	Pt/GC	KI (1.0)	Amberlyst-15 (2.0)	DMF	20.0	6.0	46
12	GC/GC	KI (1.0)	*p*-TsOH·H_2_O (2.0)	DMF	20.0	6.0	15
13	SS/GC	KI (1.0)	*p*-TsOH·H_2_O (2.0)	DMF	20.0	6.0	34
14	Ni/GC	KI (1.0)	*p*-TsOH·H_2_O (2.0)	DMF	20.0	6.0	48
15	Pt/C	KI (1.0)	*p*-TsOH·H_2_O (2.0)	DMF	20.0	6.0	36
16	Pt/Pt	KI (1.0)	*p*-TsOH·H_2_O (2.0)	DMF	20.0	6.0	22
17	Pt/GC	KI (1.0)	*p*-TsOH·H_2_O (2.0)	DMSO	20.0	6.0	34
18	Pt/GC	KI (1.0)	*p*-TsOH·H_2_O (2.0) n-Bu_4_NClO_4_ (1.0)	PhCl	20.0	6.0	18
19 ^3^	Pt/GC	KI (1.0)	*p*-TsOH·H_2_O (2.0)	DMF	20.0	6.0	61
20 ^4^	Pt/GC	KI (1.0)	*p*-TsOH·H_2_O (2.0)	DMF	20.0	6.0	31

^1^ General reaction conditions: undivided cell, glassy carbon plate anode/platinum plate cathode (3 cm^2^), constant current, **1a** (1.0 mmol, 145.2 mg), **2a** (2.0 mmol, 214.4 mg), solvent (10.0 mL), 100 °C, and air atmosphere. ^2^ **3a** (4.0 mmol, 428.8 mg). ^3^ 70 °C. ^4^ 50 °C.

## Data Availability

The data presented in this study are available in insert article or Supplementary Materials here.

## Supplementary Materials

The following supporting information can be downloaded at: https://www.mdpi.com/article/10.3390/molecules27227721/s1, Figure S1: CV curves; Table S1: Detailed optimization of imidazole electrosynthesis, characterization data of synthesized compounds, NMR spectra, HRMS, and IR spectra.

## Sample Availability

Samples of the compounds **3** are available from the authors.
