# Supplementary material for: Electrochemically Induced Synthesis of Imidazoles from Vinyl Azides and Benzyl Amines
Source: Molecules. 2022 Nov 9;27(22):7721. doi: 10.3390/molecules27227721 (PMC9692461; doi:10.3390/molecules27227721)

SUPPORTING INFORMATION

Electrochemically induced synthesis of imidazoles from vinyl azides and benzyl amines

*Vera A. Vil', Sergei S. Grishin, Alexander O. Terent'ev**

N. D. Zelinsky Institute of Organic Chemistry, Russian Academy of Sciences, 47 Leninsky Prospect, Moscow, 119991, Russian Federation. E-mail: alterex@yandex.ru, terentev@ioc.ac.ru

Table of content

General materials and methods S2
Synthesis of starting compounds S2
Electrochemical cell S2
Table S1. Detailed optimization of imidazole electrosynthesis S3
Experimental Procedures for Table S1........ S5
General Experimental Procedure for Schemes 2, 3. .... S9
CV study S16
Experimental Procedures for Scheme 4 .... S17
Experimental Procedures for Scheme 5. .... S17
References S18
NMR spectra of synthesized compounds S19
HRMS spectra of synthesized compounds........ S54
IR spectra of synthesized compounds........ S71

General materials and methods

^{1}H and ^{13}C NMR spectra were recorded on Bruker AVANCE II 300 spectrometer (300.13 and 75.48 MHz, respectively) in $CDCl_3$. Chemical shifts were reported in parts per million (ppm), and the residual solvent peak was used as an internal reference: ^{1}H ($CDCl_3$ δ=7.25 ppm), ^{13}C ($CDCl_3$ δ=77.00 ppm). Multiplicity was indicated as follows: s (singlet), d (doublet), t (triplet), q (quartet), sept (septet), m (multiplet).

High resolution mass spectra (HR-MS) were measured on a Bruker micrOTOF II instrument using electrospray ionization (ESI). The measurements were performed in a positive ion mode (interface capillary voltage - 4500 V); mass range from m/z 50 to m/z 3000 Da; external calibration with Electrospray Calibrant Solution (Fluka). A syringe injection was used for all acetonitrile solutions (flow rate 3 μL/min). Nitrogen was applied as a dry gas; interface temperature was set at 180 °C.

FT-IR spectra were recorded on Bruker Alpha instrument.

The TLC analysis was carried out on standard silica gel chromatography plates (DC-Fertigfolien ALUGRAM^{R} Xtra SIL G/UV_{254}). Column chromatography was performed using silica gel (0.040-0.060 mm, 60 A).

DMF, *p*-TsOH·H_2O, TBAI, KI, NH_4I, NH_4Br, $LiClO_4$, AcOH, HCOOH, H_2SO_4, CH_3SO_3H, Amberlyst-15, Lewatit MonoPlus SP-112-H, *p*-chlorobenzene were purchased from commercial sources and were used as is. All solvents were distilled before use using standard procedures.

Synthesis of starting compounds

(1-Azidovinyl)benzene (**1a**), 1-(1-azidovinyl)-4-methylbenzene (**1b**), 1-(1-azidovinyl)-4-tertbutylbenzene (**1c**), 1-(1-azidovinyl)-3-methylbenzene (**1d**), 1-(1-azidovinyl)-4-methoxylbenzene (**1e**), 1-(1-azidovinyl)-4-fluorobenzene (**1f**), 1-(1-azidovinyl)-4-bromobenzene (**1g**), 1-(1-azidovinyl)-3-bromobenzene (**1h**), 1-(1-azidovinyl)-2-chlorobenzene (**1i**) were synthesized according to the literature through the bromination of corresponding styrenes followed by the reaction of dibromides with NaN_3.[1] 1-(Azidomethyl)-4-(1-azidovinyl)benzene (**1j**) was synthesized according the same procedure[1] from 1-(chloromethyl)-4-vinylbenzene as a result of simultaneous azidation of formed dibromide and nucleophilic substitution of chlorine atom. 2-Azidododec-1-ene was synthesized according to the literature through the reaction between styrenes and I_2/NaN_3 system followed by dehydroiodination with t-BuOK.[2]

Amines **2** were obtained from commercial suppliers and used without further purification.

Electrochemical cell

For the electrosynthesis glassy carbon and platinum plates from Russian commercial suppliers were used as electrodes (glassy carbon: СУ-2000: ТУ 1916-027-27208846-01; platinum grade: AISI 304): The reactions were performed in a common chemical tube:

Undivided electrochemical cell equipped with glassy carbon plate anode and platinum plate cathode with the reaction mixture during electrolysis under constant current conditions.

Before all electrochemical reactions the electrodes were put into 5 M solution of KOH and this mixture was electrolyzed for 10 minutes at j = 200 mA/cm^2. After that the polarity of electrodes was changed and the mixture was electrolyzed under these conditions again. After electrolysis the electrodes were washed with running water and then with acetone. All these procedures help to clean the electrodes from the impurities from the previous electrolysis.
The detailed electrochemical equipment was presented in our previous study [*Adv. Synth. Catal.* **2022**, *364 (6)*, 1098-1108].

Table S1. Detailed optimization of imidazole electrosynthesis.

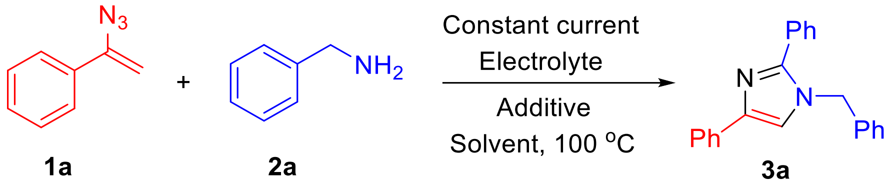

№	Cathode /Anode	Electrolyte (eq)	Additive (eq.)	Solvent	Current density, mA/cm^2	Electricity passed per **1a**, F/mol	Yield **3a** %
1	Pt/GC	**TBAI (1.0)**	-	DMF	10.0	4.0	24
2	Pt/GC	**KI (1.0)**	-	DMF	10.0	4.0	37
3	Pt/GC	**NH_4I (1.0)**	-	DMF	10.0	4.0	36
4	Pt/GC	**NH_4Br (1.0)**	-	DMF	10.0	4.0	26
5	Pt/GC	**$LiClO_4$ (1.0)**	-	DMF	10.0	4.0	22
7	Pt/GC	KI (1.0)	-	**DMA**	10.0	4.0	37
8	Pt/GC	KI (1.0)	-	**DMSO**	10.0	4.0	36
9[b]	Pt/GC	KI (1.0)	-	DMF	10.0	4.0	26
10[c]	Pt/GC	KI (1.0)	-	DMF	10.0	4.0	26
11[d]	Pt/GC	KI (1.0)	-	DMF	10.0	4.0	33
12	Pt/GC	KI (1.0)	***p*-TsOH·H_2O (0.5)**	DMF	10.0	4.0	38
13	Pt/GC	KI (1.0)	***p*-TsOH·H_2O (1.0)**	DMF	10.0	4.0	43
14	Pt/GC	KI (1.0)	***p*-TsOH·H_2O (2.0)**	DMF	10.0	4.0	48
15	Pt/GC	KI (1.0)	***p*-TsOH·H_2O (3.0)**	DMF	10.0	4.0	21
16	Pt/GC	KI (1.0)	**AcOH (2.0)**	DMF	10.0	4.0	40
17	Pt/GC	KI (1.0)	**HCOOH (2.0)**	DMF	10.0	4.0	32

18	Pt/GC	KI (1.0)	*p*-TsOH·H_2O (2.0)	DMF	10.0	**5.0**	41
19	Pt/GC	KI (1.0)	*p*-TsOH·H_2O (2.0)	DMF	**20.0**	4.0	39
20	Pt/GC	KI (1.0)	*p*-TsOH·H_2O (2.0)	DMF	**26.7**	4.0	26
21	Pt/GC	KI (1.0)	*p*-TsOH·H_2O (2.0)	DMF	20.0	**6.0**	55
22	Pt/GC	KI (1.0)	*p*-TsOH·H_2O (2.0)	DMF	20.0	**8.0**	30
23	Pt/GC	KI (1.0)	*p*-TsOH·H_2O (2.0)	DMF	-	-	7
24	-	KI (1.0)	*p*-TsOH·H_2O (2.0)	DMF	-	-	7
25	Pt/GC	KI (1.0)	*p*-TsOH·H_2O (2.0) *n*-Bu_4NClO_4 (1.0)	**PhCl**	20.0	6.0	18
26	Pt/GC	**KI (0.5)**	*p*-TsOH·H_2O (2.0)	DMF	20.0	6.0	25
27	Pt/GC	**KI (2.0)**	*p*-TsOH·H_2O (2.0)	DMF	20.0	6.0	53
28	Pt/GC	KI (1.0)	**H_2SO_4 (2.0)**	DMF	20.0	6.0	-
29	Pt/GC	KI (1.0)	**CH_3SO_3H (2.0)**	DMF	20.0	6.0	-
30	Pt/GC	KI (1.0)	**Amberlyst-15 (2.0)**	DMF	20.0	6.0	46
31	Pt/GC	KI (1.0)	**Lewatit MonoPlus SP-112-H (2.0)**	DMF	20.0	6.0	traces
32[e]	Pt/GC	KI (1.0)	*p*-TsOH·H_2O (2.0)	**CH_3CN**	20.0	6.0	7
33[e]	Pt/GC	KI (1.0)	*p*-TsOH·H_2O (2.0)	**MeOH**	20.0	6.0	traces
34[f]	Pt/GC	KI (1.0)	*p*-TsOH·H_2O (2.0)	DMF	20.0	6.0	61
35[g]	Pt/GC	KI (1.0)	*p*-TsOH·H_2O (2.0)	DMF	20.0	6.0	31
36	**GC/GC**	KI (1.0)	*p*-TsOH·H_2O (2.0)	DMF	20.0	6.0	15
37	**Pt/Pt**	KI (1.0)	*p*-TsOH·H_2O (2.0)	DMF	20.0	6.0	22
38	**Pt/C**	KI (1.0)	*p*-TsOH·H_2O (2.0)	DMF	20.0	6.0	36
39	**Cu/GC**	KI (1.0)	*p*-TsOH·H_2O (2.0)	DMF	20.0	6.0	35
40	**SS/GC**	KI (1.0)	*p*-TsOH·H_2O (2.0)	DMF	20.0	6.0	34
41	**Ni/GC**	KI (1.0)	*p*-TsOH·H_2O (2.0)	DMF	20.0	6.0	48

42	**GC/Pt**	KI (1.0)	*p*-TsOH·H_2O (2.0)	DMF	20.0	6.0	46

[a] **General reaction conditions:** undivided cell, glassy carbon plate anode / plathinum plate cathode (3 cm^2), constant current, **1a** (1.0 mmol, 145.2 mg), **2a** (2.0 mmol, 214.3 mg), solvent (10.0 mL), 100 °C, air atmosphere. [b] 20-25 °C, [c] 120 °C, [d] **2a** (4.0 mmol, 428.8 mg), [e] 25 °C, [f] 70 °C, [g] 50 °C

Experimental Procedures for Table S1.

Experimental Procedure for Table S1, entries 1-5.

An undivided cell was equipped with a glassy carbon anode (3 cm^2) and a platinum plate cathode (3 cm^2) and connected to a DC regulated power supply. The solution of (1-azidovinyl)benzene **1a** (1.0 mmol, 145.2 mg, 1.0 eq.), benzylamine **2a** (2.0 mmol, 214.3 mg, 2.0 eq.) and supporting electrolyte TBAI, KI, NH_4I, NH_4Br, $LiClO_4$ (1.0 mmol, 1.0 eq.) in 10 mL of DMF was electrolyzed using constant current conditions at 100 °C under magnetic stirring for 215 min with I = 30 mA. After that the reaction mixture was diluted with H_2O (30 ml) and washed with mixture of PE and ethyl acetate (1:1) (2×30 mL). Combined organic layer was washed with 0.3 M solution of $Na_2S_2O_3$ (2×10 mL), water (2×10 mL), dried over Na_2SO_4 and concentrated under reduced pressure using a rotary evaporator (15-20 mmHg), (bath temperature, ca. 30–40 °C). Product **3a** was isolated by chromatography on SiO_2 (PE:EtOAc = from 15:1 to 2:1).

Experimental Procedure for Table S1, entries 9-10.

An undivided cell was equipped with a glassy carbon anode (3 cm^2) and a platinum plate cathode (3 cm^2) and connected to a DC regulated power supply. The solution of (1-azidovinyl)benzene **1a** (1.0 mmol, 145.2 mg, 1.0 eq.), benzylamine **2a** (2.0 mmol, 214.3 mg, 2.0 eq.) and KI (1.0 mmol, 166.0 mg, 1.0 eq.) in 10 mL of DMF was electrolyzed using constant current conditions at 25 °C (entry 9) or 120 °C (entry 10) under magnetic stirring for 215 min with I = 30 mA. After that the reaction mixture was diluted with H_2O (30 ml) and washed with mixture of PE and ethyl acetate (1:1) (2×30 mL). Combined organic layer was washed with 0.3 M solution of $Na_2S_2O_3$ (2×10 mL), water (2×10 mL), dried over Na_2SO_4 and concentrated under reduced pressure using a rotary evaporator (15-20 mmHg), (bath temperature, ca. 30–40 °C). Product **3a** was isolated by chromatography on SiO_2 (PE:EtOAc = from 15:1 to 2:1).

Experimental Procedure for Table S1, entries 11.

An undivided cell was equipped with a glassy carbon anode (3 cm^2) and a platinum plate cathode (3 cm^2) and connected to a DC regulated power supply. The solution of (1-azidovinyl)benzene **1a** (1.0 mmol, 145.2 mg, 1.0 eq.), benzylamine **2a** (4.0 mmol, 428.8 mg, 4.0 eq.) KI (1.0 mmol, 166.0 mg, 1.0 eq.) in 10 mL of DMF was electrolyzed using constant current conditions at 100 °C under magnetic stirring for 215 min with I = 30 mA. After that the reaction mixture was diluted with H_2O (30 ml) and washed with mixture of PE and ethyl acetate (1:1) (2×30 mL). Combined organic layer was washed with 0.3 M solution of $Na_2S_2O_3$ (2×10 mL), water (2×10 mL), dried over Na_2SO_4 and concentrated under

reduced pressure using a rotary evaporator (15-20 mmHg), (bath temperature, ca. 30–40 °C). Product **3a** was isolated by chromatography on SiO_2 (PE:EtOAc = from 15:1 to 2:1).

Experimental Procedure for Table S1, entries 12-17.

An undivided cell was equipped with a glassy carbon anode (3 cm^2) and a platinum plate cathode (3 cm^2) and connected to a DC regulated power supply. The solution of (1-azidovinyl)benzene **1a** (1.0 mmol, 145.2 mg, 1.0 eq.), benzylamine **2a** (2.0 mmol, 214.3 mg, 2.0 eq.), additive *p*-TsOH·H_2O, AcOH, HCOOH (0.5-3.0 mmol, 0.5-3.0 eq.), and KI (1.0 mmol, 166.0 mg, 1.0 eq.) in 10 mL of DMF was electrolyzed using constant current conditions at 100 °C under magnetic stirring for 215 min with I = 30 mA. After that the reaction mixture was diluted with H_2O (30 ml) and washed with mixture of PE and ethyl acetate (1:1) (2×30 mL). Combined organic layer was washed with 0.3 M solution of $Na_2S_2O_3$ (2×10 mL), water (2×10 mL), dried over Na_2SO_4 and concentrated under reduced pressure using a rotary evaporator (15-20 mmHg), (bath temperature, ca. 30–40 °C). Product **3a** was isolated by chromatography on SiO_2 (PE:EtOAc = from 15:1 to 2:1).

Experimental Procedure for Table S1, entries 18-22.

An undivided cell was equipped with a glassy carbon anode (3 cm^2) and a platinum plate cathode (3 cm^2) and connected to a DC regulated power supply. The solution of (1-azidovinyl)benzene **1a** (1.0 mmol, 145.2 mg, 1.0 eq.), benzylamine **2a** (2.0 mmol, 214.3 mg, 2.0 eq.), *p*-TsOH·H_2O (2.0 mmol, 380.0 mg, 2.0 eq.), and KI (1.0 mmol, 166.0 mg, 1.0 eq.) in 10 mL of DMF was electrolyzed using constant current conditions at 100 °C under magnetic stirring for 270 min with I = 30 mA (entry 18); 110 min with I = 60 mA (entry 19); 80 min with I = 80 mA (entry 20); 160 min with I = 60 mA (entry 21) or 215 min with I = 60 mA (entry 22). After that the reaction mixture was diluted with H_2O (30 ml) and washed with mixture of PE and ethyl acetate (1:1) (2×30 mL). Combined organic layer was washed with 0.3 M solution of $Na_2S_2O_3$ (2×10 mL), water (2×10 mL), dried over Na_2SO_4 and concentrated under reduced pressure using a rotary evaporator (15-20 mmHg), (bath temperature, ca. 30–40 °C). Product **3a** was isolated by chromatography on SiO_2 (PE:EtOAc = from 15:1 to 2:1).

Experimental Procedure for Table S1, entries 23.

An undivided cell was equipped with a glassy carbon anode (3 cm^2) and a platinum plate cathode (3 cm^2). The solution of (1-azidovinyl)benzene **1a** (1.0 mmol, 145.2 mg, 1.0 eq.), benzylamine **2a** (2.0 mmol, 214.3 mg, 2.0 eq.), *p*-TsOH·H_2O (2.0 mmol, 380.0 mg, 2.0 eq.) and KI (1.0 mmol, 166.0 mg, 1.0 eq.) in 10 mL of DMF was stirred at 100 °C for 160 min. After that the reaction mixture was diluted with H_2O (30 ml) and washed with mixture of PE and ethyl acetate (1:1) (2×30 mL). Combined organic layer was washed with 0.3 M solution of $Na_2S_2O_3$ (2×10 mL), water (2×10 mL), dried over Na_2SO_4 and concentrated under reduced pressure using a rotary evaporator (15-20 mmHg), (bath temperature, ca. 30–40 °C). Product **3a** was isolated by chromatography on SiO_2 (PE:EtOAc = from 15:1 to 2:1).

Experimental Procedure for Table S1, entries 24.

The solution of (1-azidovinyl)benzene **1a** (1.0 mmol, 145.2 mg, 1.0 eq.), benzylamine **2a** (2.0 mmol, 214.3 mg, 2.0 eq.), *p*-TsOH·H_2O (2.0 mmol, 380.0 mg, 2.0 eq.), and KI (1.0 mmol, 166.0 mg, 1.0 eq.) in 10 mL of DMF was stirred at 100 °C for 160 min. After that the reaction mixture was diluted with H_2O

(30 ml) and washed with mixture of PE and ethyl acetate (1:1) (2×30 mL). Combined organic layer was washed with 0.3 M solution of $Na_2S_2O_3$ (2×10 mL), water (2×10 mL), dried over Na_2SO_4 and concentrated under reduced pressure using a rotary evaporator (15-20 mmHg), (bath temperature, ca. 30–40 °C). Product **3a** was isolated by chromatography on SiO_2 (PE:EtOAc = from 15:1 to 2:1).

Experimental Procedure for Table S1, entries 25.

An undivided cell was equipped with a glassy carbon anode (3 cm^2) and a platinum plate cathode (3 cm^2) and connected to a DC regulated power supply. The solution of (1-azidovinyl)benzene **1a** (1.0 mmol, 145.2 mg, 1.0 eq.), benzylamine **2a** (2.0 mmol, 214.3 mg, 2.0 eq.), *p*-TsOH·H_2O (2.0 mmol, 380.0 mg, 2.0 eq.), KI (1.0 mmol, 166.0 mg, 1.0 eq.), and *n*-Bu_4NClO_4 (1.0 mmol, 314.9 mg, 1.0 eq.) in 10 mL of chlorobenzene was electrolyzed using constant current conditions at 100 °C under magnetic stirring for 160 min with I = 60 mA. After that the reaction mixture was diluted with H_2O (30 ml) and washed with mixture of PE and ethyl acetate (1:1) (2×30 mL). Combined organic layer was washed with 0.3 M solution of $Na_2S_2O_3$ (2×10 mL), water (2×10 mL), dried over Na_2SO_4 and concentrated under reduced pressure using a rotary evaporator (15-20 mmHg), (bath temperature, ca. 30–40 °C). Product **3a** was isolated by chromatography on SiO_2 (PE:EtOAc = from 15:1 to 2:1).

Experimental Procedure for Table S1, entries 26, 27.

An undivided cell was equipped with a glassy carbon anode (3 cm^2) and a platinum plate cathode (3 cm^2) and connected to a DC regulated power supply. The solution of (1-azidovinyl)benzene **1a** (1.0 mmol, 145.2 mg, 1.0 eq.), benzylamine **2a** (2.0 mmol, 214.3 mg, 2.0 eq.), *p*-TsOH·H_2O (2.0 mmol, 380.0 mg, 2.0 eq.), and KI (0.5 – 2.0 mmol, 0.5 – 2.0 eq.) in 10 mL of DMF was electrolyzed using constant current conditions at 100 °C under magnetic stirring for 160 min with I = 60 mA. After that the reaction mixture was diluted with H_2O (30 ml) and washed with mixture of PE and ethyl acetate (1:1) (2×30 mL). Combined organic layer was washed with 0.3 M solution of $Na_2S_2O_3$ (2×10 mL), water (2×10 mL), dried over Na_2SO_4 and concentrated under reduced pressure using a rotary evaporator (15-20 mmHg), (bath temperature, ca. 30–40 °C). Product **3a** was isolated by chromatography on SiO_2 (PE:EtOAc = from 15:1 to 2:1).

Experimental Procedure for Table S1, entries 28-31.

An undivided cell was equipped with a glassy carbon anode (3 cm^2) and a platinum plate cathode (3 cm^2) and connected to a DC regulated power supply. The solution of (1-azidovinyl)benzene **1a** (1.0 mmol, 145.2 mg, 1.0 eq.), benzylamine **2a** (2.0 mmol, 214.3 mg, 2.0 eq.), additive H_2SO_4, CH_3SO_3H, Amberlist-15, or Lewatit MonoPlus SP-112-H (2.0 mmol, 2.0 eq.), and KI (1.0 mmol, 166.0 mg, 1.0 eq.) in 10 mL of DMF was electrolyzed using constant current conditions at 100 °C under magnetic stirring for 160 min with I = 60 mA. After that the reaction mixture was diluted with H_2O (30 ml) and washed with mixture of PE and ethyl acetate (1:1) (2×30 mL). Combined organic layer was washed with 0.3 M solution of $Na_2S_2O_3$ (2×10 mL), water (2×10 mL), dried over Na_2SO_4 and concentrated under reduced pressure using a rotary evaporator (15-20 mmHg), (bath temperature, ca. 30–40 °C). Product **3aa** was not detected (entries 28-29). Product **3a** was isolated by chromatography on SiO_2 (PE:EtOAc = from 15:1 to 2:1) (entry 30).

Experimental Procedure for Table S1, entries 32,33.

An undivided cell was equipped with a glassy carbon anode (3 cm^2) and a platinum plate cathode (3 cm^2) and connected to a DC regulated power supply. The solution of (1-azidovinyl)benzene **1a** (1.0 mmol, 145.2 mg, 1.0 eq.), benzylamine **2a** (2.0 mmol, 214.3 mg, 2.0 eq.), *p*-TsOH·H_2O (2.0 mmol, 380.0 mg, 2.0 eq.), and KI (0.5 – 2.0 mmol, 0.5 – 2.0 eq.) in 10 mL of CH_3CN (entry 32) or MeOH (entry 33) was electrolyzed using constant current conditions at 25 °C under magnetic stirring for 160 min with I = 60 mA. After that the reaction mixture was diluted with H_2O (30 ml) and washed with mixture of PE and ethyl acetate (1:1) (2×30 mL). Combined organic layer was washed with 0.3 M solution of $Na_2S_2O_3$ (2×10 mL), water (2×10 mL), dried over Na_2SO_4 and concentrated under reduced pressure using a rotary evaporator (15-20 mmHg), (bath temperature, ca. 30–40 °C). Product **3a** was isolated by chromatography on SiO_2 (PE:EtOAc = from 15:1 to 2:1).

Experimental Procedure for Table S1, entries 34, 35.

An undivided cell was equipped with a glassy carbon anode (3 cm^2) and a platinum plate cathode (3 cm^2) and connected to a DC regulated power supply. The solution of (1-azidovinyl)benzene **1a** (1.0 mmol, 145.2 mg, 1.0 eq.), benzylamine **2a** (2.0 mmol, 214.3 mg, 2.0 eq.), *p*-TsOH·H_2O (2.0 mmol, 380.0 mg, 2.0 eq.), and KI (1.0 mmol, 166.0 mg, 1.0 eq.) in 10 mL of DMF was electrolyzed using constant current conditions at 70 °C (entry 34) or 50 °C (entry 35) under magnetic stirring for 160 min with I = 60 mA. After that the reaction mixture was diluted with H_2O (30 ml) and washed with mixture of PE and ethyl acetate (1:1) (2×30 mL). Combined organic layer was washed with 0.3 M solution of $Na_2S_2O_3$ (2×10 mL), water (2×10 mL), dried over Na_2SO_4 and concentrated under reduced pressure using a rotary evaporator (15-20 mmHg), (bath temperature, ca. 30–40 °C). Product **3a** was isolated by chromatography on SiO_2 (PE:EtOAc = from 15:1 to 2:1).

Experimental Procedure for Table S1, entries 36-42

An undivided cell was equipped with a glassy carbon electrodes (3 cm^2 for each electrode, entry 36), or platinum electrodes (3 cm^2 for each electrode, entry 37), a graphite plate anode (3 cm^2 and a platinum plate cathode (3 cm^2) (entry 38); a glassy carbon anode (3 cm^2) and a copper plate cathode (3 cm^2) (entry 39); a glassy carbon anode (3 cm^2) and stainless steel plate cathode (3 cm^2) (entry 40); a glassy carbon anode (3 cm^2) and nickel plate cathode (3 cm^2) (entry 41); a platinum plate anode (3 cm^2) and glassy carbon anode (3cm^2) (entry 42) and connected to a DC regulated power supply. The solution of (1-azidovinyl)benzene **1a** (1.0 mmol, 145.2 mg, 1.0 eq.), benzylamine **2a** (2.0 mmol, 214.3 mg, 2.0 eq.), *p*-TsOH·H_2O (2.0 mmol, 380.0 mg, 2.0 eq.), and KI (1.0 mmol, 166.0 mg, 1.0 eq.) in 10 mL of DMF was electrolyzed using constant current conditions at 70 °C under magnetic stirring for 160 min with I = 60 mA. After that the reaction mixture was diluted with H_2O (30 ml) and washed with mixture of PE and ethyl acetate (1:1) (2×30 mL). Combined organic layer was washed with 0.3 M solution of $Na_2S_2O_3$ (2×10 mL), water (2×10 mL), dried over Na_2SO_4 and concentrated under reduced pressure using a rotary evaporator (15-20 mmHg), (bath temperature, ca. 30–40 °C). Product **3a** was isolated by chromatography on SiO_2 (PE:EtOAc = from 15:1 to 2:1).

General Experimental Procedure for Schemes 2, 3.

An undivided cell was equipped with a glassy carbon anode (3 cm^2) and a platinum plate cathode (3 cm^2) and connected to a DC regulated power supply. The solution of **1a** (1.0 mmol, 1.0 eq.), **2a** (2.0 mmol, 2.0 eq.), *p*-TsOH·H_2O (2.0 mmol, 380.0 mg, 2.0 eq.), and KI (1.0 mmol, 166.0 mg, 1.0 eq.) in 10 mL of DMF was electrolyzed using constant current conditions at 70 °C under magnetic stirring for 160 min with I = 60 mA (*j* = 20 mA/cm^2). After that the reaction mixture was diluted with H_2O (30 ml) and washed with mixture of PE and ethyl acetate (1:1) (2×30 mL). Combined organic layer was washed with 0.3 M solution of $Na_2S_2O_3$ (2×10 mL), water (2×10 mL), dried over Na_2SO_4 and concentrated under reduced pressure using a rotary evaporator (15-20 mmHg), (bath temperature, ca. 30–40 °C). Product **3** was isolated by chromatography on SiO_2.

1-benzyl-2,4-diphenyl-1*H*-imidazole (3a) [3]

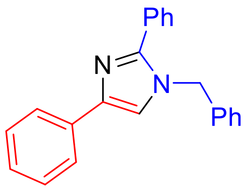

Yellow solid. Yield 61% (189.7 mg, 0.61 mmol, PE/EtOAc = from 15:1 to 2:1 as eluent), mp = 123-124 °C (lit.[3] mp = 123-124 °C). R_f = 0.36 (PE:EtOAc = 5:1).
^{1}H NMR (300.13 MHz, $CDCl_3$, δ): 7.88 (d, *J* = 7.4 Hz, 2H), 7.68 – 7.58 (m, 2H), 7.51 – 7.43 (m, 3H), 7.43 – 7.35 (m, 5H), 7.33 – 7.27 (m, 2H), 7.23 – 7.13 (m, 2H), 5.26 (s, 2H).
$^{13}C\{^1H\}$ NMR (75.48 MHz, $CDCl_3$, δ): 148.7, 141.6, 136.9, 134.2, 130.5, 129.1, 128.7, 128.6, 128.0, 126.9, 126.7, 125.0, 116.9, 50.5.
HRMS (ESI-TOF) m/z $[M+H]^+$. Calcd for $[C_{22}H_{19}N_2]^+$: 311.1543. Found: 311.1543.
IR (KBr): 3469, 3034, 1651, 1474, 1446, 1398, 772, 736, 697 cm^{-1}.

1- benzyl-2-phenyl-4-(p-tolyl)-1*H*-imidazole (3b) [3]

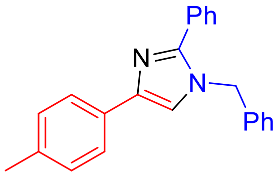

Yellow solid. Yield 52% (168.5 mg, 0.52 mmol, PE/EtOAc = from 15:1 to 2:1 as eluent), mp = 140-142 °C (lit.[3] mp = 138-140 °C). R_f = 0.37 (PE:EtOAc = 5:1).
^{1}H NMR (300.13 MHz, $CDCl_3$, δ): 7.75 (d, *J* = 8.0 Hz, 2H), 7.68 – 7.58 (m, 2H), 7.47 – 7.38 (m, 3H), 7.37 – 7.28 (m, 3H), 7.24 – 7.10 (m, 5H), 5.21 (s, 2H), 2.36 (s, 3H)
$^{13}C\{^1H\}$ NMR (75.48 MHz, $CDCl_3$, δ): 148.5, 141.6, 137.0, 136.6, 131.2, 130.5, 129.3, 129.2, 129.1, 128.7, 128.1, 126.8, 125.0, 116.5, 50.6, 21.3.
HRMS (ESI-TOF) m/z $[M+H]^+$. Calcd for $[C_{23}H_{21}N_2]^+$: 325.1699. Found: 325.1696.
IR (KBr): 3542, 3498, 3468, 3438, 3066, 3029, 2957, 2924, 2855, 1729, 1644, 1646, 1273, 1178, 822, 763, 731, 698 cm^{-1}.

1-benzyl-4-(4-(tert-butyl)phenyl)-2-phenyl-1*H*-imidazole (3c) [3]

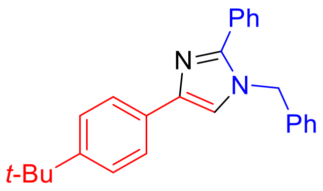

Yellow liquid. Yield 64% (234.6 mg, 0.64 mmol, PE/EtOAc = from 15:1 to 2:1 as eluent). R_f = 0.25 (PE:EtOAc = 5:1).

^{1}H NMR (300.13 MHz, $CDCl_3$, δ): 7.80 (d, *J* = 8.3 Hz, 2H), 7.68 – 7.58 (m, 2H), 7.46 – 7.37 (m, 5H), 7.37 – 7.29 (m, 3H), 7.23 (s, 1H), 7.13 (d, *J* = 6.6 Hz, 2H), 5.22 (s, 2H), 1.36 (s, 9H).

^{13}C{^{1}H} NMR (75.48 MHz, $CDCl_3$, δ): 149.9, 148.5, 141.7, 137.1, 131.3, 130.6, 129.2, 129.1, 129.0, 128.7, 128.0, 126.7, 125.5, 124.8, 116.6, 50.6, 34.6, 31.5.

HRMS (ESI-TOF) m/z $[M+H]^+$. Calcd for $[C_{26}H_{27}N_2]^+$: 367.2169. Found: 367.2165.

IR (KBr): 3465, 3444, 3142, 3116, 3064, 3029, 2957, 2866, 1604, 1495, 1470, 1451, 1415, 1365, 1202, 836, 773, 730, 699, 522 cm^{-1}.

1-benzyl-2-phenyl-4-(m-tolyl)-1*H*-imidazole (3d) [4]

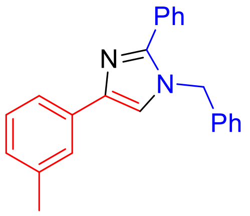

Yellow solid. Yield 44% (142.7 mg, 0.44 mmol, PE/EtOAc = from 15:1 to 2:1 as eluent), mp = 124-126 °C (lit.[4] mp = 125-126 °C). R_f = 0.23 (PE:EtOAc = 5:1).

^{1}H NMR (300.13 MHz, $CDCl_3$, δ): 7.78 (s, 1H), 7.69 – 7.59 (m, 3H), 7.47 – 7.38 (m, 3H), 7.39 – 7.27 (m, 4H), 7.24 (s, 1H), 7.17 – 7.07 (m, 3H), 5.18 (s, 2H), 2.41 (s, 3H).

^{13}C{^{1}H} NMR (75.48 MHz, $CDCl_3$, δ): 148.5, 141.5, 138.1, 136.9, 133.9, 130.4, 128.98, 128.95, 128.6, 128.4, 127.9, 127.6, 126.6, 125.6, 122.0, 116.9, 50.4, 21.5.

HRMS (ESI-TOF) m/z $[M+H]^+$. Calcd for $[C_{23}H_{21}N_2]^+$: 325.1699. Found: 325.1695.

IR (KBr): 3471, 3134, 3030, 2952, 2918, 1604, 1449, 1402, 1359, 753, 696 cm^{-1}.

1-benzyl-4-(4-methoxyphenyl)-2-phenyl-1*H*-imidazole (3e) [4]

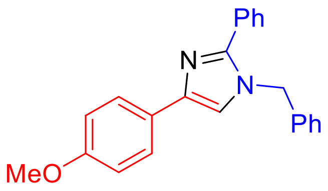

Yellow liquid. Yield 34% (115.6 mg, 0.34 mmol, PE/EtOAc = from 10:1 to 2:1 as eluent). R_f = 0.13 (PE:EtOAc = 5:1).

^{1}H NMR (300.13 MHz, $CDCl_3$, δ): 7.78 (d, *J* = 8.6 Hz, 2H), 7.67 – 7.57 (m, 2H), 7.47 – 7.39 (m, 3 H), 7.38 – 7.30 (m, 3H), 7.19 – 7.10 (m, 3H), 6.92 (d, *J* = 8.6 Hz, 2H), 5.21 (s, 2H), 3.82 (s, 3H).

^{13}C{^{1}H} NMR (75.48 MHz, $CDCl_3$, δ): 159.0, 148.4, 141.3, 136.9, 130.2, 129.21, 129.15, 128.8, 128.1, 126.8, 126.7, 126.4, 115.9, 114.1, 55.4, 50.7.

HRMS (ESI-TOF) m/z $[M+H]^+$. Calcd for $[C_{23}H_{21}N_2O]^+$: 341.1648. Found: 341.1649.

IR (KBr): 3446, 3427, 3126, 3103, 3060, 3030, 2959, 2835, 1612, 1559, 1497, 1453, 1248, 1172, 1024, 833, 765, 698, 526 cm^{-1}.

1-benzyl-4-(4-fluorophenyl)-2-phenyl-1*H*-imidazole (3f) [3]

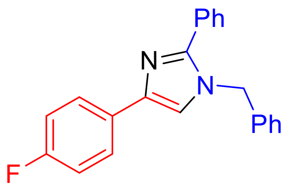

Yellow solid. Yield 53% (174.0 mg, 0.53 mmol, PE/EtOAc = from 10:1 to 2:1 as eluent), mp = 105-107 °C (lit.[3] mp = 106-107 °C). R_f = 0.67 (PE:EtOAc = 2:1).

^{1}H NMR (300.13 MHz, $CDCl_3$, δ): 7.87 – 7.75 (m, 2H), 7.66 – 7.57 (m, 2H), 7.46 – 7.39 (m, 3H), 7.38 – 7.31 (m, 3H), 7.19 (s, 1H), 7.17 – 7.10 (m, 2H), 7.09 – 7.00 (m, 2H), 5.20 (s, 2H).

^{13}C{^{1}H} NMR (75.48 MHz, $CDCl_3$, δ): 162.0 (d, *J* = 245.4 Hz), 148.7, 140.7, 136.8, 130.4 (d, *J* = 2.8 Hz), 129.12, 129.07, 129.0, 128.7, 128.1, 126.7, 126.60 (d, *J* = 7.9 Hz), 116.5, 115.4 (d, *J* = 21.5 Hz), 50.55.

HRMS (ESI-TOF) m/z $[M+H]^+$. Calcd for $[C_{22}H_{18}FN_2]^+$: 329.1449. Found: 329.1443.

IR (KBr): 3458, 3059, 3032, 2357, 1644, 1495, 1348, 1218, 1156, 840, 761, 731, 695, 583, 519 cm^{-1}.

1-benzyl-4-(4-bromophenyl)-2-phenyl-1*H*-imidazole (3g) [3]

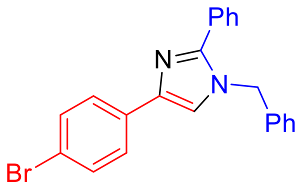

Yellow solid. Yield 56% (218.0 mg, 0.56 mmol, PE/EtOAc = from 10:1 to 2:1 as eluent), mp = 166-167 °C (lit.[3] mp = 164-166 °C). R_f = 0.71 (PE:EtOAc = 2:1).

^{1}H NMR (300.13 MHz, $CDCl_3$, δ): 7.72 (d, *J* = 8.4 Hz, 2H), 7.66 – 7.57 (m, 2H), 7.48 (d, *J* = 8.4 Hz, 2H), 7.46 – 7.40 (m, 3H), 7.39 – 7.30 (m, 3H), 7.22 (s, 1H), 7.17 – 7.08 (m, 2H), 5.19 (s, 2H).

^{13}C{^{1}H} NMR (75.48 MHz, $CDCl_3$, δ): 148.8, 140.4, 136.7, 133.1, 131.6, 130.2, 129.2, 129.1, 129.0, 128.7, 128.1, 126.8, 126.6, 120.5, 117.1, 50.6.

HRMS (ESI-TOF) m/z $[M+H]^+$. Calcd for $[C_{22}H_{18}BrN_2]^+$: 389.0648, 391.0628. Found: 389.0645, 391.0628.

IR (KBr): 3472, 3129, 3059, 3025, 2977, 2952, 1599, 1550, 1478, 1413, 1188, 1070, 946, 830, 767, 701, 506 cm^{-1}.

1-benzyl-4-(3-bromophenyl)-2-phenyl-1*H*-imidazole (3h)

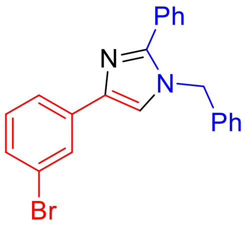

Yellow solid. Yield 42% (163.5 mg, 0.42 mmol, PE/EtOAc = from 10:1 to 2:1 as eluent), mp = 104-106 °C. R_f = 0.69 (PE:EtOAc = 2:1).

^{1}H NMR (300.13 MHz, $CDCl_3$, δ): 8.02 (s, 1H), 7.75 (d, *J* = 7.7 Hz, 1H), 7.66 – 7.56 (m, 2 H), 7.48 – 7.39 (m, 3H), 7.38 – 7.29 (m, 4H), 7.25 – 7.17 (m, 2H), 7.17 – 7.09 (m, 2H), 5.21 (s, 2H).

^{13}C{^{1}H} NMR (75.48 MHz, $CDCl_3$, δ): 148.9, 140.2, 136.7, 136.3, 130.3, 130.2, 129.7, 129.3, 129.2, 129.1, 128.8, 128.2, 128.0, 126.8, 123.5, 122.9, 117.5, 50.7.

HRMS (ESI-TOF) m/z $[M+H]^+$. Calcd for $[C_{22}H_{18}BrN_2]^+$: 389.0648. Found: 389.0645.

IR (KBr): 3477, 3129, 3062, 3031, 2951, 1600, 1566, 1468, 1450, 1205, 1072, 956, 872, 735, 697, 461 cm^{-1}.

1-benzyl-4-(2-chlorophenyl)-2-phenyl-1*H*-imidazole (3i) [3]

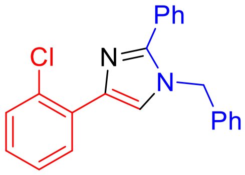

Yellow liquid. Yield 36% (124.2 mg, 0.36 mmol, PE/EtOAc = from 15:1 to 2:1 as eluent). R_f = 0.31 (PE:EtOAc = 5:1).
^{1}H NMR (300.13 MHz, $CDCl_3$, δ): 8.35 (dd, *J* = 7.9, 1.6 Hz, 1H), 7.77 (s, 1H), 7.67 – 7.58 (m, 2H), 7.46 – 7.39 (m, 4H), 7.38 – 7.30 (m, 4H), 7.22 – 7.10 (m, 3H), 5.26 (s, 2H).
^{13}C{^{1}H} NMR (75.48 MHz, $CDCl_3$, δ): 147.8, 137.6, 136.9, 132.5, 130.9, 130.4, 130.2, 129.8, 129.1, 129.1, 128.7, 128.0, 127.5, 126.9, 126.6, 121.7, 50.6.
HRMS (ESI-TOF) m/z $[M+H]^+$. Calcd for $[C_{22}H_{18}ClN_2]^+$: 345.1153. Found: 345.1149.
IR (KBr): 3449, 3147, 3057, 3031, 1473, 1451, 1426, 1351, 1182, 1047, 764, 736, 700, 560 cm^{-1}.

4-(4-(azidomethyl)phenyl)-1-benzyl-2-phenyl-1*H*-imidazole (3j)

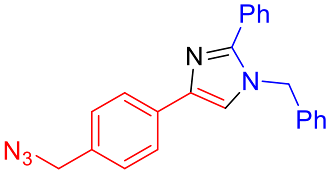

Yellow liquid. Yield 30% (109.6 mg, 0.30 mmol, PE/EtOAc = from 15:1 to 2:1 as eluent). R_f = 0.55 (PE:EtOAc = 2:1).
^{1}H NMR (300.13 MHz, $CDCl_3$, δ): 7.86 (d, *J* = 8.2 Hz, 2H), 7.66 – 7.56 (m, 2H), 7.48 – 7.39 (m, 3H), 7.38 – 7.28 (m, 5H), 7.26 (s, 1H), 7.18 – 7.08 (m, 2H), 5.20 (s, 2H), 4.32 (s, 2H).
^{13}C{^{1}H} NMR (75.48 MHz, $CDCl_3$, δ): 148.8, 141.0, 136.8, 134.2, 133.7, 130.4, 129.14, 129.07, 128.7, 128.6, 128.1, 126.8, 125.4, 117.2, 54.8, 50.6
HRMS (ESI-TOF) m/z $[M+H]^+$. Calcd for $[C_{23}H_{20}N_5]^+$: 366.1713. Found: 366.1712.
IR (KBr): 3108, 3063, 3031, 2929, 2875, 2098, 1613, 1498, 1471, 1452, 1422, 1355, 1249, 1181, 1075, 1021, 948, 848, 771, 732, 699 cm^{-1}.

1-(4-methoxybenzyl)-2-(4-methoxyphenyl)-4-phenyl-1*H*-imidazole (3l) [3]

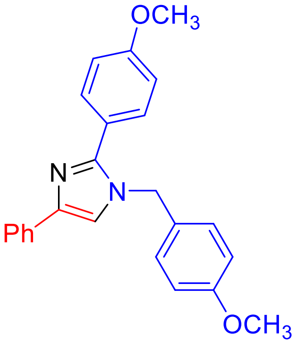

Yellow liquid. Yield 35% (129.7 mg, 0.35 mmol, PE/EtOAc = 5:1 as eluent). R_f = 0.38 (PE:EtOAc = 2:1).
^{1}H NMR (300.13 MHz, $CDCl_3$, δ): 7.80 (d, *J* = 7.3 Hz, 2H), 7.57 – 7.47 (m, 2H), 7.33 (t, *J* = 7.6 Hz, 2H), 7.24 – 7.14 (m, 2H), 7.04 (d, *J* = 8.6 Hz, 2H), 6.96 – 6.89 (m, 2H), 6.88 – 6.78 (m, 2H), 5.09 (s, 2H), 3.81 (s, 3H), 3.77 (s, 1H).
^{13}C{^{1}H} NMR (75.48 MHz, $CDCl_3$, δ): 160.3, 159.4, 148.5, 141.3, 134.3, 130.5, 129.0, 128.6, 128.2, 126.8, 125.0, 123.2, 116.5, 114.5, 114.1, 55.44, 55.42, 50.1.
HRMS (ESI-TOF) m/z $[M+H]^+$. Calcd for $[C_{24}H_{23}N_2O_2]^+$: 371.1754. Found: 371.1751.
IR (KBr): 3130, 3060, 3033, 3002, 2957, 2935, 2834, 1611, 1514, 1485, 1457, 1295, 1253, 1177, 1029, 838, 735, 697, 611, 518 cm^{-1}.

1-(4-chlorobenzyl)-2-(4-chlorophenyl)-4-phenyl-1*H*-imidazole (3m) [3]

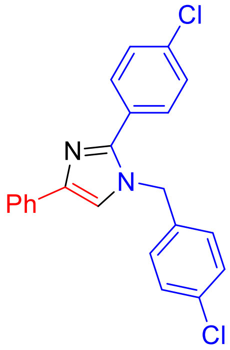

Yellow liquid. Yield 55% (208.6 mg, 0.55 mmol, PE/EtOAc = from 15:1 to 2:1 as eluent). R_f = 0.29 (PE:EtOAc = 5:1).
^{1}H NMR (300.13 MHz, $CDCl_3$, δ): 7.80 (d, *J* = 7.5 Hz, 2H), 7.54 – 7.44 (m, 2H), 7.42 – 7.35 (m, 4H), 7.34 – 7.27 (m, 2H), 7.26 – 7.18 (m, 2H), 7.01 (d, *J* = 8.3 Hz, 2H), 5.12 (s, 2H).
^{13}C{^{1}H} NMR (75.48 MHz, $CDCl_3$, δ): 147.4, 142.0, 135.3, 135.1, 134.1, 133.8, 130.2, 129.4, 129.0, 128.8, 128.7, 128.0, 127.2, 125.0, 117.1, 50.0.
HRMS (ESI-TOF) m/z $[M+H]^+$. Calcd for $[C_{22}H_{17}Cl_2N_2]^+$: 379.0763. Found: 379.0760.
IR (KBr): 3130, 3062, 3032, 2931, 1896, 1606, 1489, 1450, 1411, 1180, 1092, 1014, 947, 910, 837, 732. 696, 488 cm^{-1}.

1-(2-chlorobenzyl)-2-(2-chlorophenyl)-4-phenyl-1*H*-imidazole (3n) [4]

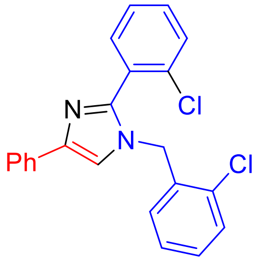

Yellow solid. Yield 40% (151.7 mg, 0.40 mmol, PE/EtOAc = from 15:1 to 2:1 as eluent), mp =134-136 °C (lit.[4] mp = 135-136 °C). R_f = 0.22 (PE:EtOAc = 5:1).
^{1}H NMR (300.13 MHz, $CDCl_3$, δ): 7.81 (d, *J* = 7.4 Hz, 2H), 7.51 – 7.42 (m, 2H), 7.40 – 7.27 (m, 5H), 7.25 – 7.11 (m, 4H), 6.97 – 6.87 (m, 1H), 5.08 (s, 2H).
^{13}C{^{1}H} NMR (75.48 MHz, $CDCl_3$, δ): 145.9, 141.5, 134.7, 133.9, 133.8, 133.1, 132.8, 131.1, 129.9, 129.7, 129.5, 129.3, 128.6, 127.3, 127.0, 126.9, 124.9, 115.9, 48.2.
HRMS (ESI-TOF) m/z $[M+H]^+$. Calcd for $[C_{22}H_{17}Cl_2N_2]^+$: 379.0763. Found: 379.0761.
IR (KBr): 3138, 3057, 2937, 2854, 1604, 1446, 1405, 1382, 1336, 1192, 1028, 948, 915, 753, 697, 506 cm^{-1}.

1-(4-fluorobenzyl)-2-(4-fluorophenyl)-4-phenyl-1*H*-imidazole (3o) [4]

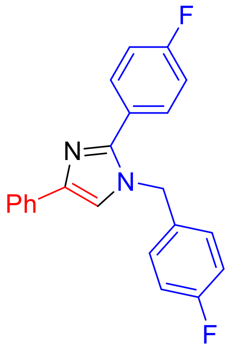

Yellow liquid. Yield 40% (138.6 mg, 0.40 mmol, PE/EtOAc = from 10:1 to 2:1 as eluent). R_f = 0.62 (PE:EtOAc = 2:1).
^{1}H NMR (300.13 MHz, $CDCl_3$, δ): 7.87 – 7.77 (m, 2H), 7.60 – 7.49 (m, 2H), 7.36 (t, *J* = 7.6 Hz, 2H), 7.28 – 7.22 (m, 1H), 7.21 (s, 1H), 7.16 – 7.09 (m, 1H), 7.09 – 6.97 (m, 5H), 5.12 (s, 2H).
^{13}C{^{1}H} NMR (75.48 MHz, $CDCl_3$, δ): 164.5 (d, *J* = 247.0 Hz), 161.24 (d, *J* = 245.2Hz), 147.6, 141.7, 133.9, 132.4 (d, *J* = 3.3 Hz), 131.0 (d, *J* = 8.4 Hz), 128.7, 128.5 (d, *J* = 8.2 Hz), 127.1, 126.6 (d, *J* = 3.7 Hz), 125.0, 116.8, 116.1 (d, *J* = 19.4 Hz), 115.8 (d, *J* = 19.7 Hz), 49.9.
^{19}F NMR (282 MHz, $CDCl_3$) δ -112.31, -114.50.
HRMS (ESI-TOF) m/z $[M+H]^+$: Calcd for $[C_{22}H_{17}F_2N_2]^+$: 347.1354. Found: 347.1354.
IR (KBr): 3129, 3065, 3038, 2932, 1607, 1511, 1485, 1451, 1419, 1226, 1159, 1097, 1015, 947, 910, 843, 733, 697, 607, 505 cm^{-1}.

1-(3,4-dimethoxybenzyl)-2-(3,4-dimethoxyphenyl)-4-phenyl-1*H*-imidazole (3p) [3]

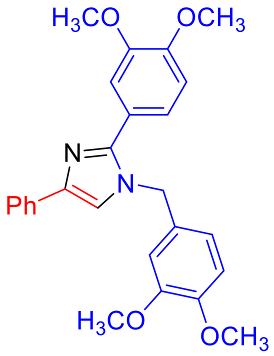

Yellow solid. Yield 38% (163.6 mg, 0.38 mmol, PE/EtOAc = 3:1 as eluent), mp = 181-183 °C (lit.[3] mp = 182-184 °C). R_f = 0.15 (PE:EtOAc = 2:1).
^{1}H NMR (300.13 MHz, $CDCl_3$, δ): 7.84 (d, *J* = 7.4 Hz, 2H), 7.36 (t, *J* = 7.4 Hz, 2 H), 7.28 – 7.17 (m, 3H), 7.13 (d, *J* = 8.6 Hz, 1H), 6.94 – 6.79 (m, 2H), 6.74 – 6.66 (m, 1H), 6.63 (s, 1H), 5.16 (s, 2H), 3.90 (s, 3H), 3.87 (s, 3H), 3.82 (s, 3H), 3.80 (s, 3H).
^{13}C{^{1}H} NMR (75.48 MHz, $CDCl_3$, δ): 149.9, 149.5, 149.1, 148.9, 148.4, 141.1, 133.9, 129.4, 128.6, 126.9, 125.0, 123.0, 121.6, 119.1, 116.7, 112.5, 111.6, 111.1, 109.9, 56.0, 55.9, 50.4
HRMS (ESI-TOF) m/z $[M+H]^+$. Calcd for $[C_{26}H_{27}N_2O_4]^+$: 431.1965. Found: 431.1969.
IR (KBr): 3453, 3130, 3099, 3012, 2959,2936,2835, 1606, 1515, 1442, 1320, 1261, 1244, 1141, 1025, 812, 765, 723, 696 cm^{-1}.

2-(furan-2-yl)-1-(furan-2-ylmethyl)-4-phenyl-1*H*-imidazole (3q) [3]

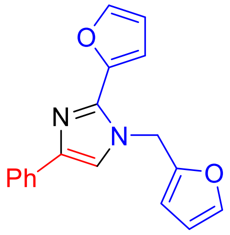

Yellow liquid. Yield 38% (110.3 mg, 0.38 mmol, PE/EtOAc = from 10:1 to 2:1 as eluent). R_f = 0.55 (PE:EtOAc = 2:1).
^{1}H NMR (300.13 MHz, $CDCl_3$, δ): 7.81 (d, *J* = 7.4 Hz, 2H), 7.58 – 7.51 (m, 1H), 7.40 – 7.30 (m, 3H), 7.25 – 7.20 (m, 2H), 6.96 (d, *J* = 3.4 Hz, 1H), 6.58 – 6.49 (m, 1H), 6.36 – 6.31 (m, 1H), 6.31 – 6.26 (m, 1H), 5.36 (s, 2H).
^{13}C{^{1}H} NMR (75.48 MHz, $CDCl_3$, δ): 149.3, 145.4, 143.1, 142.9, 141.7, 139.1, 133.7, 128.6, 127.0, 125.1, 116.7, 111.7, 110.7, 110.4, 109.1, 44.0.
HRMS (ESI-TOF) m/z $[M+H]^+$.Calcd for $[C_{18}H_{15}N_2O_2]^+$: 291.1128. Found: 291.1125.

IR (KBr): 3124, 3061, 3032, 2928, 2853, 1678, 1606, 1482, 1446, 1343, 1222, 1185, 1149, 1073, 1011, 948, 909, 885, 816, 736, 696, 596, 504 cm^{-1}.

3-(4-phenyl-1-(pyridin-3-ylmethyl)-1*H*-imidazol-2-yl)pyridine (3r)

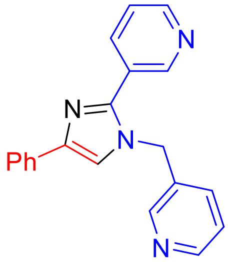

Yellow liquid. Yield 30% (93.7 mg, 0.30 mmol, PE/EtOAc = 1:1 as eluent). R_f = 0.10 (PE:EtOAc = 1:1).
^1H NMR (300.13 MHz, $CDCl_3$, δ): 8.80 (s, 1H), 8.61 (d, *J* = 4.7, 1H), 8.52 (d, *J* = 4.7, 1H), 8.40 (s, 1H), 7.88 (d, *J* = 7.8 Hz, 1H), 7.78 (d, *J* = 7.9 Hz, 2H), 7.40 – 7.29 (m, 4H), 7.29 – 7.19 (m, 3H), 5.20 (s, 2H).
$^{13}C\{^1H\}$ NMR (75.48 MHz, $CDCl_3$, δ): 150.1, 149.7, 149.3, 148.2, 145.4, 142.6, 136.4, 134.3, 133.4, 131.9, 128.7, 127.3, 126.5, 125.0, 124.0, 123.6, 117.3, 48.4.
HRMS (ESI-TOF) m/z $[M+H]^+$. Calcd for $[C_{20}H_{17}N_4]^+$: 313.1448. Found: 313.1440.
IR (KBr): 3386, 3127, 3059, 3035, 2934, 2219, 1606, 1575, 1481, 1450, 1426, 1193, 1090, 1027, 912, 816, 731, 644, 507 cm^{-1}.

CV study

Cyclic voltammetry (CV) was implemented on an IPC-Pro M computer-assisted potentiostat manufactured by «Econix» (scan rate error 1.0%). The starting potential was set to 0.25 mV, and the initial sweep was carried out in the positive (anode) region at a rate of 100 mV/s. Analyzed solutions were prepared in acetonitrile and contained *n*-Bu_4NBF_4 (0.1 M) as an supporting electrolyte and analyte (0.05 M). The experiments were performed in a 10 mL fiveneck glass conic electrochemical cell with a water jacket for thermostatting. CV curves were recorded using a three-electrode scheme. In a typical case, 10 mL of a solution was utilized. The working electrode was a disc glassy-carbon electrode (d= 3 mm, surface area ~0.07 cm^2). A platinum wire served as an auxiliary electrode. An Ag/$AgNO_3$ electrode was used as the reference electrode and was linked to the solution by a porous glass diaphragm. The solutions were kept under thermally controlled conditions at 15±0.5 °C and deaerated by bubbling argon. Electrochemical experiments were performed under an argon atmosphere. The working electrode was polished with figure-eight motions on a synthetic chamois leather pad using a Cr_2O_3-based polishing paste (~5 μm particle size) down to the mirror-like surface, and rinsed with acetonitrile. Polishing was carried before each recording of CV curve.

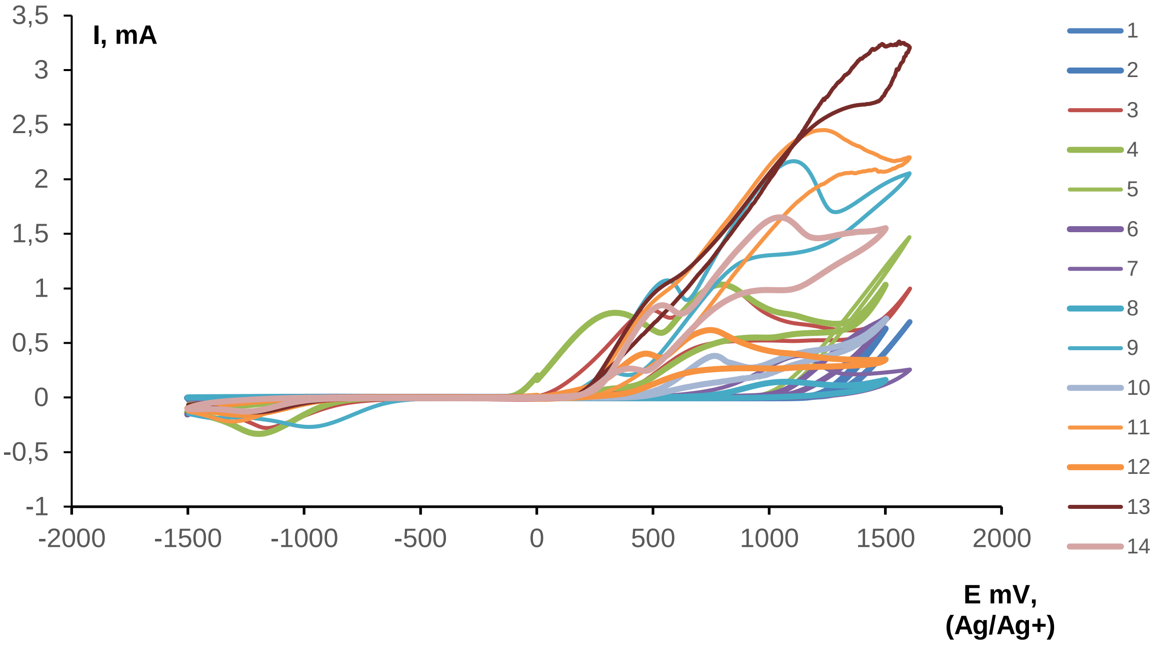

Figure S1. CV curves for the corresponding solutions on a working glassy-carbon electrode (d=3 mm) under a scan rate of 0.1 V/s. 1) 0.1 M *n*-Bu_4NBF_4 solution in DMF; 2) 0.2 M solution of *p*-TsOH·H_2O in 0.1 M *n*-Bu_4NBF_4 solution in DMF; 3) 0.1 M solution of KI in 0.1 M *n*-Bu_4NBF_4 solution in DMF; 4) 0.1 M solution of KI in 0.2 M solution of *p*-TsOH·H_2O in 0.1 M *n*-Bu_4NBF_4 solution in DMF; 5) 0.1 M solution of vinyl azide **1a** in 0.1 M *n*-Bu_4NBF_4 solution in DMF; 6) mixture of vinyl azide **1a** and *p*-TsOH·H_2O in 0.1 M *n*-Bu_4NBF_4 solution in DMF; 7) 0.2 M solution of amine **2a** in 0.1 M *n*-Bu_4NBF_4 solution in DMF; 8) mixture of amine **2a** and *p*-TsOH·H_2O in 0.1 M *n*-Bu_4NBF_4 solution in DMF; 9) 0.1 M solution of of vinyl azide **1a** in 0.1 M solution of KI in 0.1 M *n*-Bu_4NBF_4 solution in DMF; 10) mixture of vinyl azide **1a** and *p*-TsOH·H_2O in 0.1 M solution of KI in 0.1 M *n*-Bu_4NBF_4 solution in DMF; 11) 0.2 M solution of amine **2a** in 0.1 M *n*-Bu_4NBF_4 solution in DMF; 12) mixture of amine **2a** and *p*-TsOH·H_2O in 0.1 M solution of KI in 0.1 M *n*-Bu_4NBF_4 solution in DMF; 13) miture of vinyl azide **1a** and amine **2a** in 0.1 M solution of KI in 0.1 M *n*-Bu_4NBF_4 solution in DMF; 14) mixture of vinyl azide **1a,** amine **2a** and *p*-TsOH·H_2O in 0.1 M solution of KI in 0.1 M *n*-Bu_4NBF_4 solution in DMF.

Experimental Procedures for Scheme 4

a)

The solution of (1-azidovinyl)benzene **1a** (1.0 mmol, 145.2 mg, 1.0 eq.), benzylamine **2a** (2.0 mmol, 214.3 mg, 2.0 eq.), *p*-TsOH·H_2O (2.0 mmol, 380.0 mg, 2.0 eq.), and I_2 (4.0 mmol, 1.0 g, 4.0 eq.) in 10 mL of DMF was stirred at 70 °C for 160 min. After that the reaction mixture was diluted with H_2O (30 ml) and washed with mixture of PE and ethyl acetate (1:1) (2×30 mL). Combined organic layer was washed with 0.3 M solution of $Na_2S_2O_3$ (2×10 mL), water (2×10 mL), dried over Na_2SO_4 and concentrated under reduced pressure using a rotary evaporator (15-20 mmHg), (bath temperature, ca. 30–40 °C). Product **3a** was not detected. Product **5** was isolated by chromatography on SiO_2 (PE:EtOAc = from 15:1 to 2:1) The isolated yield is 20% (24.3 mg, 0.20 mmol).

b)

An undivided cell was equipped with a glassy carbon anode (3 cm^2) and a platinum plate cathode (3 cm^2) and connected to a DC regulated power supply. The solution of ω-iodoacetophenone **4** (1.0 mmol, 246.1 mg, 1.0 eq.), benzylamine **2a** (2.0 mmol, 214.3 mg, 2.0 eq.), *p*-TsOH·H_2O (2.0 mmol, 380.0 mg, 2.0 eq.), and KI (1.0 mmol, 170.0 mg, 1.0 eq.) in 10 mL of DMF was electrolyzed using constant current conditions at 70 °C under magnetic stirring for 160 min with I = 60 mA (j = 20 mA/cm^2). After that the reaction mixture was diluted with H_2O (30 ml) and washed with mixture of PE and ethyl acetate (1:1) (2×30 mL). Combined organic layer was washed with 0.3 M solution of $Na_2S_2O_3$ (2×10 mL), water (2×10 mL), dried over Na_2SO_4 and concentrated under reduced pressure using a rotary evaporator (15-20 mmHg), (bath temperature, ca. 30–40 °C). Product **3a** was not detected. Product **5** was isolated by chromatography on SiO_2 (PE:EtOAc = from 15:1 to 2:1) The isolated yield is 13% (15.6 mg, 0.20 mmol).

c)

An undivided cell was equipped with a glassy carbon anode (3 cm^2) and a platinum plate cathode (3 cm^2) and connected to a DC regulated power supply. The solution of acetophenone **5** (1.0 mmol, 120.2 mg, 1.0 eq.), benzylamine **2a** (2.0 mmol, 214.3 mg, 2.0 eq.), *p*-TsOH·H_2O (2.0 mmol, 380.0 mg, 2.0 eq.), and KI (1.0 mmol, 170.0 mg, 1.0 eq.) in 10 mL of DMF was electrolyzed using constant current conditions at 70 °C under magnetic stirring for 160 min with I = 60 mA (j = 20 mA/cm^2). After that the reaction mixture was diluted with H_2O (30 ml) and washed with mixture of PE and ethyl acetate (1:1) (2×30 mL). Combined organic layer was washed with 0.3 M solution of $Na_2S_2O_3$ (2×10 mL), water (2×10 mL), dried over Na_2SO_4 and concentrated under reduced pressure using a rotary evaporator (15-20 mmHg), (bath temperature, ca. 30–40 °C). Product **3a** was isolated by chromatography on SiO_2 (PE:EtOAc = from 15:1 to 2:1).

d)

An undivided cell was equipped with a glassy carbon anode (3 cm^2) and a platinum plate cathode (3 cm^2) and connected to a DC regulated power supply. The solution of 3-phenyl-2*H*-azirine **6** (1.0 mmol, 117.2 mg, 1.0 eq.), benzylamine **2a** (2.0 mmol, 214.3 mg, 2.0 eq.), *p*-TsOH·H_2O (2.0 mmol, 380.0 mg, 2.0 eq.), and KI (1.0 mmol, 170.0 mg, 1.0 eq.) in 10 mL of DMF was electrolyzed using constant current conditions at 70 °C under magnetic stirring for 160 min with I = 60 mA (j = 20 mA/cm^2). After that the reaction mixture was diluted with H_2O (30 ml) and washed with mixture of PE and ethyl acetate (1:1) (2×30 mL). Combined organic layer was washed with 0.3 M solution of $Na_2S_2O_3$ (2×10 mL), water (2×10 mL), dried over Na_2SO_4 and concentrated under reduced pressure using a rotary evaporator (15-20 mmHg), (bath temperature, ca. 30–40 °C). Product **3a** was isolated by chromatography on SiO_2 (PE:EtOAc = from 15:1 to 2:1).

Experimental Procedures for Scheme 5.

a)

A divided cell was equipped with a glassy carbon anode (3 cm^2) and a platinum plate cathode (3 cm^2) and connected to a DC regulated power supply. Anodic space: the solution of (1-azidovinyl)benzene **1a**

(1.0 mmol, 145.2 mg, 1.0 eq.), benzylamine **2a** (2.0 mmol, 214.3 mg, 2.0 eq.), KI (1.0 mmol, 166.0 mg, 1.0 eq.) and supporting electrolyte n-Bu_4NBF_4 (0.5 mmol, 164.6 mg) in 10 mL of DMF. Cathodic space: KI (1.0 mmol, 166.0 mg, 1.0 eq) and supporting electrolyte *n*-Bu_4NBF_4 (0.5 mmol, 164.6 mg) in 10 mL of DMF. The solutions were electrolyzed using constant current conditions at 25 °C under magnetic stirring for 320 min with I = 20 mA. After that the reaction mixture was diluted with H_2O (30 ml) and washed with mixture of PE and ethyl acetate (1:1) (2×30 mL). Combined organic layer was washed with 0.3 M solution of $Na_2S_2O_3$ (2×10 mL), water (2×10 mL), dried over Na_2SO_4 and concentrated under reduced pressure using a rotary evaporator (15-20 mmHg), (bath temperature, ca. 30–40 °C). Product **3a** was isolated by chromatography on SiO_2 (PE:EtOAc = from 15:1 to 2:1).

b)

A divided cell was equipped with a glassy carbon anode (3 cm^2) and a platinum plate cathode (3 cm^2) and connected to a DC regulated power supply. Anodic space: the solution of (1-azidovinyl)benzene **1a** (1.0 mmol, 145.2 mg, 1.0 eq.), benzylamine **2a** (2.0 mmol, 214.3 mg, 2.0 eq.), *p*-TsOH·H_2O (2.0 mmol, 380.0 mg, 2.0 eq.), KI (1.0 mmol, 166.0 mg, 1.0 eq.), and supporting electrolyte *n*-Bu_4NBF_4 (0.5 mmol, 164.6 mg) in 10 mL of DMF. Cathodic space: KI (1.0 mmol, 166.0 mg, 1.0 eq) and supporting electrolyte *n*-Bu_4NBF_4 (0.5 mmol, 164.6 mg) in 10 mL of DMF. The solutions were electrolyzed using constant current conditions at 25 °C under magnetic stirring for 320 min with I = 20 mA. After that the reaction mixture was diluted with H_2O (30 ml) and washed with mixture of PE and ethyl acetate (1:1) (2×30 mL). Combined organic layer was washed with 0.3 M solution of $Na_2S_2O_3$ (2×10 mL), water (2×10 mL), dried over Na_2SO_4 and concentrated under reduced pressure using a rotary evaporator (15-20 mmHg), (bath temperature, ca. 30–40 °C). Product **3a** was isolated by chromatography on SiO_2 (PE:EtOAc = from 15:1 to 2:1).

c)

An undivided cell was equipped with a glassy carbon anode (3 cm^2) and a platinum plate cathode (3 cm^2) and connected to a DC regulated power supply. The solution of (1-Azidovinyl)benzene **1a** (1.0 mmol, 145.2 mg, 1.0 eq.), benzylamine **2a** (2.0 mmol, 214.3 mg, 2.0 eq.), and KI (1.0 mmol, 166.0 mg, 1.0 eq.) in 10 mL of DMF was electrolyzed using constant current conditions at 25 °C under magnetic stirring for 320 min with I = 20 mA. After that the reaction mixture was diluted with H_2O (30 ml) and washed with mixture of PE and ethyl acetate (1:1) (2×30 mL). Combined organic layer was washed with 0.3 M solution of $Na_2S_2O_3$ (2×10 mL), water (2×10 mL), dried over Na_2SO_4 and concentrated under reduced pressure using a rotary evaporator (15-20 mmHg), (bath temperature, ca. 30–40 °C). Product **3a** was isolated by chromatography on SiO_2 (PE:EtOAc = from 15:1 to 2:1).

d)

An undivided cell was equipped with a glassy carbon anode (3 cm^2) and a platinum plate cathode (3 cm^2) and connected to a DC regulated power supply. The solution of (1-azidovinyl)benzene **1a** (1.0 mmol, 145.2 mg, 1.0 eq.), benzylamine **2a** (2.0 mmol, 214.3 mg, 2.0 eq.), *p*-TsOH·H_2O (2.0 mmol, 380.0 mg, 2.0 eq.), and KI (1.0 mmol, 166.0 mg, 1.0 eq.) in 10 mL of DMF was electrolyzed using constant current conditions at 25 °C under magnetic stirring for 320 min with I = 20 mA. After that the reaction mixture was diluted with H_2O (30 ml) and washed with mixture of PE and ethyl acetate (1:1) (2×30 mL). Combined organic layer was washed with 0.3 M solution of $Na_2S_2O_3$ (2×10 mL), water (2×10 mL), dried over Na_2SO_4 and concentrated under reduced pressure using a rotary evaporator (15-20 mmHg), (bath temperature, ca. 30–40 °C). Product **3a** was isolated by chromatography on SiO_2 (PE:EtOAc = from 15:1 to 2:1).

References

1. R. Dey, P. Banerjee, *Org. Lett.* **2017**, 19, 304-307.
2. M. Andresini, L. Degannaro, R. Luisi Beilstein *J. Org. Chem.* **2021**, 17, 203–209.
3. L. Xiang, Y. Niu, X. Pang, X. Yang, R. Yan. *Chem. Commun*. **2015**, 51, 6598-6600.
4. Z. Yang, J. Zhang, L. Hu, A. Li, L. Li, K. Liu, T. Yang, C. Zhou. *J. Org. Chem.* **2020**, 85, 5952−5958.

NMR spectra of synthesized compounds

^{1}H NMR of 3a

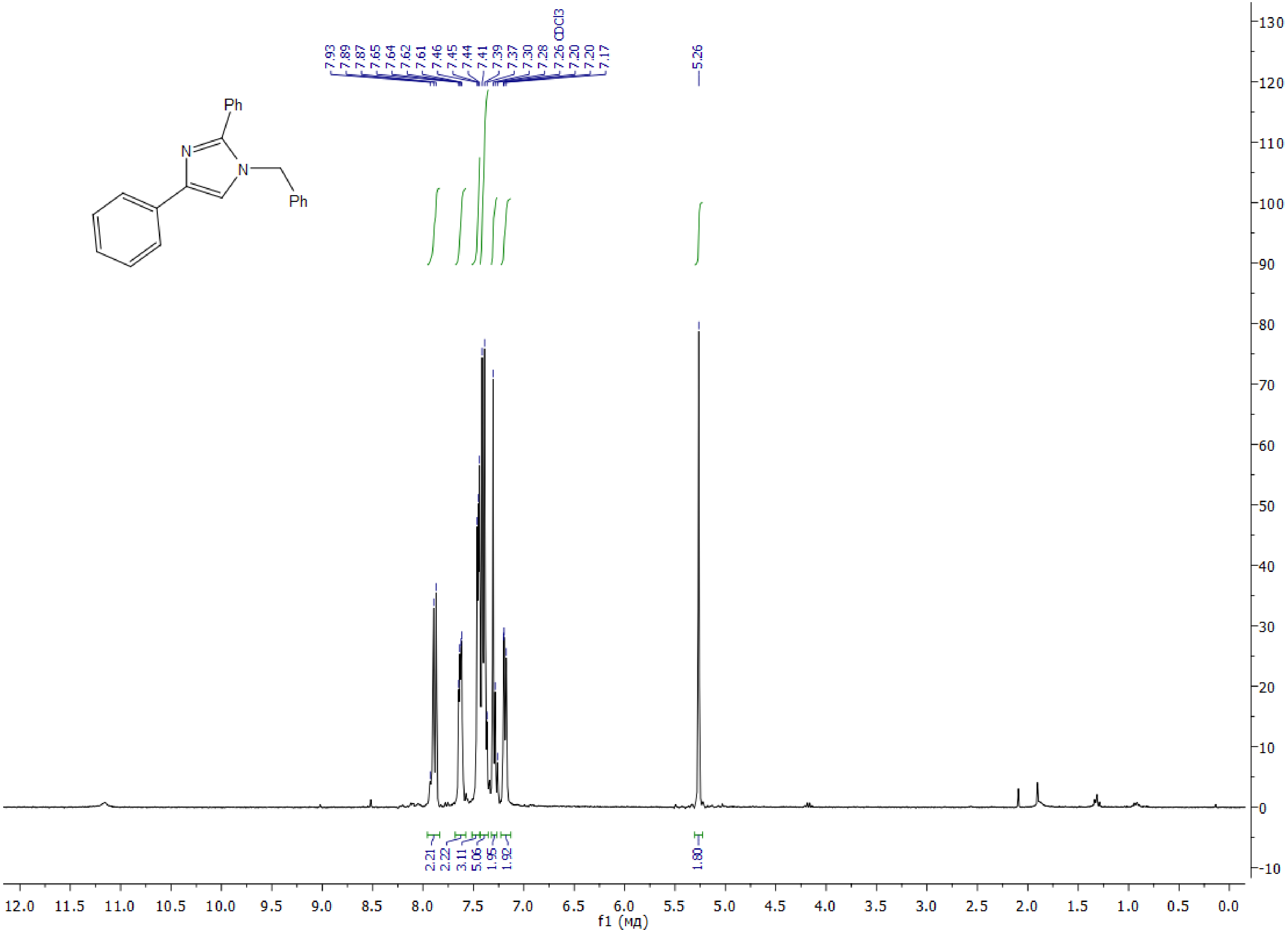

^{13}C NMR of 3a

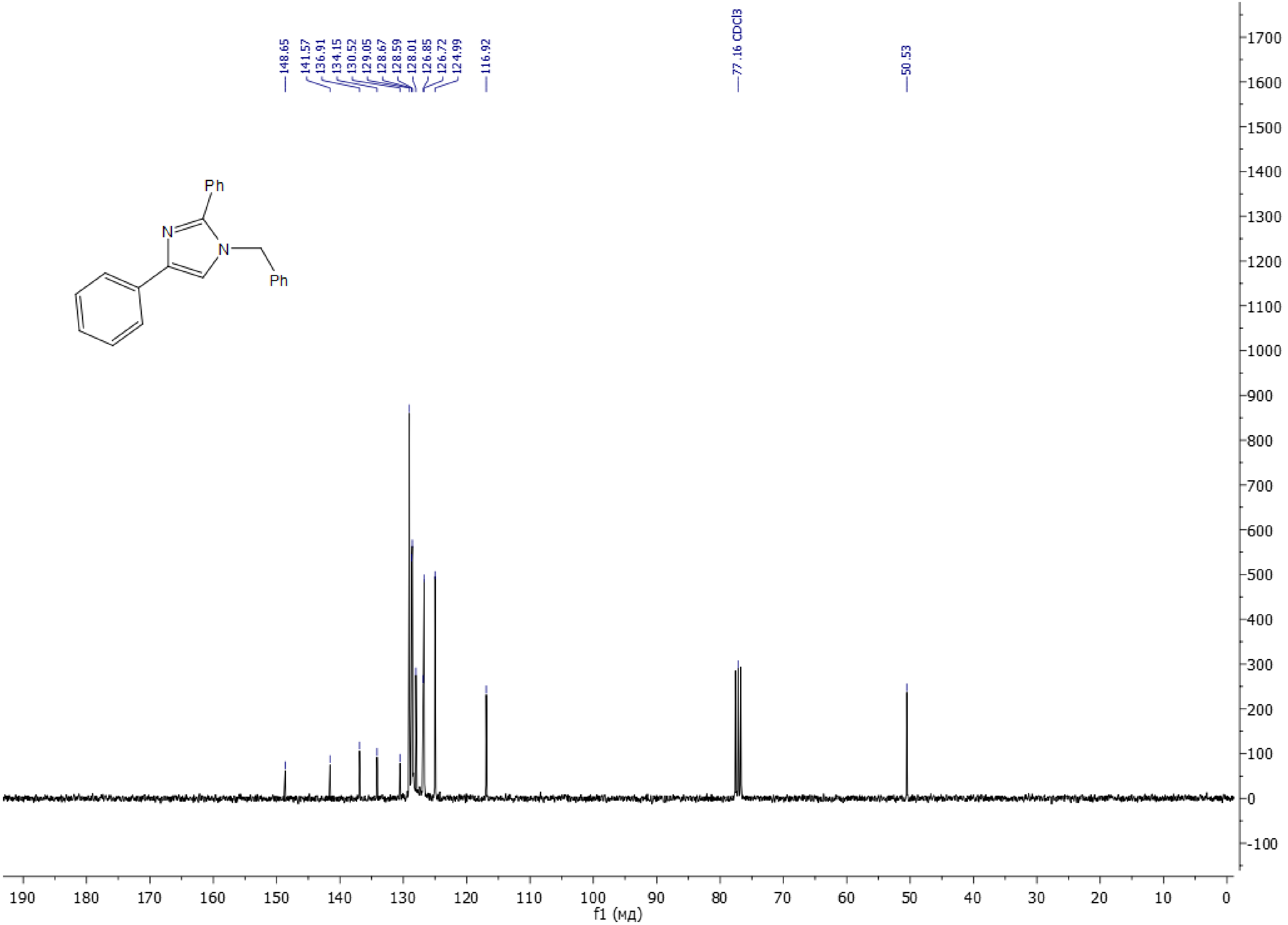

a

^{1}H NMR of 3b

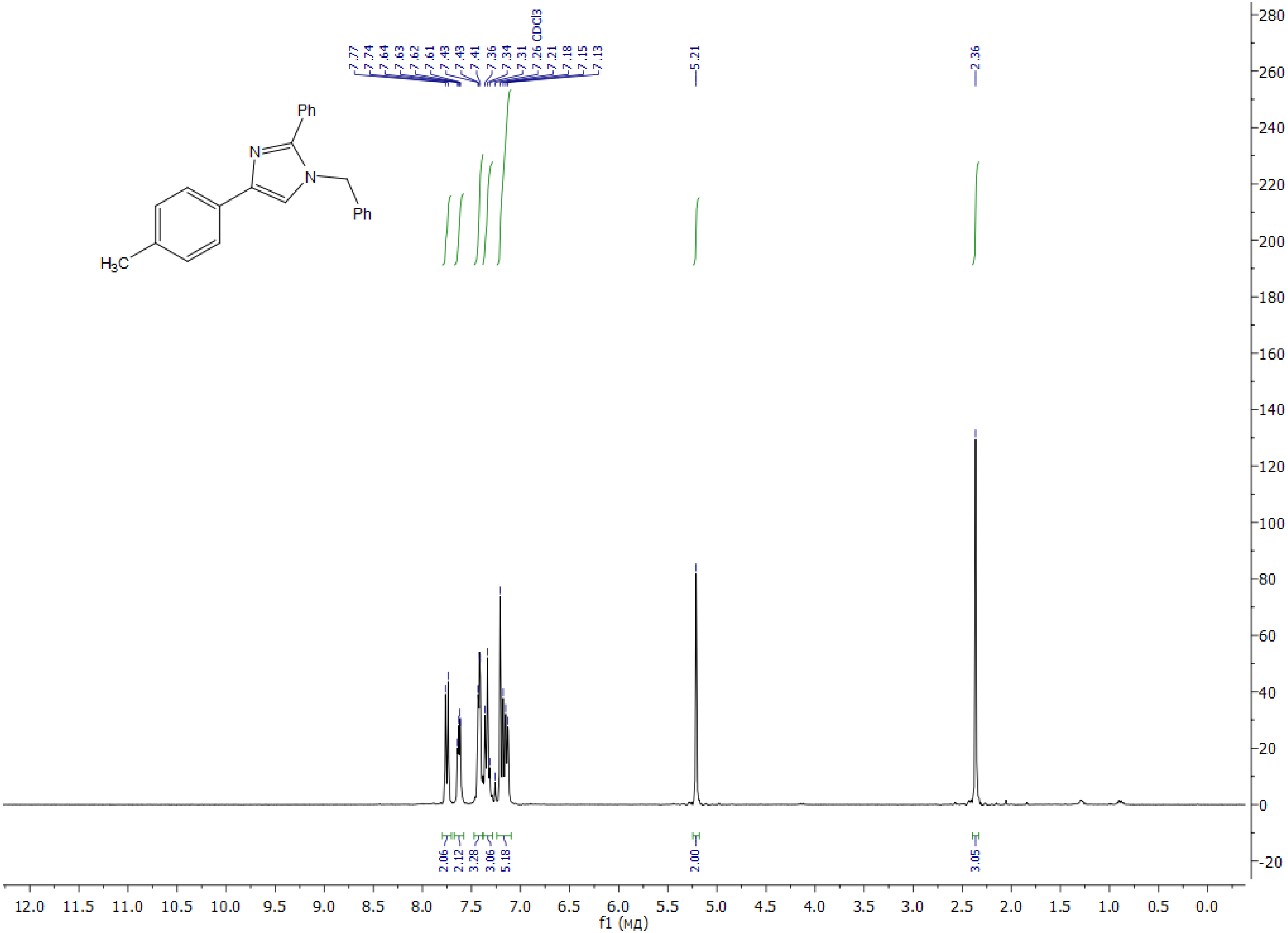

^{13}C NMR of 3b

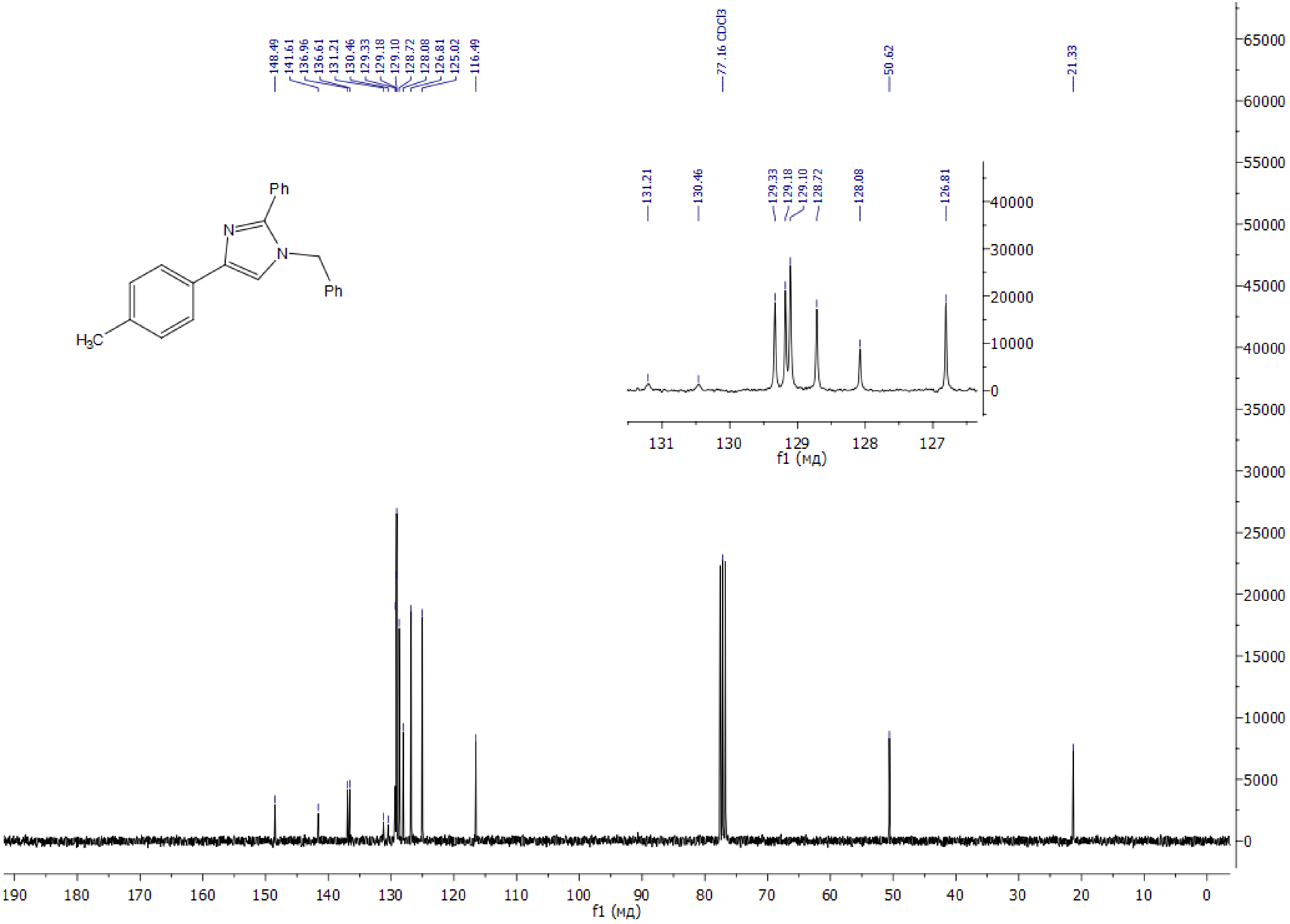
148.49
141.61
136.96
136.61
131.21
130.46
129.33
129.18
129.10
128.72
128.08
126.81
125.02
116.49
77.16 CDCl3
50.62
21.33
Ph
N
N
Ph
H3C
f1 (мд)

^{1}H NMR of 3c

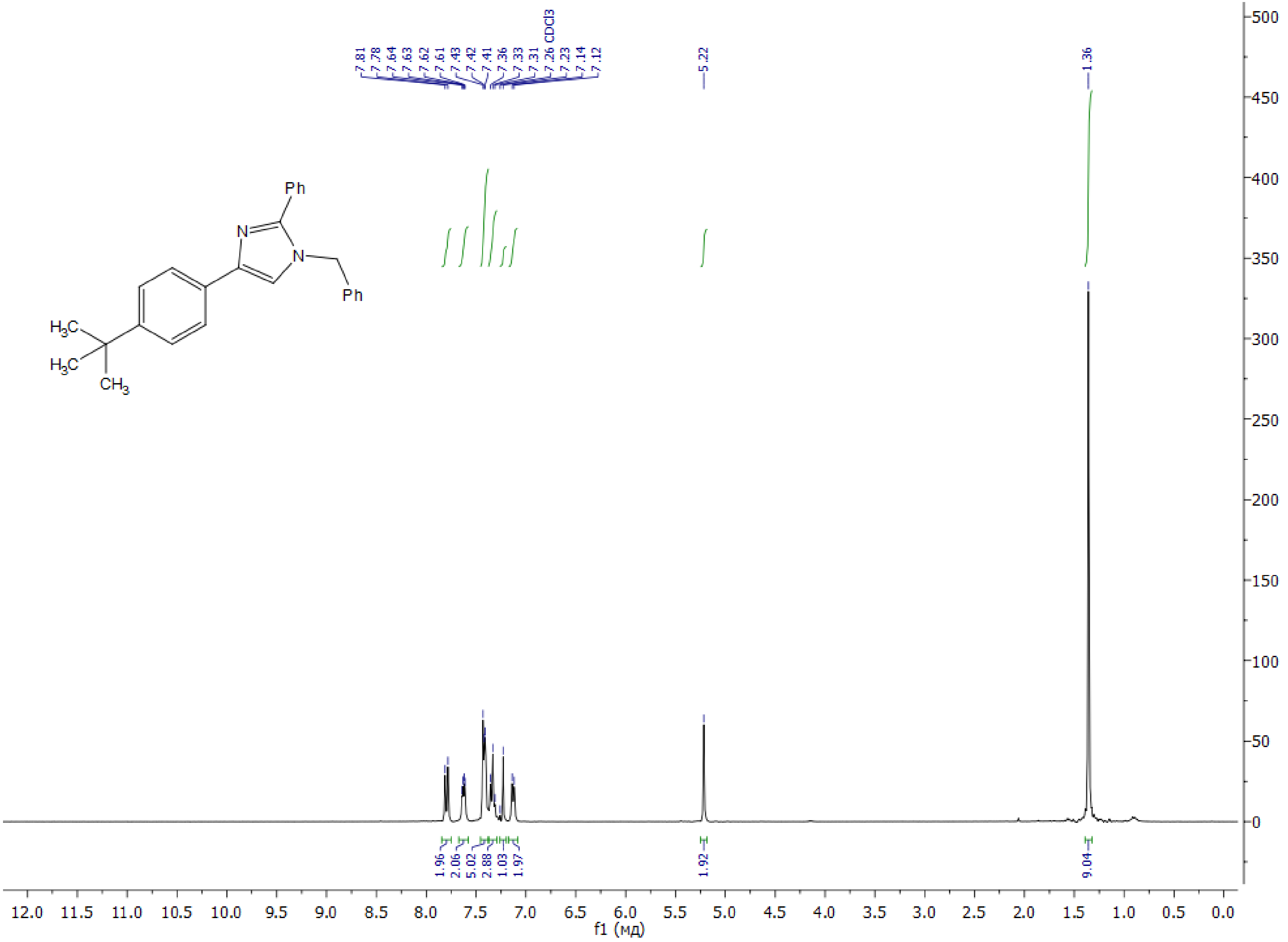
Ph
N
N
Ph
H3C
H3C
CH3
7.81
7.78
7.64
7.63
7.62
7.61
7.43
7.42
7.41
7.36
7.33
7.31
7.26 CDCl3
7.23
7.14
7.12
5.22
1.36
1.96
2.06
5.02
2.88
1.03
1.97
1.92
9.04
f1 (мд)

^{13}C NMR of 3c

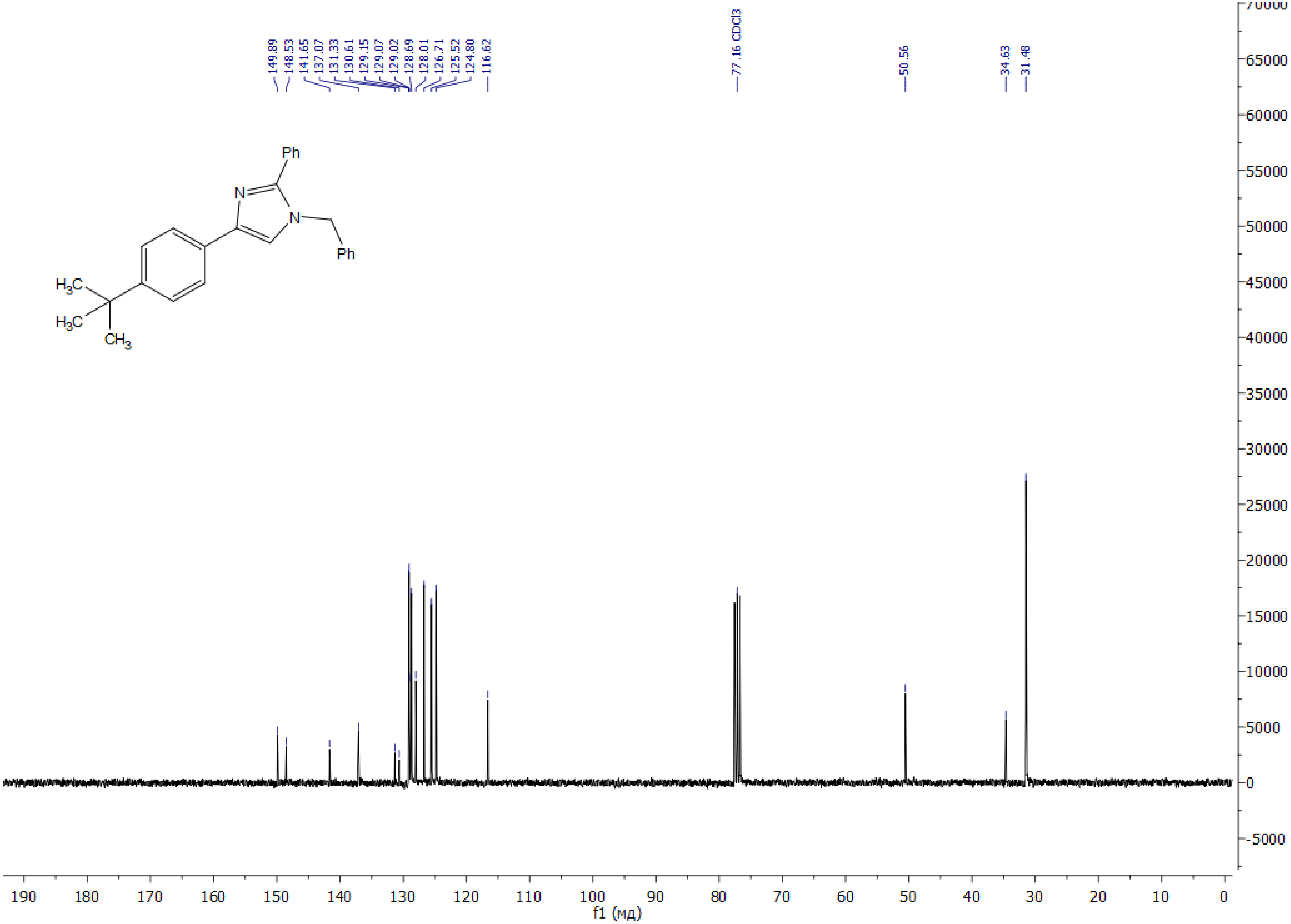
149.89
148.53
141.65
137.07
131.33
130.61
129.15
129.07
129.02
128.69
128.01
126.71
125.52
124.80
116.62
77.16 CDCl3
50.56
34.63
31.48
Ph
N
N
Ph
H3C
H3C
CH3
f1 (мд)

^{1}H NMR of 3d

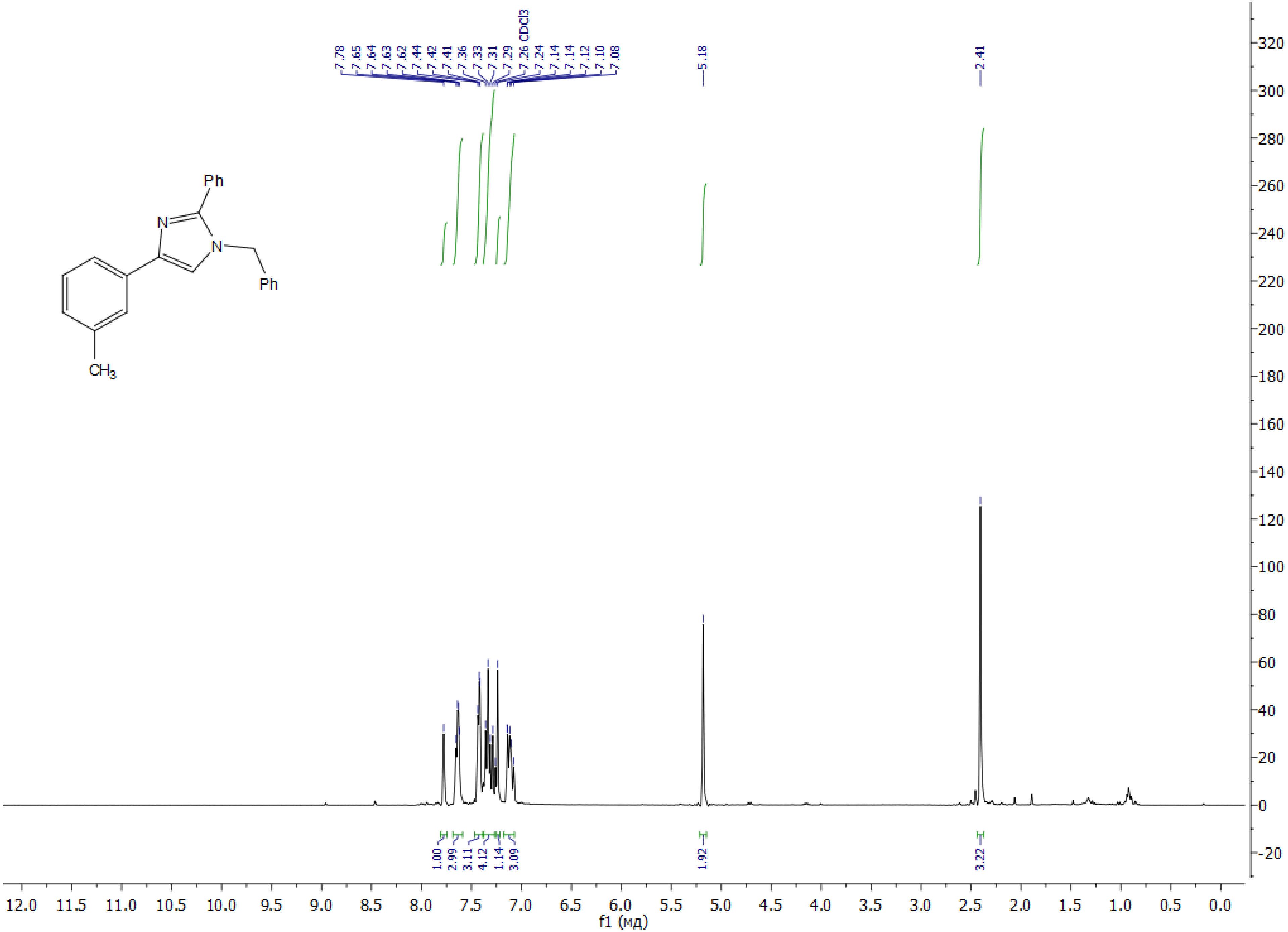

Ph
N
N
Ph
CH3
7.78
7.65
7.64
7.63
7.62
7.44
7.42
7.41
7.36
7.33
7.31
7.29
7.26 CDCl3
7.24
7.14
7.14
7.12
7.10
7.08
5.18
2.41
1.00
2.99
3.11
4.12
1.14
3.09
1.92
3.22
f1 (мд)

^{13}C NMR of 3d

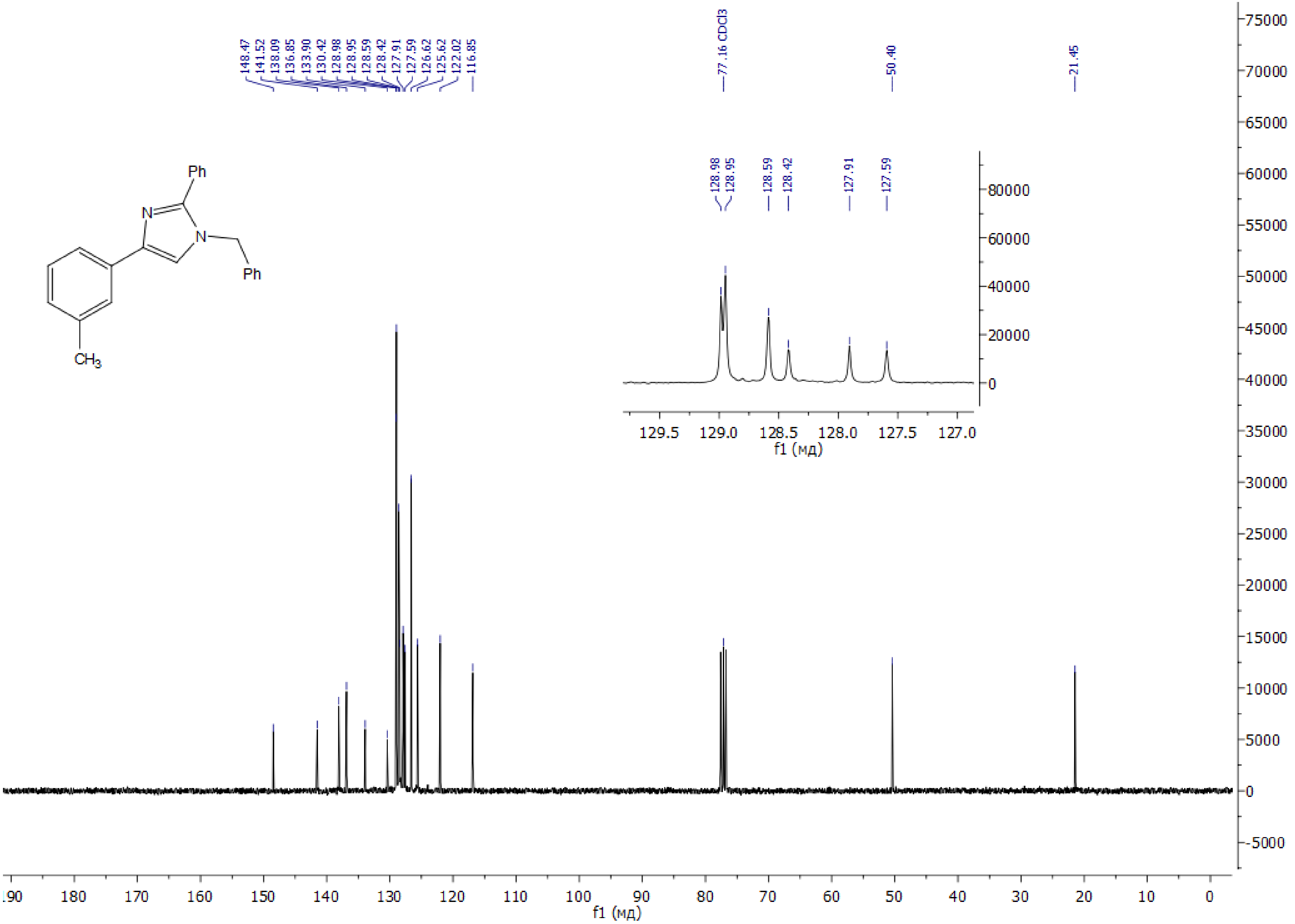

Ph
N
N
Ph
CH3
148.47
141.52
138.09
136.85
133.90
130.42
128.98
128.95
128.59
128.42
127.91
127.59
126.62
125.62
122.02
116.85
77.16 CDCl3
50.40
21.45
f1 (мд)

^{1}H NMR of 3e

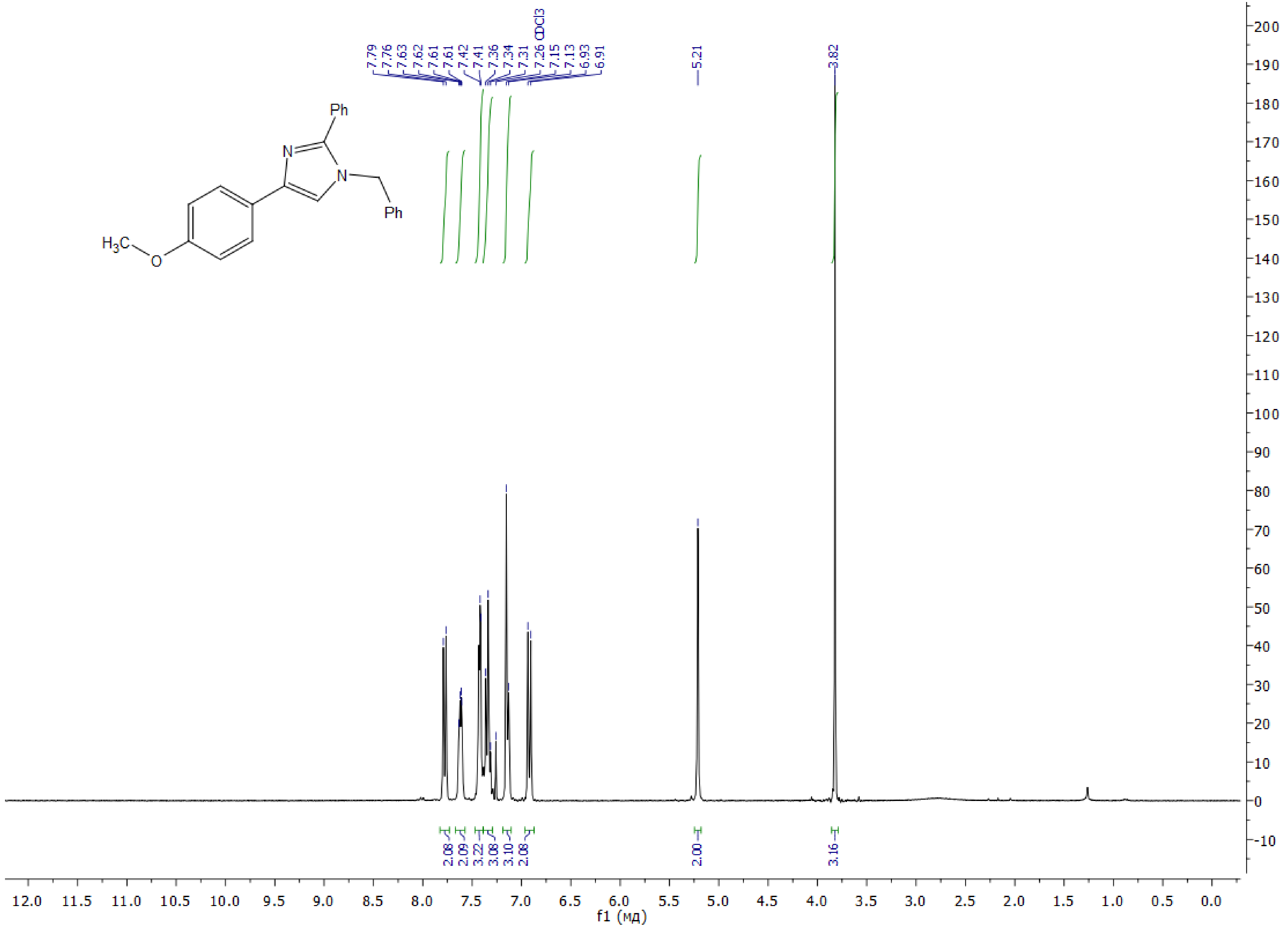

Ph
N
N
Ph
H3C
O
7.79
7.76
7.63
7.62
7.61
7.61
7.42
7.41
7.36
7.34
7.31
7.26 CDCl3
7.15
7.13
6.93
6.91
5.21
3.82
2.08
2.09
3.22
3.08
3.10
2.08
2.00
3.16
f1 (мд)

^{13}C NMR of 3e

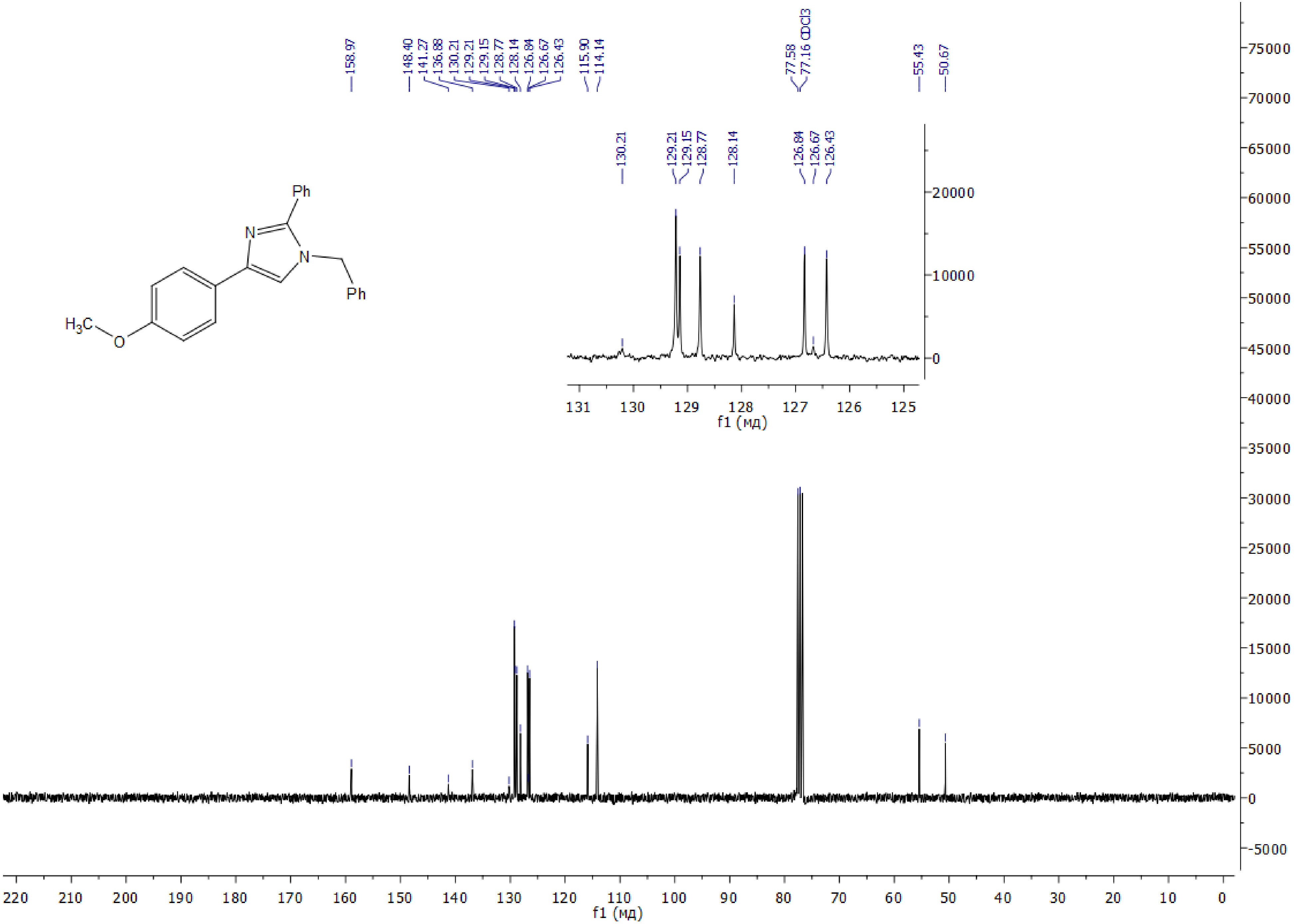

158.97
148.40
141.27
136.88
130.21
129.21
129.15
128.77
128.14
126.84
126.67
126.43
115.90
114.14
77.58
77.16 CDCl3
55.43
50.67
Ph
N
N
Ph
H3C
O
f1 (мд)

^{1}H NMR of 3f

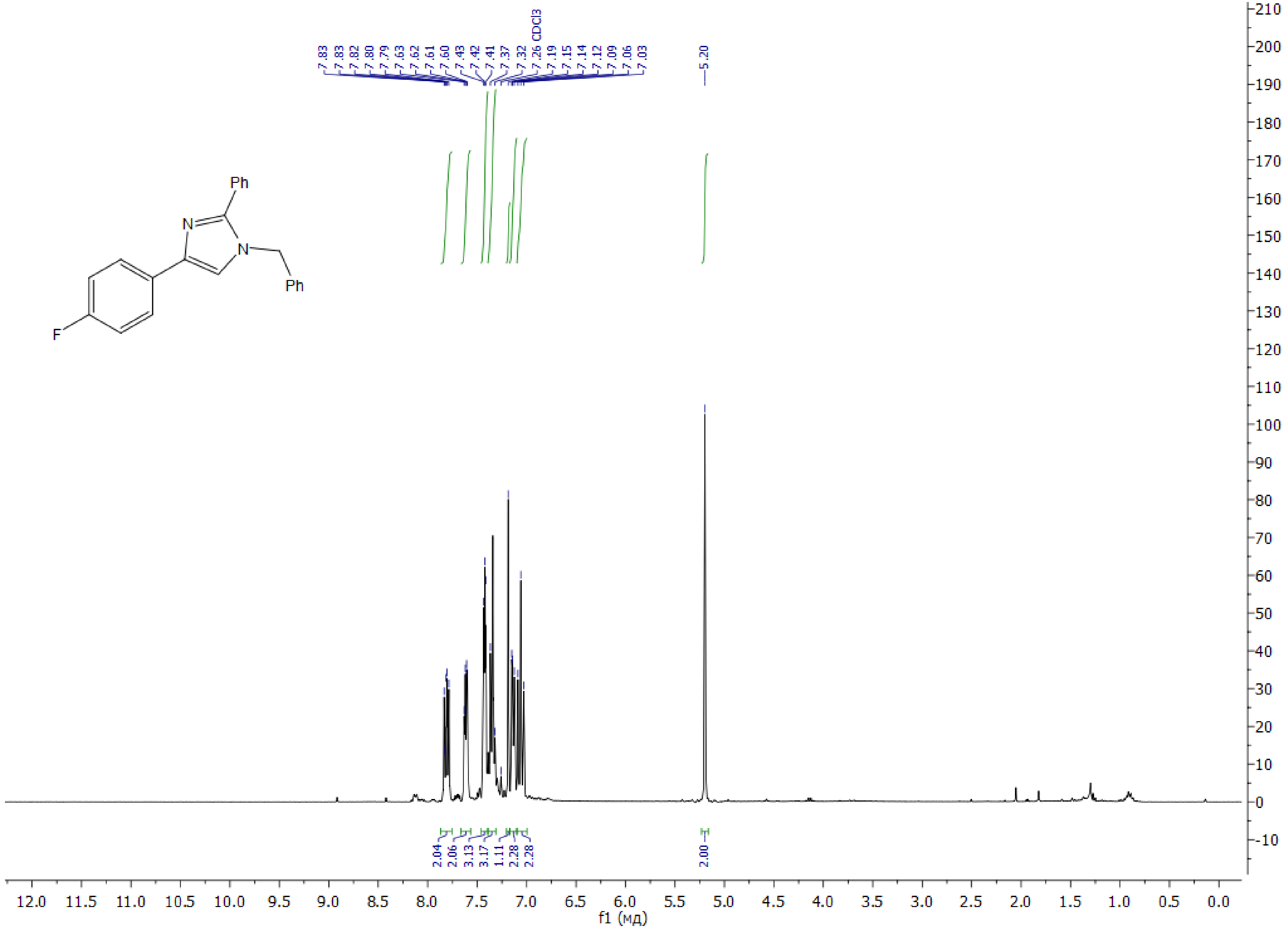

Ph
N
N
Ph
F
CDCl3
7.83
7.83
7.82
7.80
7.79
7.63
7.62
7.61
7.60
7.43
7.42
7.41
7.37
7.32
7.26
7.19
7.15
7.14
7.12
7.09
7.06
7.03
5.20
2.04
2.06
3.13
3.17
1.11
2.28
2.28
2.00
f1 (мд)

^{13}C NMR of 3f

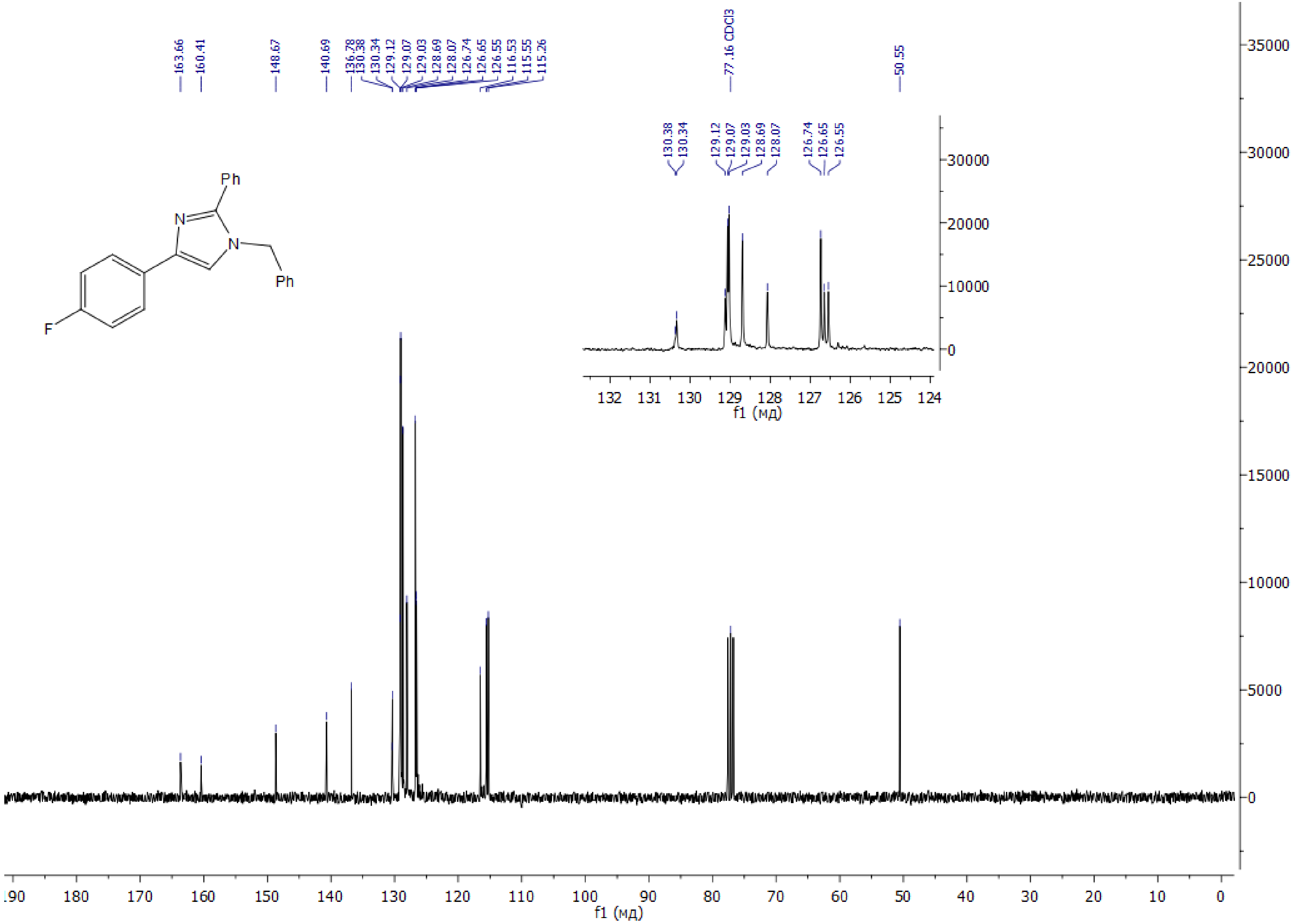
163.66
160.41
148.67
140.69
136.78
130.38
130.34
129.12
129.07
129.03
128.69
128.07
126.74
126.65
126.55
116.53
115.55
115.26
77.16 CDCl3
50.55
Ph
N
N
Ph
F
f1 (мд)
f1 (мд)

^{1}H NMR of 3g

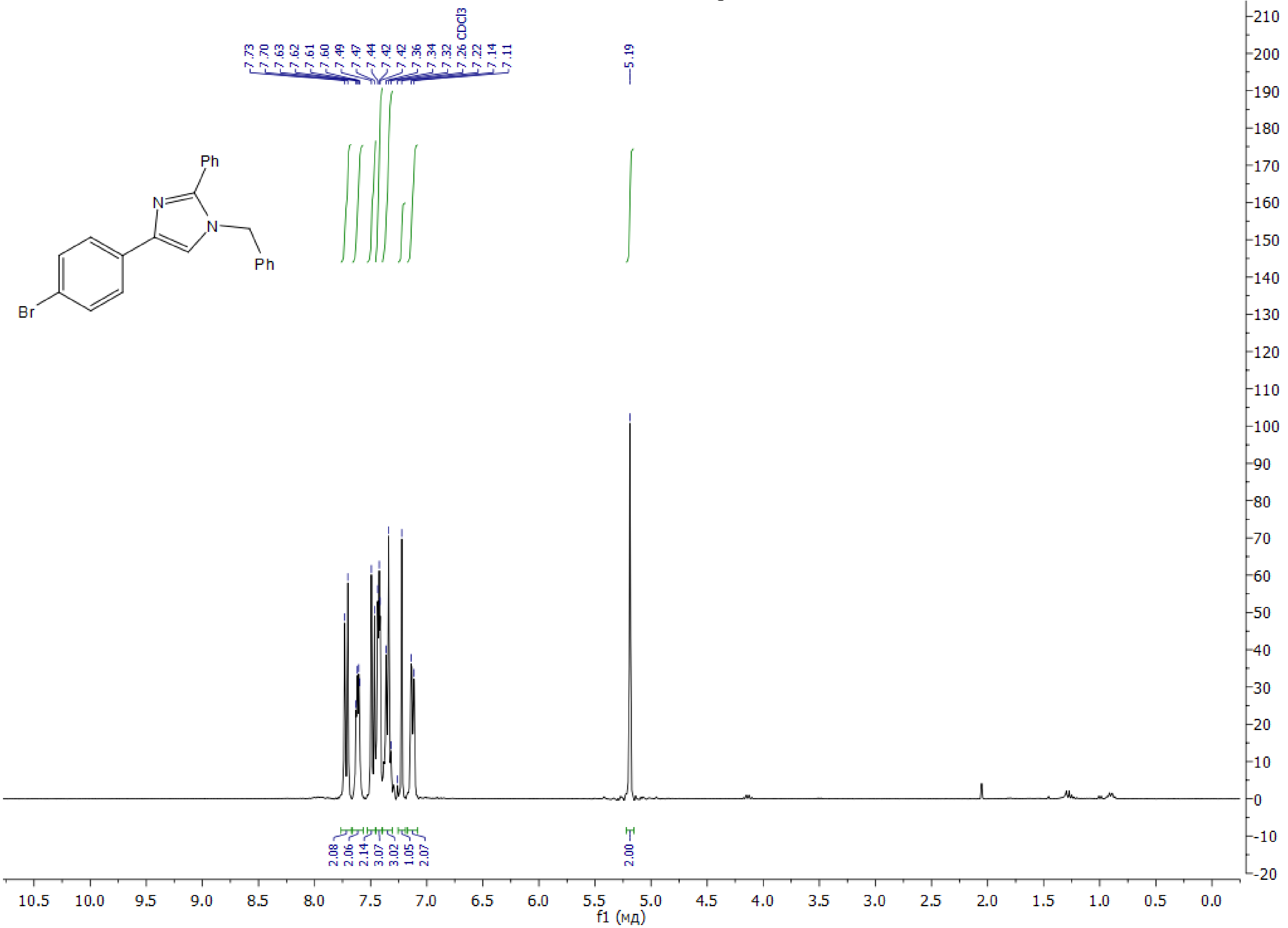

7.73
7.70
7.63
7.62
7.61
7.60
7.49
7.47
7.44
7.42
7.42
7.36
7.34
7.32
7.26 CDCl3
7.22
7.14
7.11
5.19
Ph
N
N
Ph
Br
2.08
2.06
2.14
3.07
3.02
1.05
2.07
2.00
f1 (мд)

^{13}C NMR of 3g

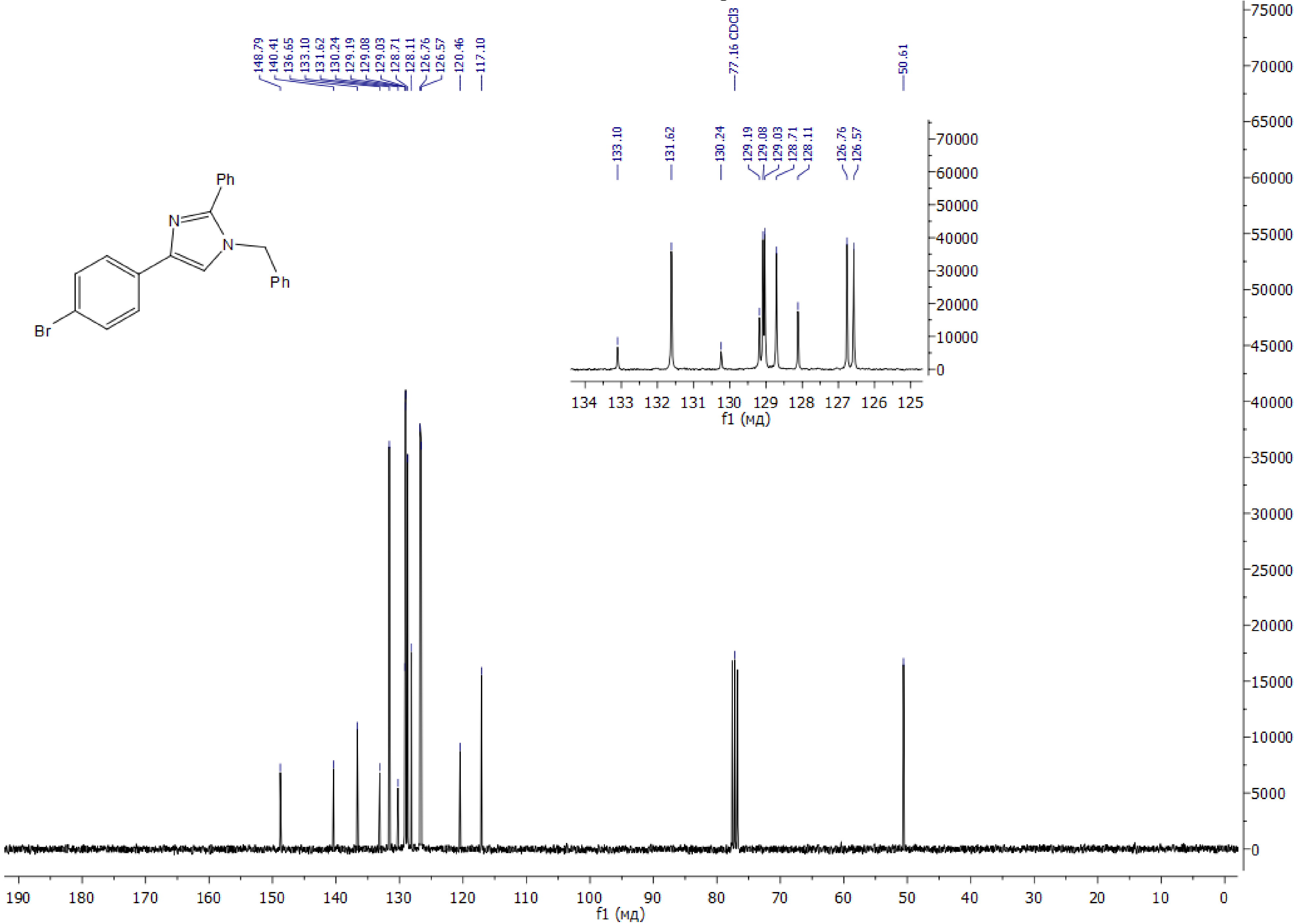
148.79
140.41
136.65
133.10
131.62
130.24
129.19
129.08
129.03
128.71
128.11
126.76
126.57
120.46
117.10
77.16 CDCl3
50.61
Ph
N
N
Ph
Br
f1 (мд)

^{1}H NMR of 3h

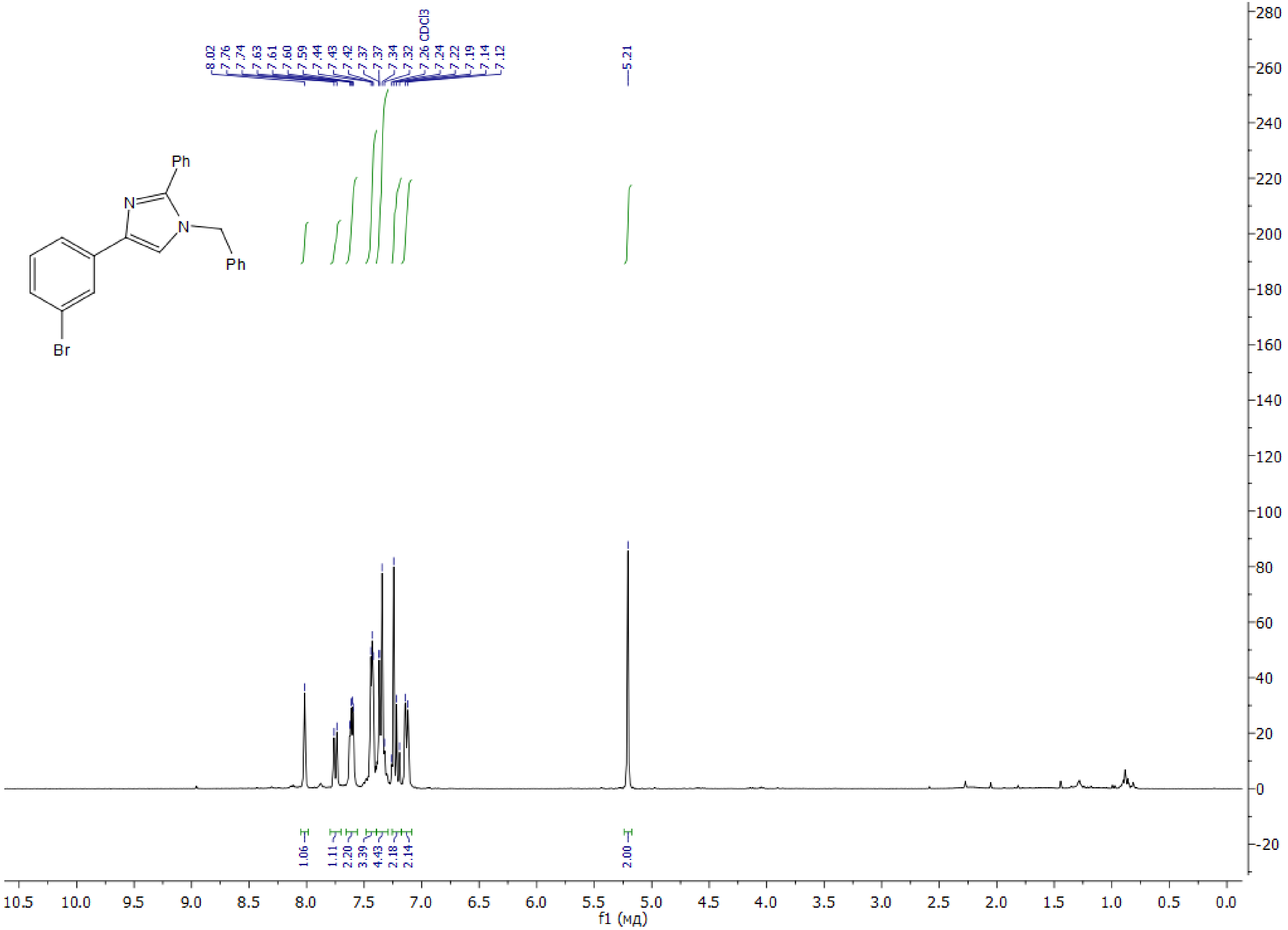
Ph
N
N
Ph
Br
8.02
7.76
7.74
7.63
7.61
7.60
7.59
7.44
7.43
7.42
7.37
7.37
7.34
7.32
7.26 CDCl3
7.24
7.22
7.19
7.14
7.12
5.21
1.06
1.11
2.20
3.39
4.43
2.18
2.14
2.00
f1 (мд)

^{13}C NMR of 3h

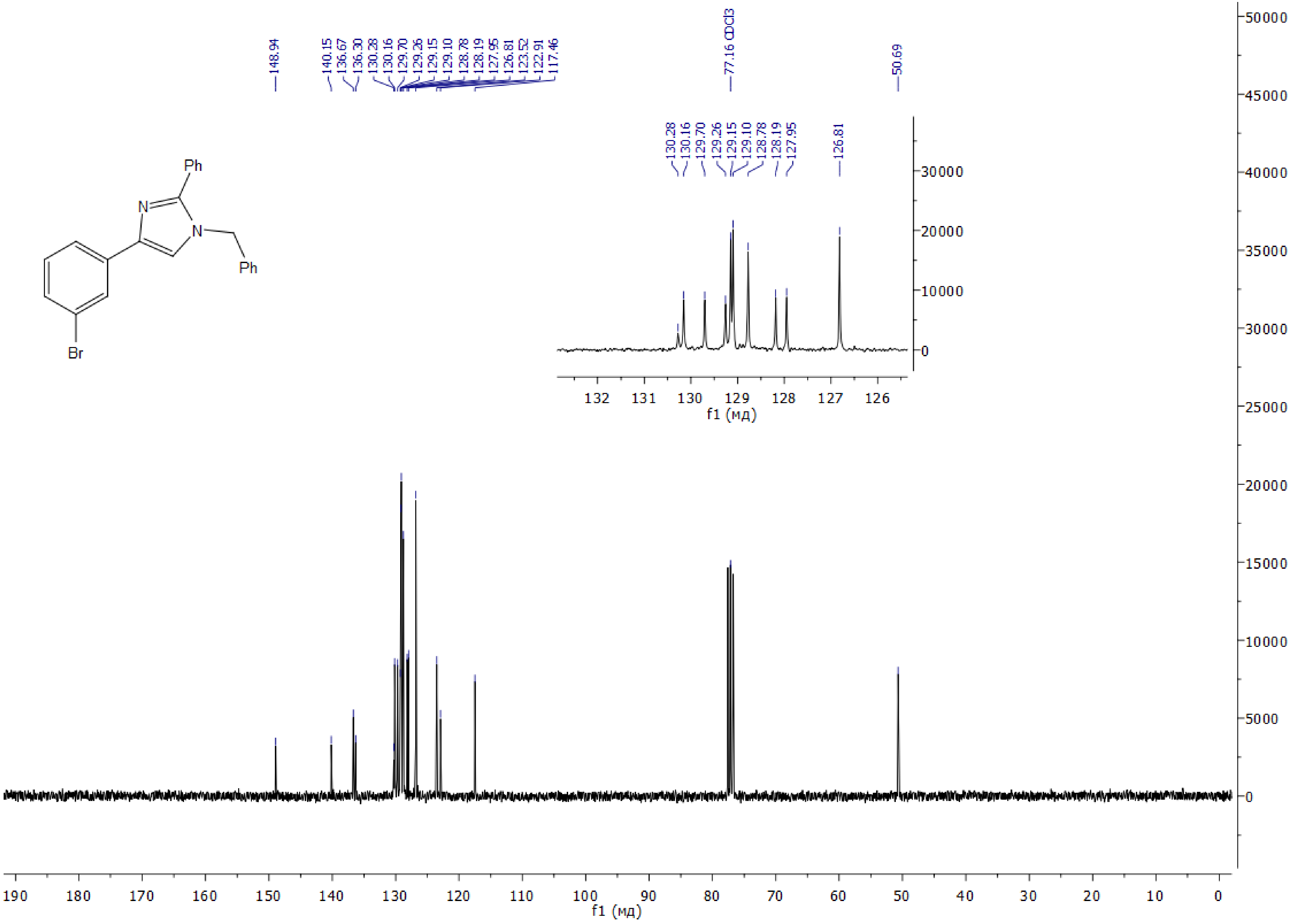

Ph
N
N
Ph
Br
148.94
140.15
136.67
136.30
130.28
130.16
129.70
129.26
129.15
129.10
128.78
128.19
127.95
126.81
123.52
122.91
117.46
77.16 CDCl3
50.69
f1 (мд)

^{1}H NMR of 3i

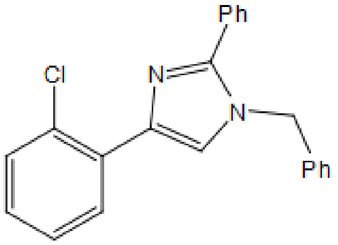

Cl
N
Ph
N
Ph

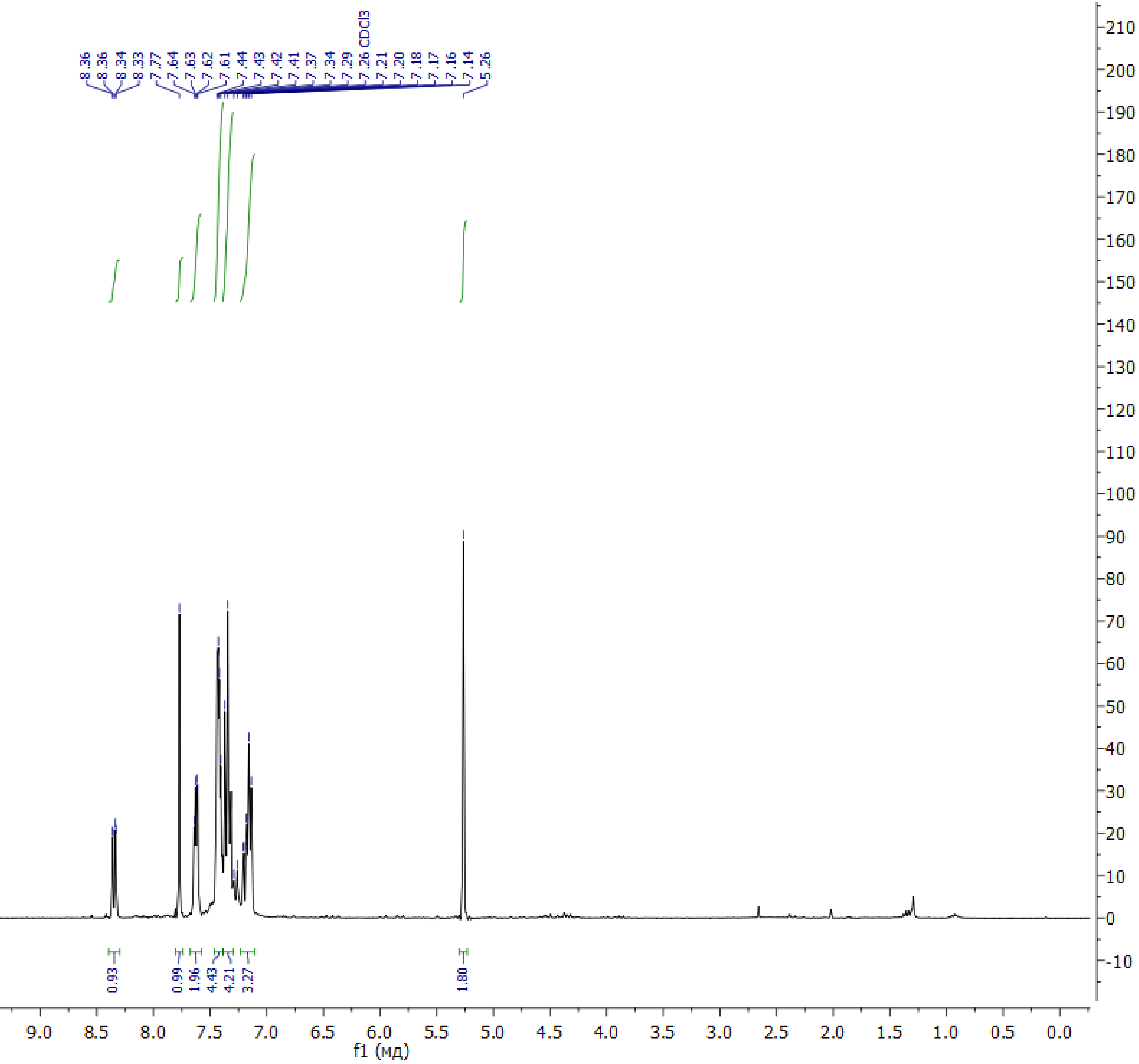

8.36
8.36
8.34
8.33
7.77
7.64
7.63
7.62
7.61
7.44
7.43
7.42
7.41
7.37
7.34
7.29
7.26 CDCl3
7.21
7.20
7.18
7.17
7.16
7.14
5.26
0.93
0.99
1.96
4.43
4.21
3.27
1.80
f1 (мд)

^{13}C NMR of 3i

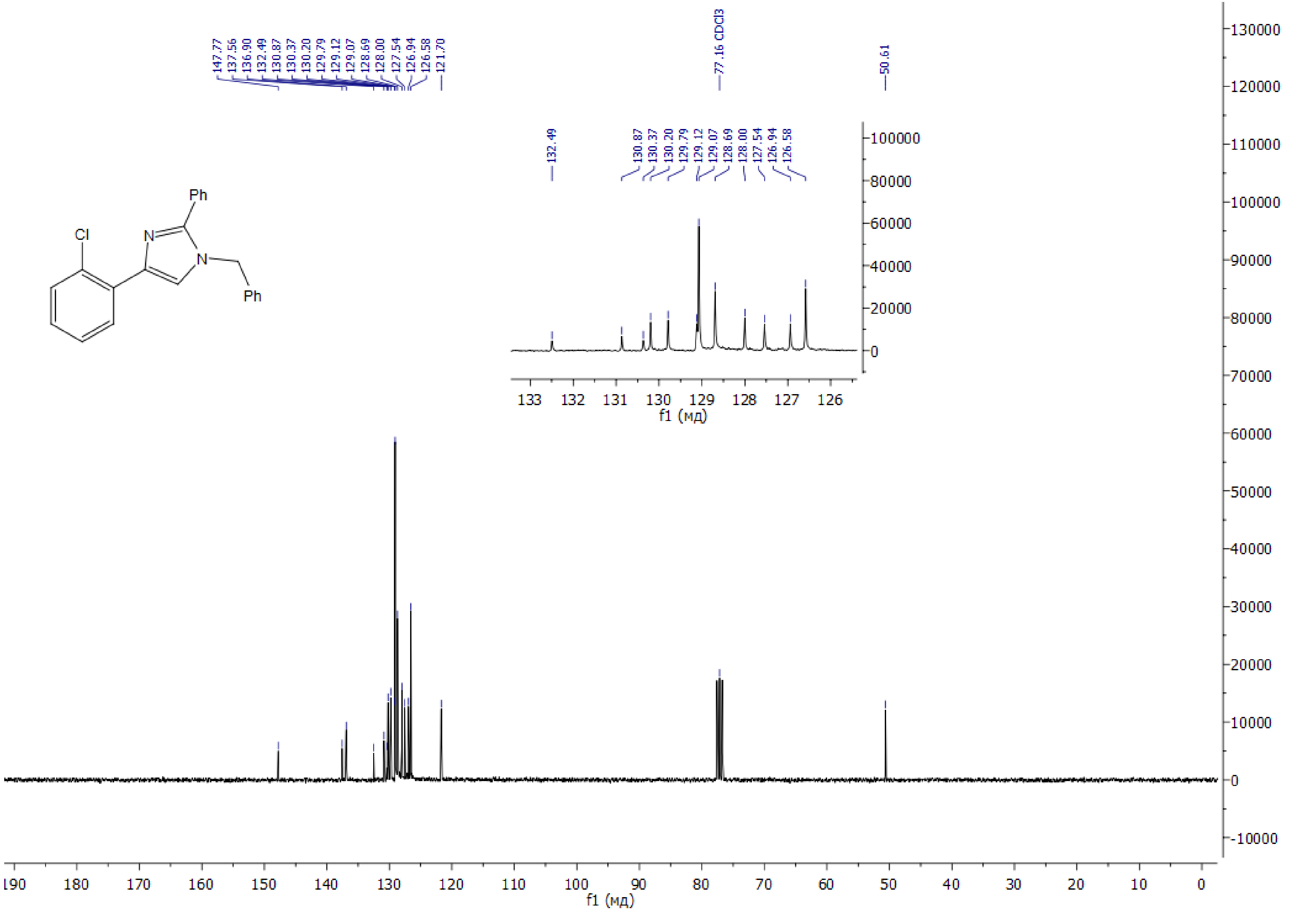

147.77
137.56
136.90
132.49
130.87
130.37
130.20
129.79
129.12
129.07
128.69
128.00
127.54
126.94
126.58
121.70
77.16 CDCl3
50.61
Cl
N
N
Ph
Ph
132.49
130.87
130.37
130.20
129.79
129.12
129.07
128.69
128.00
127.54
126.94
126.58
f1 (мд)
f1 (мд)

^{1}H NMR of 3j

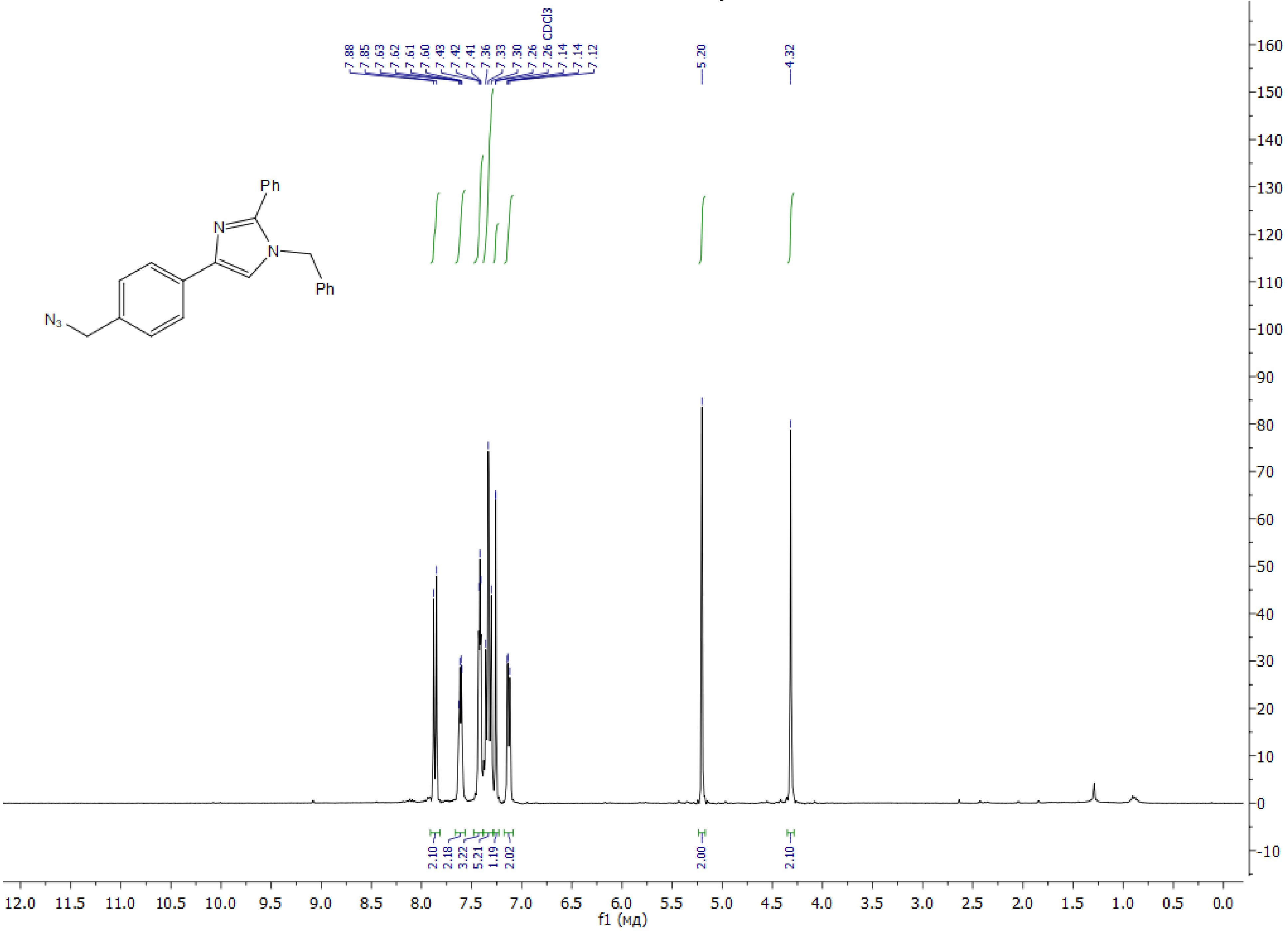

7.88
7.85
7.63
7.62
7.61
7.60
7.43
7.42
7.41
7.36
7.33
7.30
7.26
7.26 CDCl3
7.14
7.14
7.12
5.20
4.32
Ph
N
N
Ph
N3
2.10
2.18
3.22
5.21
1.19
2.02
2.00
2.10
f1 (мд)

^{13}C NMR of 3j

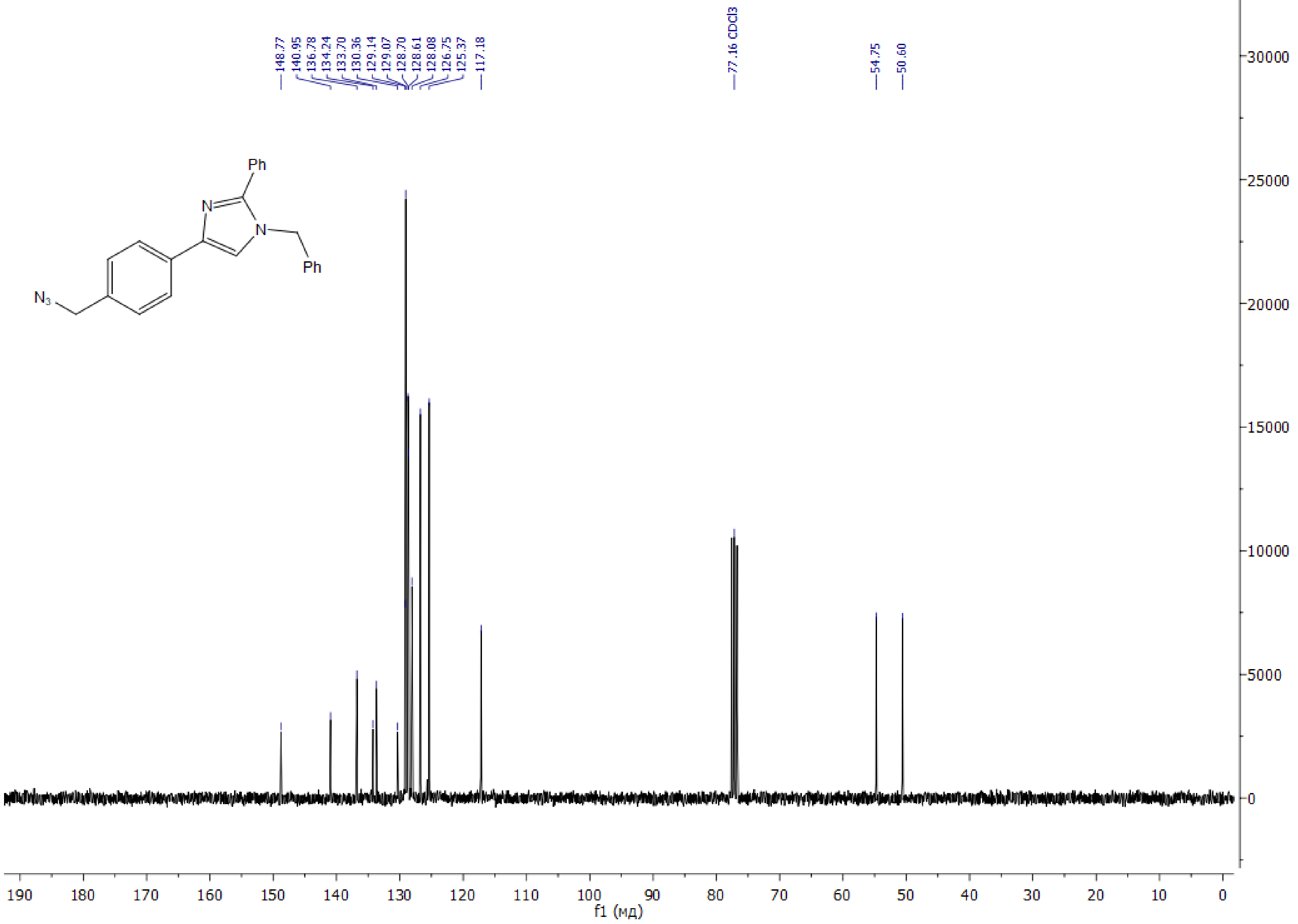
148.77
140.95
136.78
134.24
133.70
130.36
129.14
129.07
128.70
128.61
128.08
126.75
125.37
117.18
77.16 CDCl3
54.75
50.60
Ph
N
N
Ph
N3
30000
25000
20000
15000
10000
5000
0
190
180
170
160
150
140
130
120
110
100
90
80
70
60
50
40
30
20
10
0
f1 (мд)

^{1}H NMR of 3l

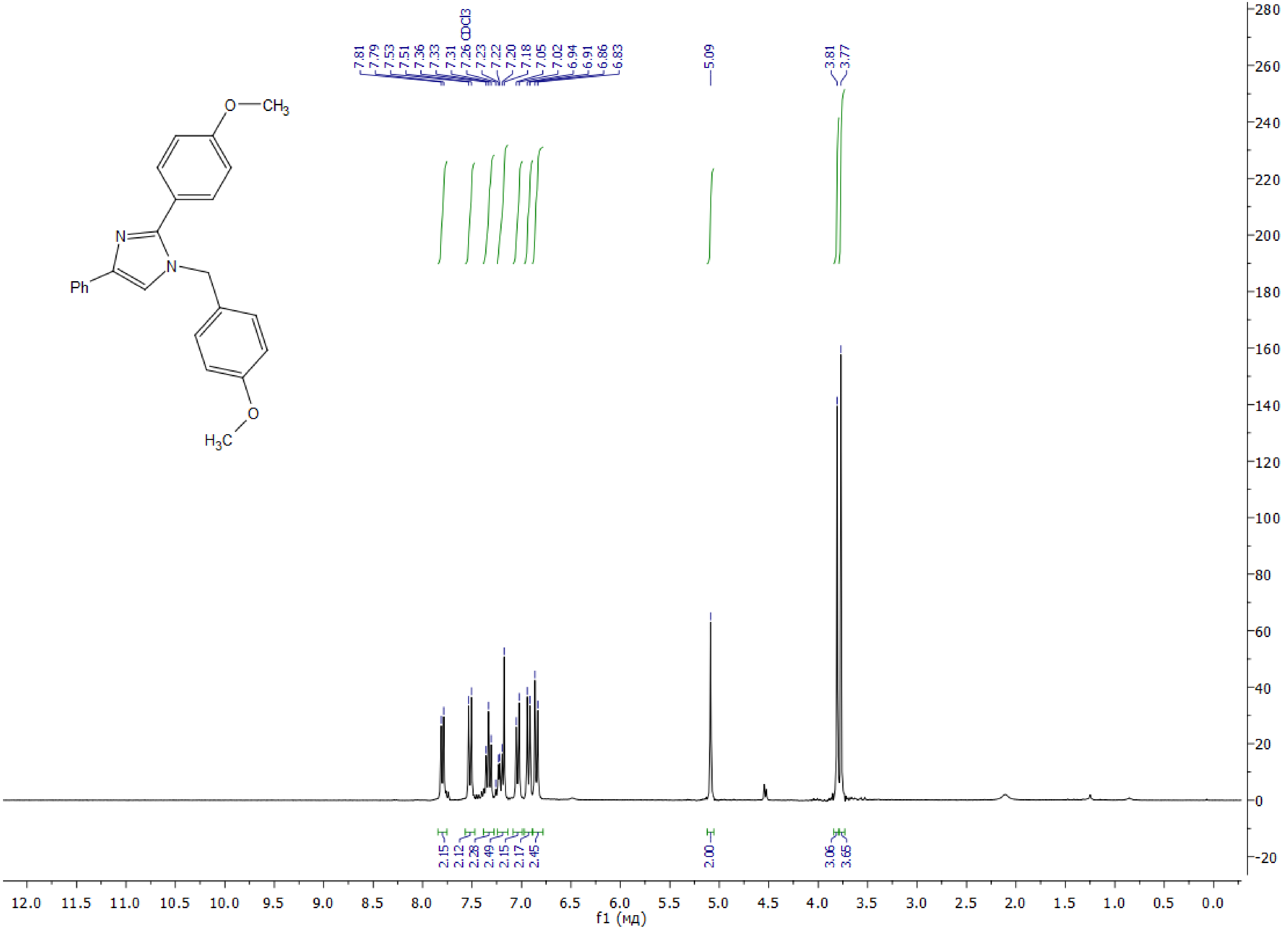

Ph
N
N
O
CH3
O
H3C
7.81
7.79
7.53
7.51
7.36
7.33
7.31
7.26 CDCl3
7.23
7.22
7.20
7.18
7.05
7.02
6.94
6.91
6.86
6.83
5.09
3.81
3.77
2.15
2.12
2.28
2.49
2.15
2.17
2.45
2.00
3.06
3.65
f1 (мд)

^{13}C NMR of 3l

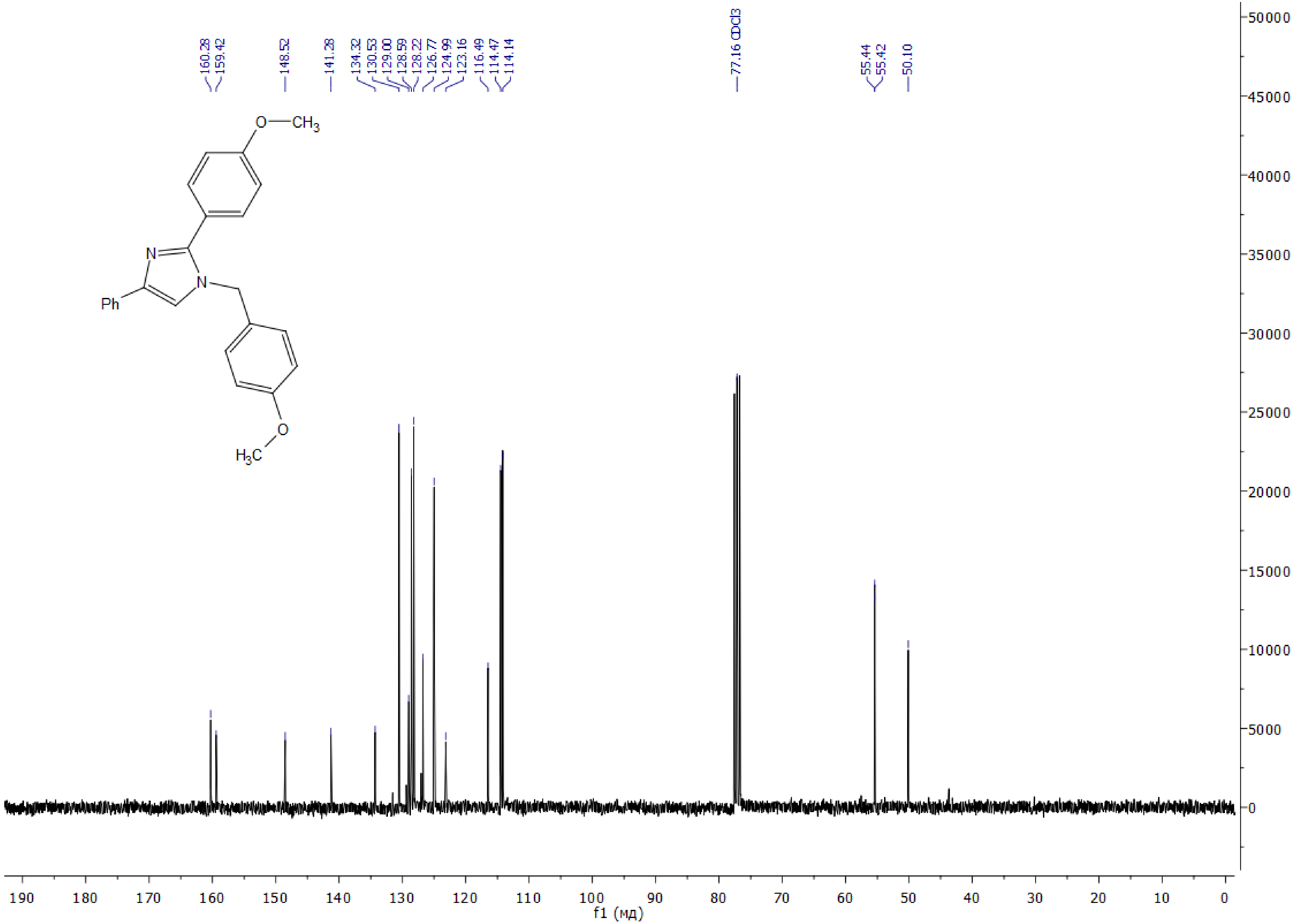

160.28
159.42
148.52
141.28
134.32
130.53
129.00
128.59
128.22
126.77
124.99
123.16
116.49
114.47
114.14
77.16 CDCl3
55.44
55.42
50.10
O—CH3
N
N
Ph
O
H3C
50000
45000
40000
35000
30000
25000
20000
15000
10000
5000
0
190
180
170
160
150
140
130
120
110
100
90
80
70
60
50
40
30
20
10
0
f1 (мд)

^{1}H NMR of 3m

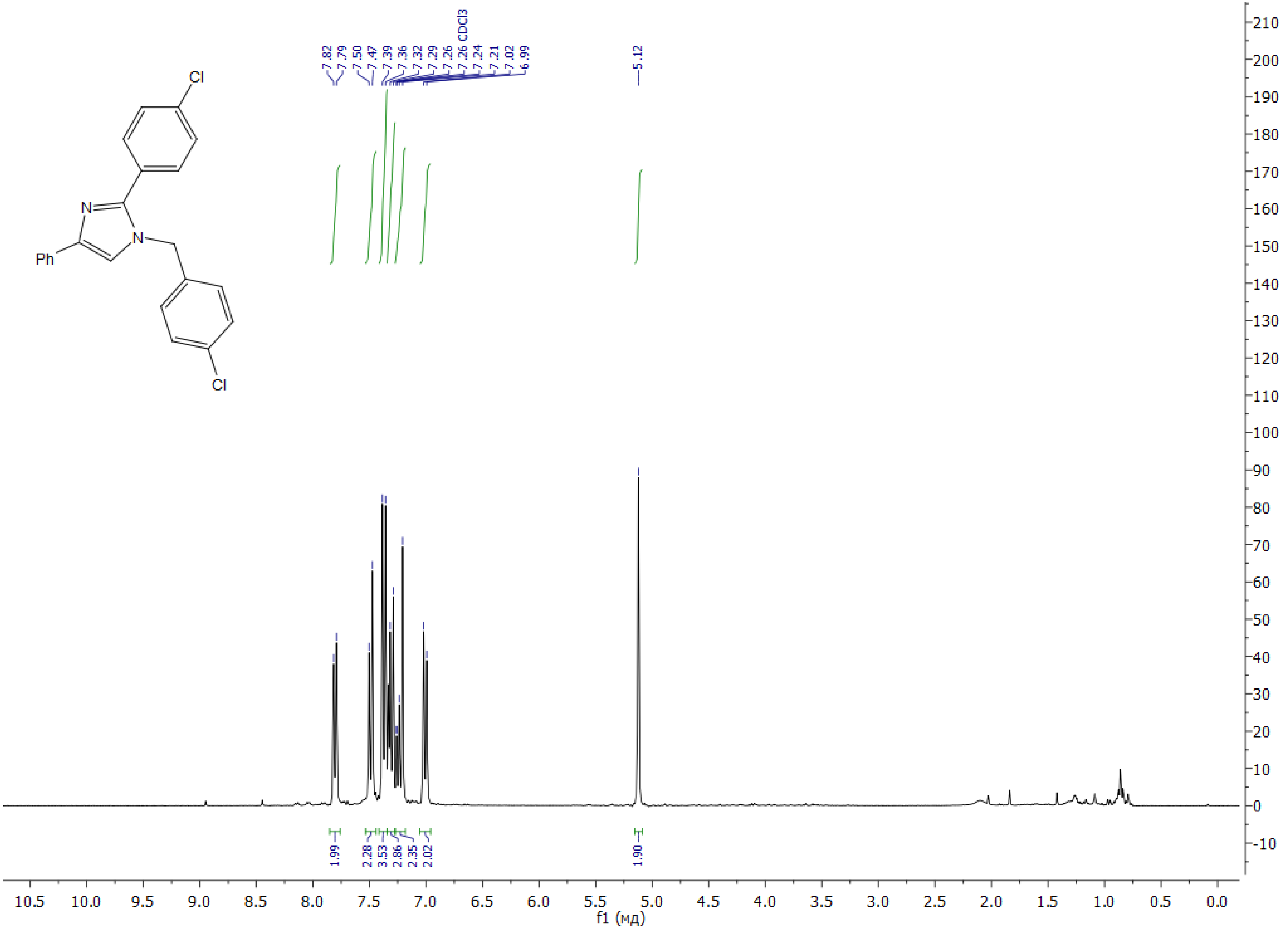
Cl
N
N
Ph
Cl
7.82
7.79
7.50
7.47
7.39
7.36
7.32
7.29
7.26
7.26 CDCl3
7.24
7.21
7.02
6.99
5.12
1.99
2.28
3.53
2.86
2.35
2.02
1.90
f1 (мд)

^{13}C NMR of 3m

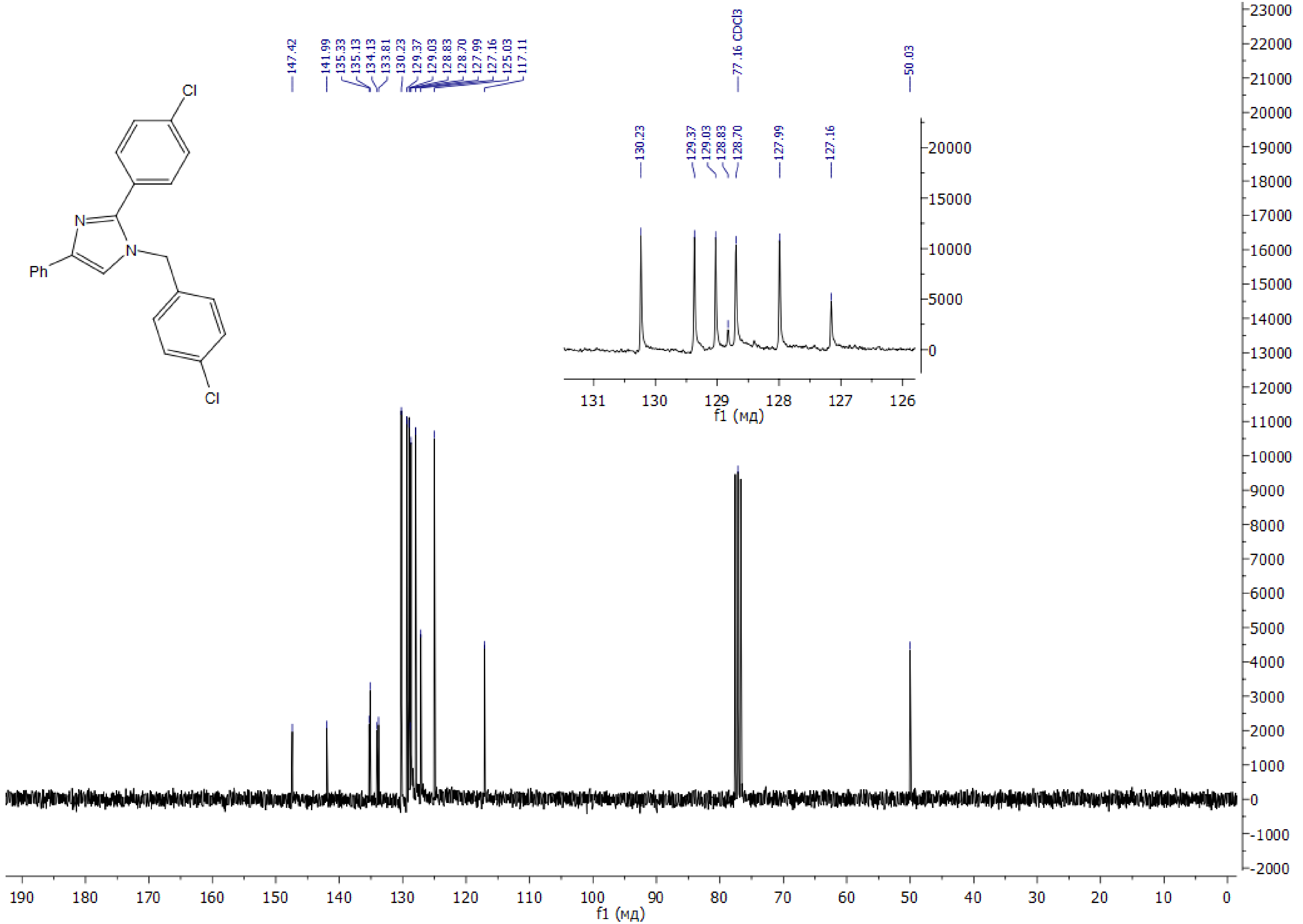
147.42
141.99
135.33
135.13
134.13
133.81
130.23
129.37
129.03
128.83
128.70
127.99
127.16
125.03
117.11
77.16 CDCl3
50.03
Cl
N
N
Ph
Cl
f1 (мд)

^{1}H NMR of 3n

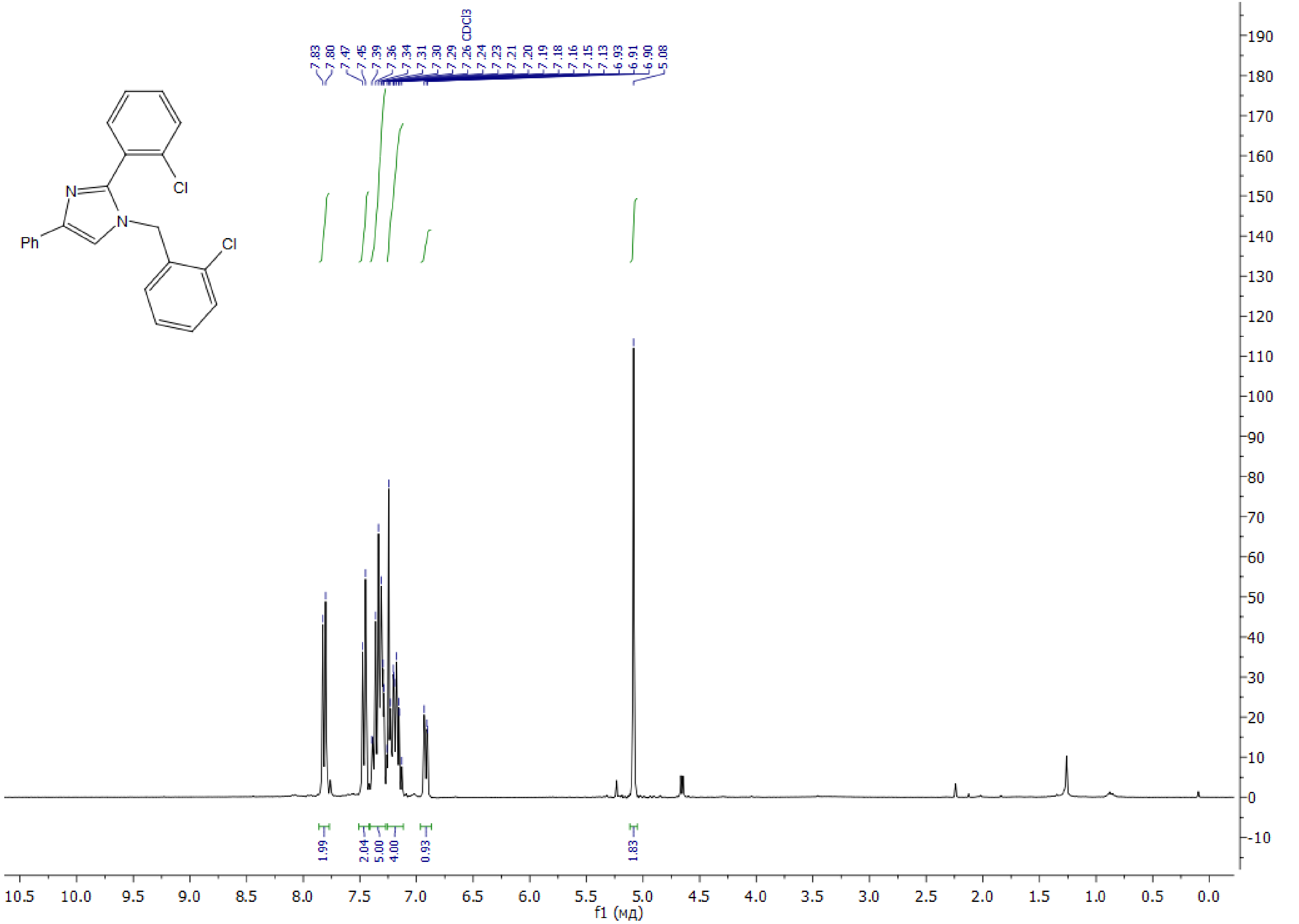

7.83
7.80
7.47
7.45
7.39
7.36
7.34
7.31
7.30
7.29
7.26 CDCl3
7.24
7.23
7.21
7.20
7.19
7.18
7.16
7.15
7.13
6.93
6.91
6.90
5.08
N
N
Cl
Cl
Ph
1.99
2.04
5.00
4.00
0.93
1.83
f1 (мд)

^{13}C NMR of 3n

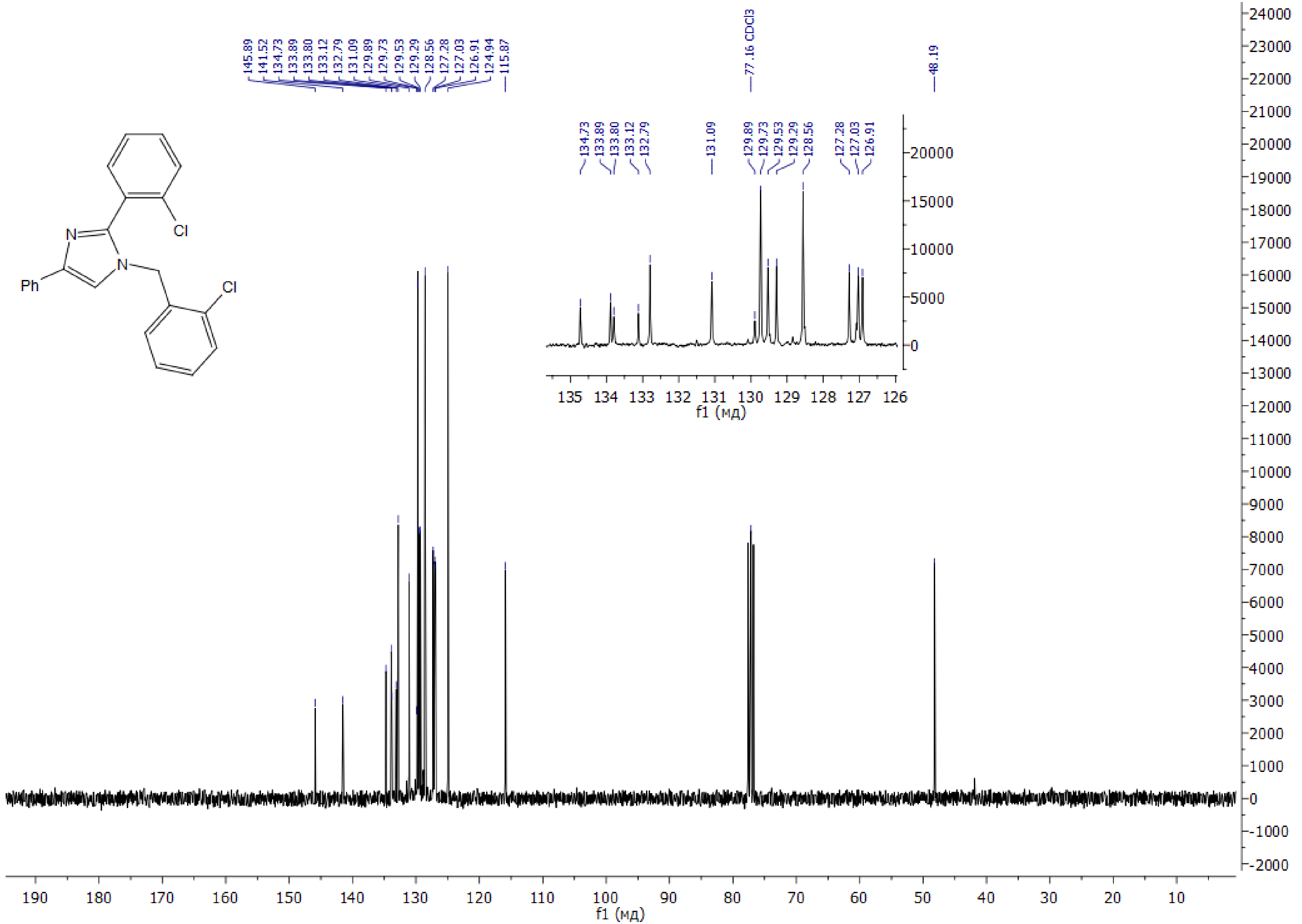

N
N
Cl
Cl
Ph
145.89
141.52
134.73
133.89
133.80
133.12
132.79
131.09
129.89
129.73
129.53
129.29
128.56
127.28
127.03
126.91
124.94
115.87
77.16 CDCl3
48.19
f1 (мд)

^{1}H NMR of 3o

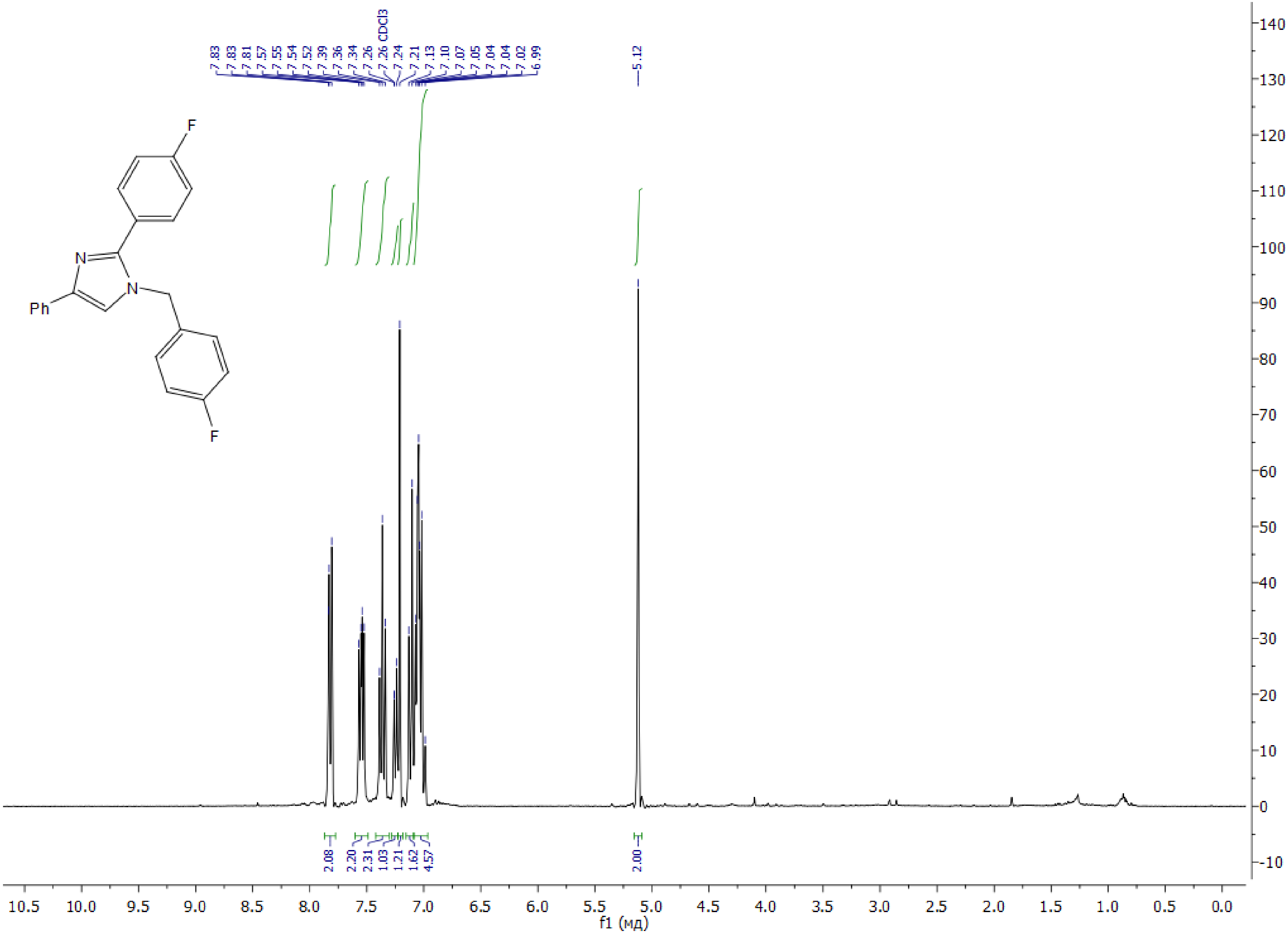

F
N
N
Ph
F
7.83
7.83
7.81
7.57
7.55
7.54
7.52
7.39
7.36
7.34
7.26
7.26 CDCl3
7.24
7.21
7.13
7.10
7.07
7.05
7.04
7.04
7.02
6.99
5.12
2.08
2.20
2.31
1.03
1.21
1.62
4.57
2.00
f1 (мд)

^{13}C NMR of 3o

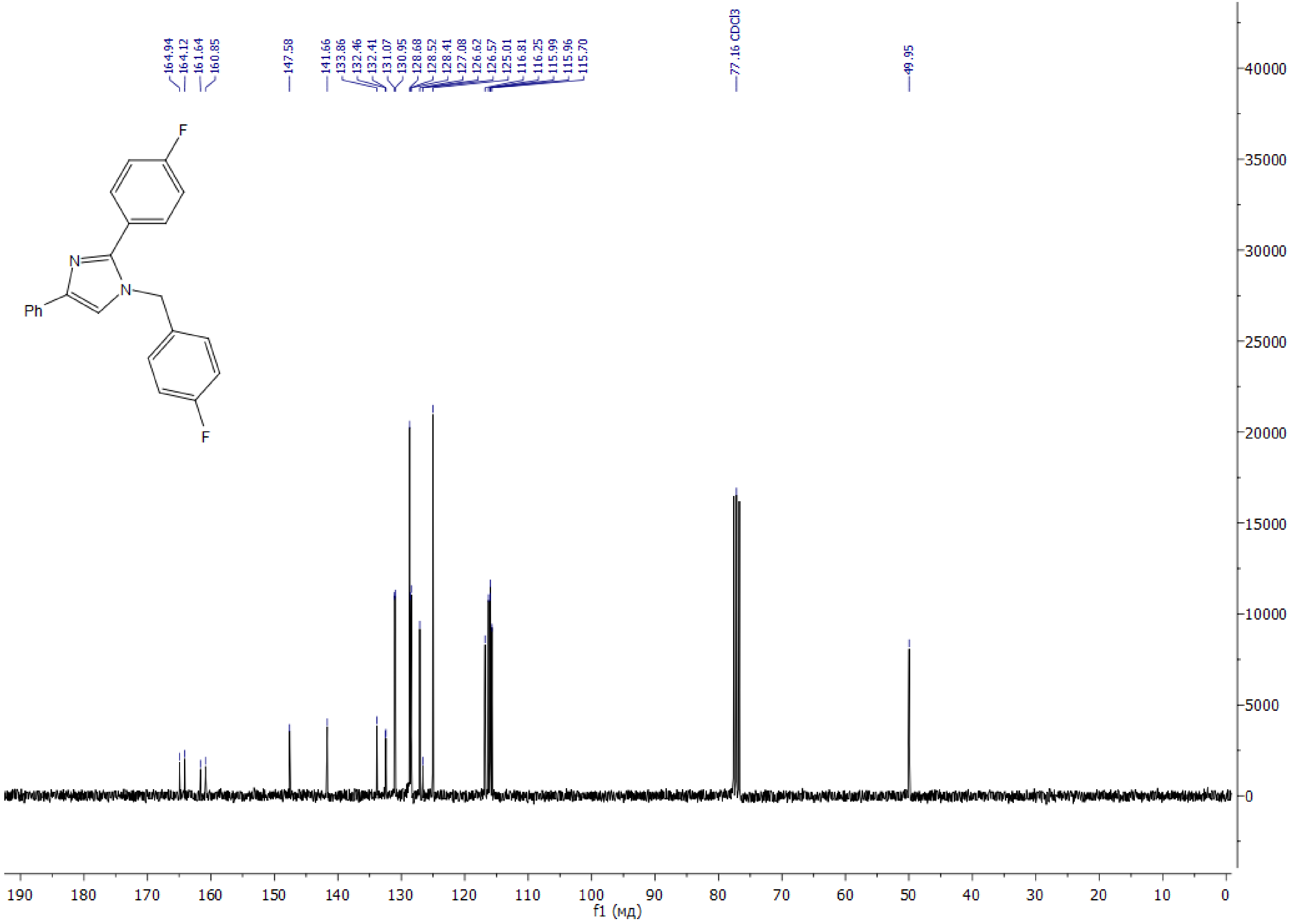

164.94
164.12
161.64
160.85
147.58
141.66
133.86
132.46
132.41
131.07
130.95
128.68
128.52
128.41
127.08
126.62
126.57
125.01
116.81
116.25
115.99
115.96
115.70
77.16 CDCl3
49.95
F
N
N
Ph
F
f1 (мд)

^{19}F NMR of 3o

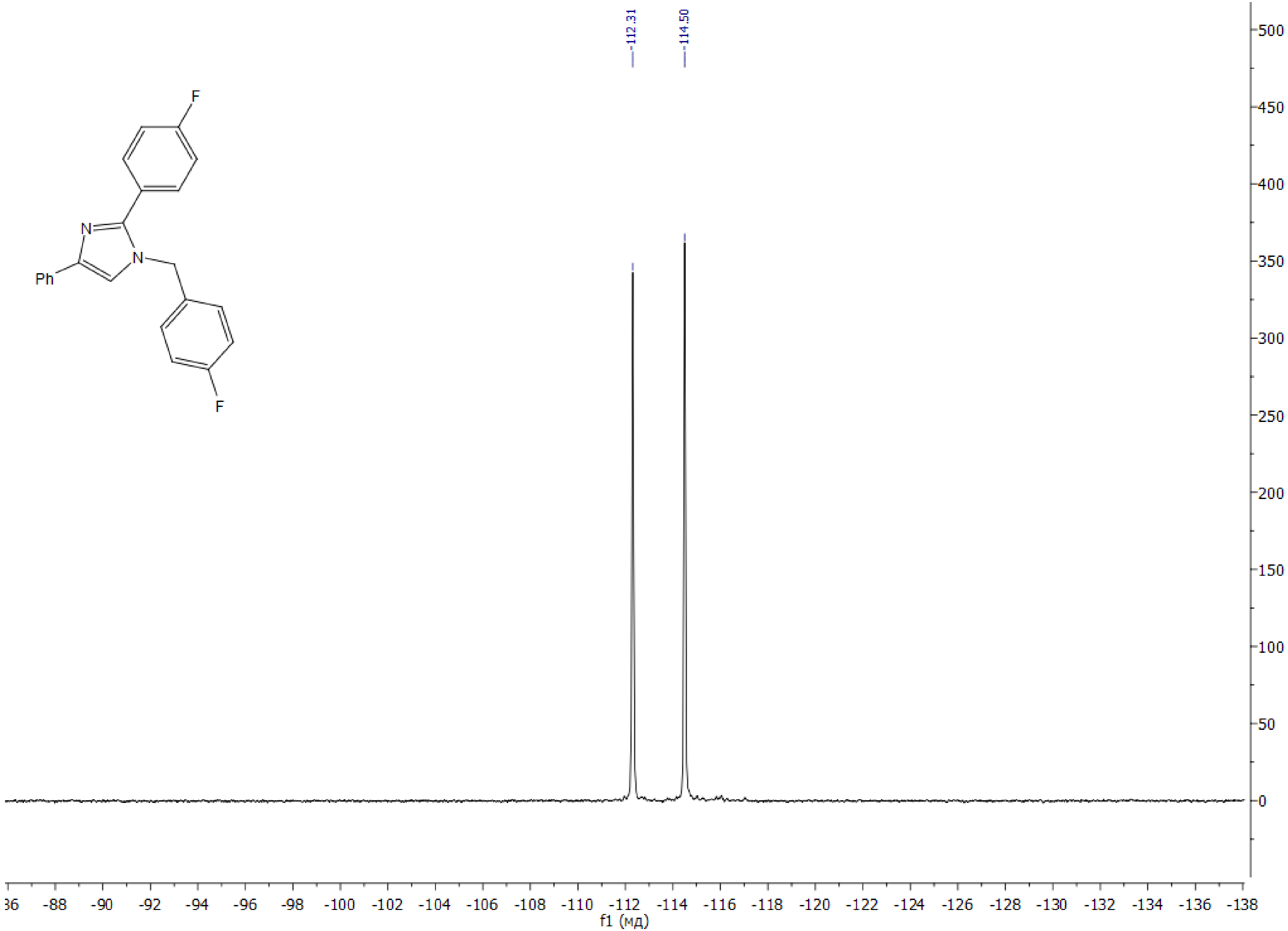

-112.31
-114.50
F
N
N
Ph
F
500
450
400
350
300
250
200
150
100
50
0
86
-88
-90
-92
-94
-96
-98
-100
-102
-104
-106
-108
-110
-112
-114
-116
-118
-120
-122
-124
-126
-128
-130
-132
-134
-136
-138
f1 (мд)

^{1}H NMR of 3p

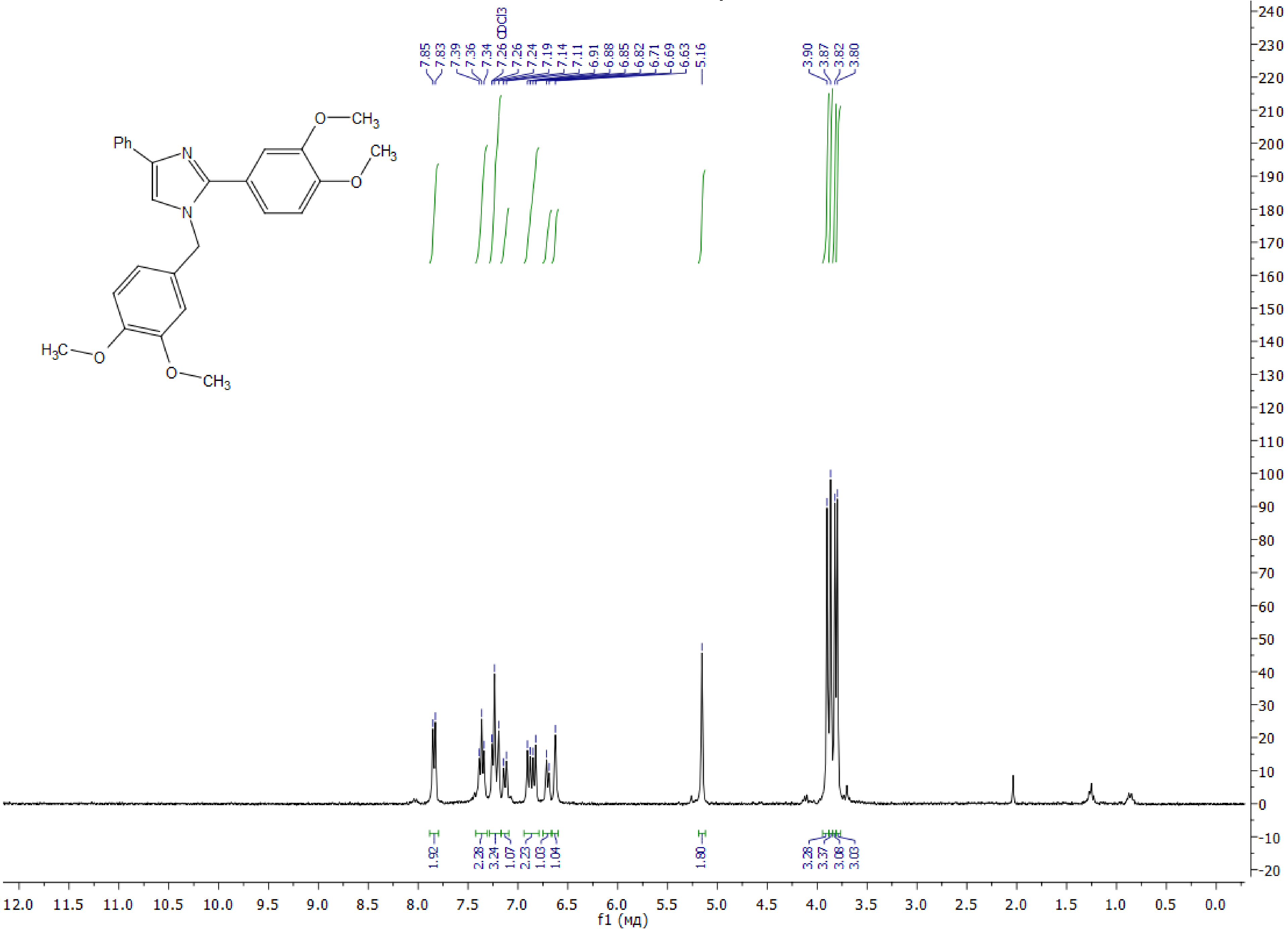

Ph
N
N
O
CH3
O
CH3
H3C
O
O
CH3
CDCl3
7.85
7.83
7.39
7.36
7.34
7.26
7.26
7.24
7.19
7.14
7.11
6.91
6.88
6.85
6.82
6.71
6.69
6.63
5.16
3.90
3.87
3.82
3.80
1.92
2.28
3.24
1.07
2.23
1.03
1.04
1.80
3.28
3.37
3.08
3.03
f1 (мд)

^{13}C NMR of 3p

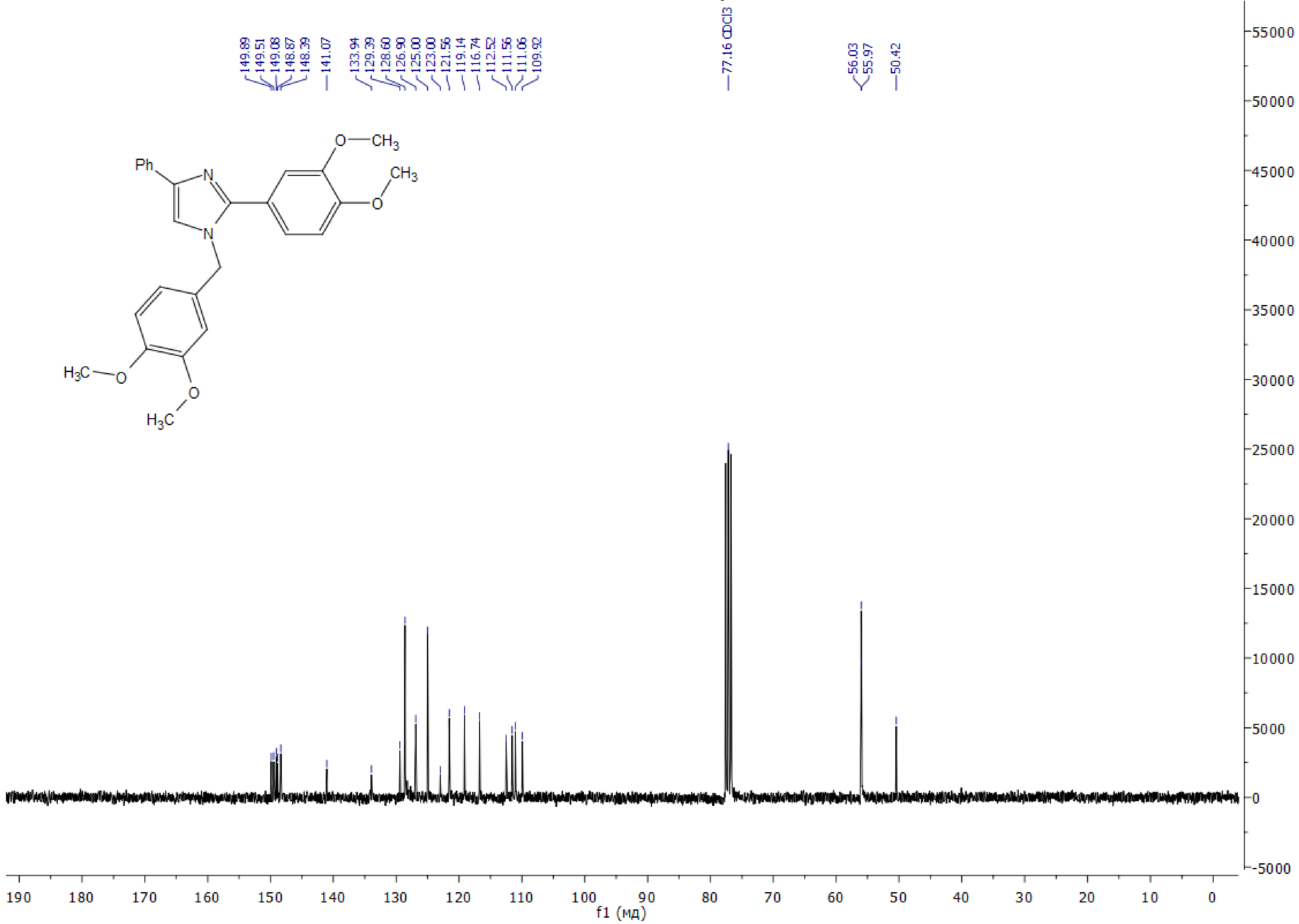
149.89
149.51
149.08
148.87
148.39
141.07
133.94
129.39
128.60
126.90
125.00
123.00
121.56
119.14
116.74
112.52
111.56
111.06
109.92
77.16 CDCl3
56.03
55.97
50.42
Ph
N
N
O
CH3
O
CH3
H3C
O
O
H3C
f1 (мд)

^{1}H NMR of 3q

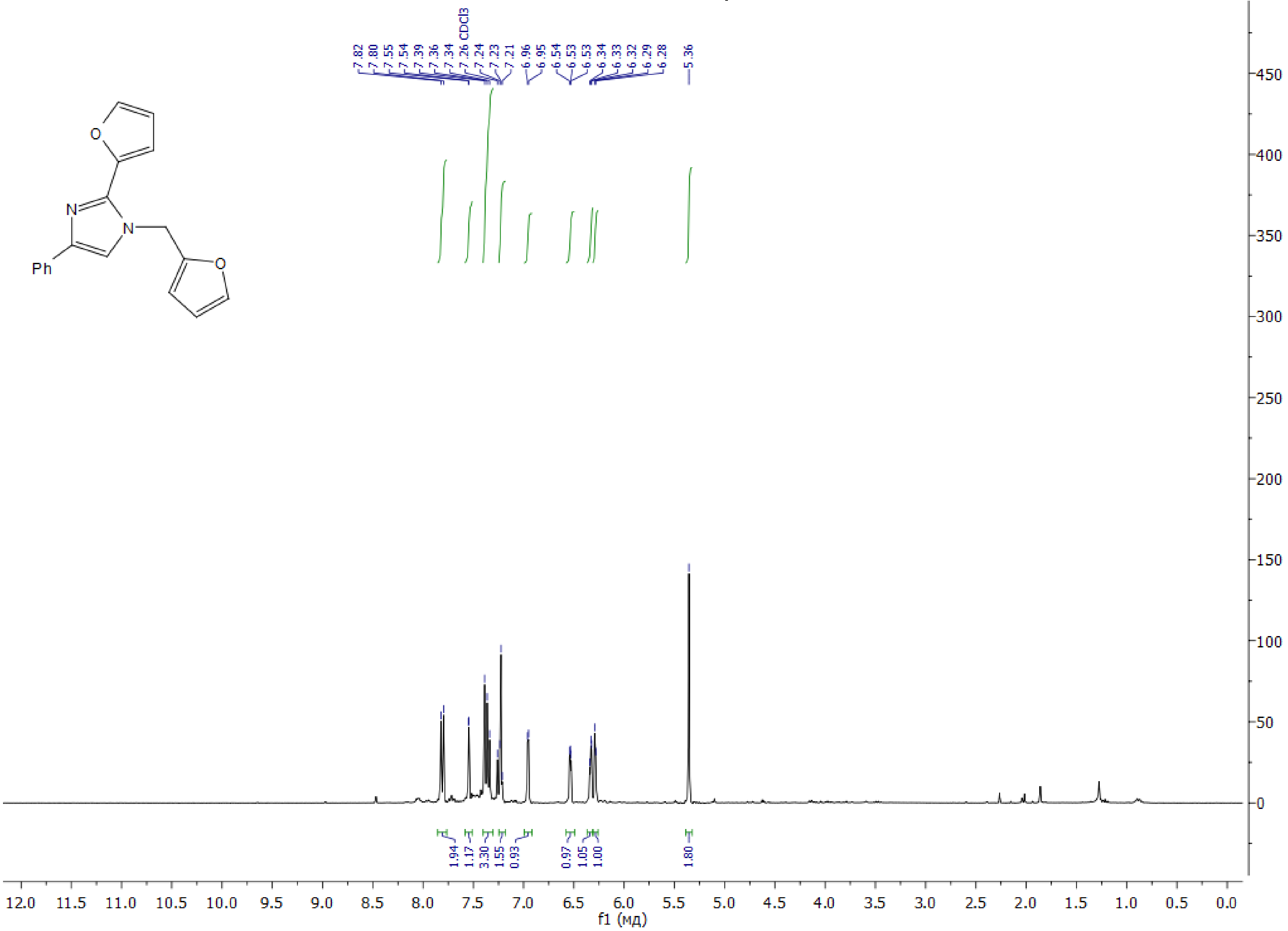

O
N
N
O
Ph
7.82
7.80
7.55
7.54
7.39
7.36
7.34
7.26 CDCl3
7.24
7.23
7.21
6.96
6.95
6.54
6.53
6.53
6.34
6.33
6.32
6.29
6.28
5.36
1.94
1.17
3.30
1.55
0.93
0.97
1.05
1.00
1.80
f1 (мд)

^{13}C NMR of 3q

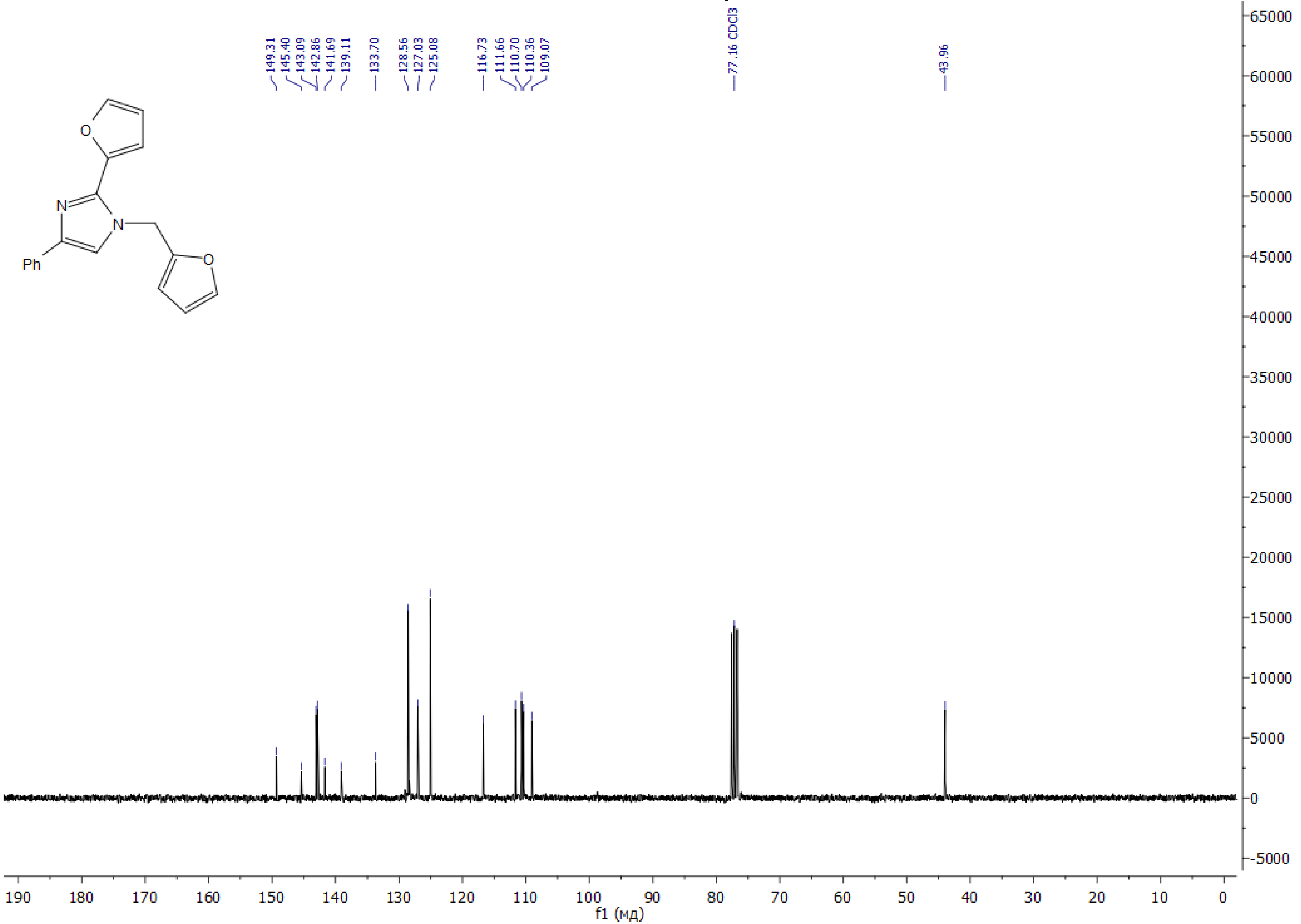

149.31
145.40
143.09
142.86
141.69
139.11
133.70
128.56
127.03
125.08
116.73
111.66
110.70
110.36
109.07
77.16 CDCl3
43.96
O
N
N
Ph
O
65000
60000
55000
50000
45000
40000
35000
30000
25000
20000
15000
10000
5000
0
-5000
190
180
170
160
150
140
130
120
110
100
90
80
70
60
50
40
30
20
10
0
f1 (мд)

^{1}H NMR of 3r

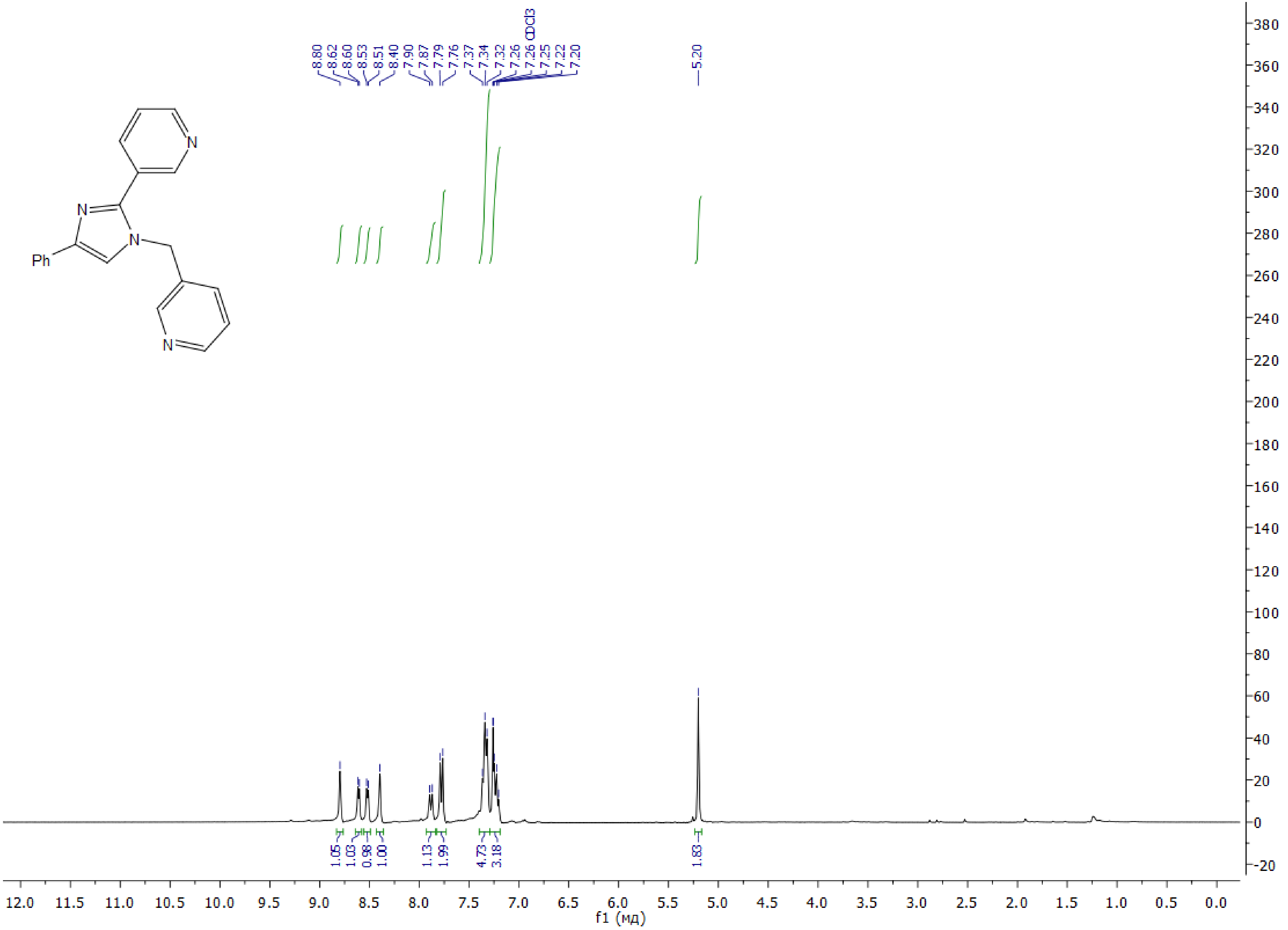
N
N
N
Ph
N
8.80
8.62
8.60
8.53
8.51
8.40
7.90
7.87
7.79
7.76
7.37
7.34
7.32
7.26
7.26 CDCl3
7.25
7.22
7.20
5.20
1.05
1.03
0.98
1.00
1.13
1.99
4.73
3.18
1.83
f1 (мд)

^{13}C NMR of 3r

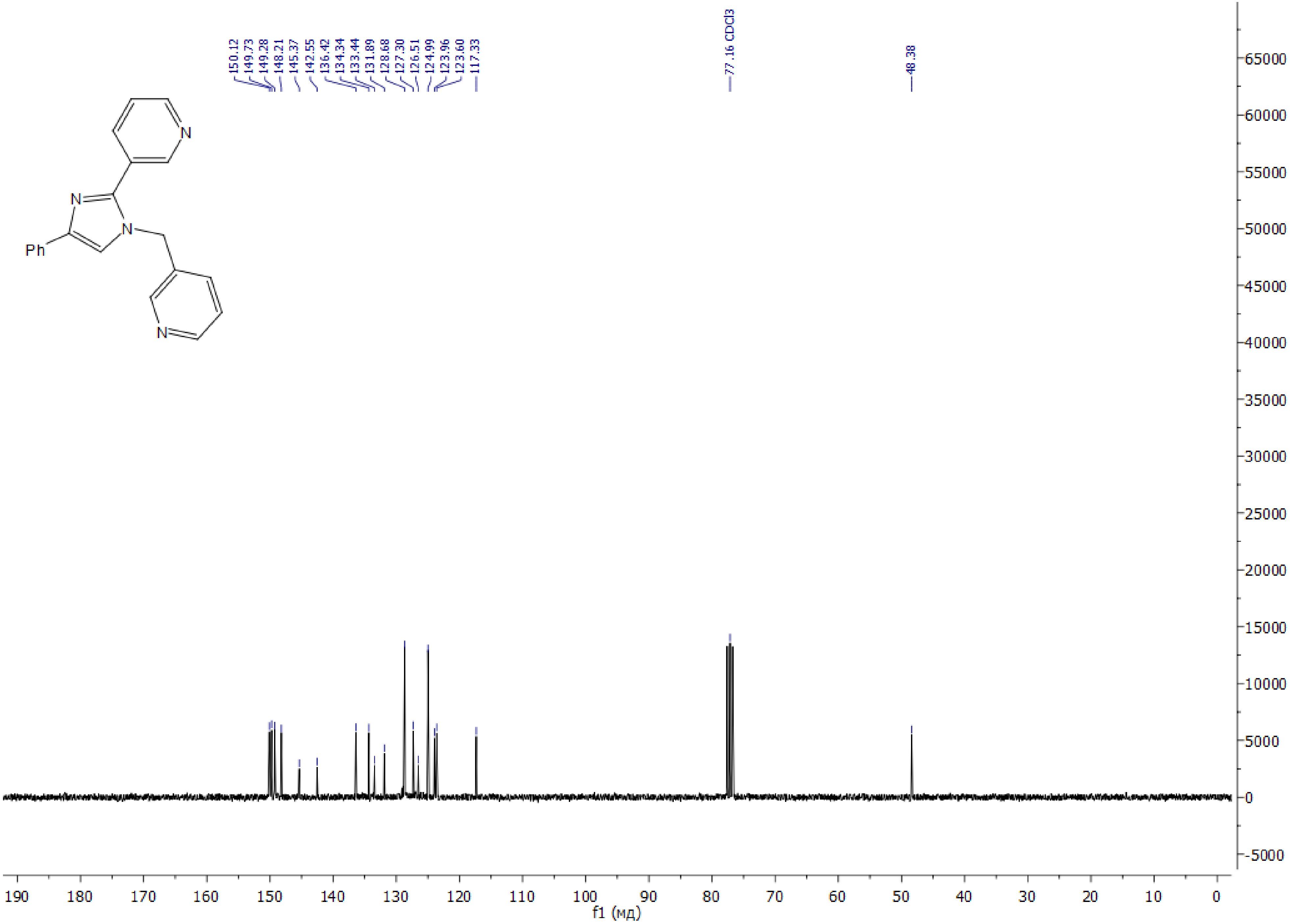

150.12
149.73
149.28
148.21
145.37
142.55
136.42
134.34
133.44
131.89
128.68
127.30
126.51
124.99
123.96
123.60
117.33
77.16 CDCl3
48.38
N
N
N
Ph
N
f1 (мд)

HRMS spectra of synthesized compounds

HRMS of 3a

Display Report

Analysis Info

Analysis Name	D:\Data\Chizhov\Terent'ev\Vil'\sb-526_&clblow.d	Acquisition Date	01.11.2022 12:45:26
Method	tune_low_1550.m	Operator	BDAL@DE
Sample Name	/TERN SB-526	Instrument / Ser#	maXis 43
Comment	CH3CN 100 %, dil. 2000, calibrant added		

Acquisition Parameter

Source Type	ESI	Ion Polarity	Positive	Set Nebulizer	0.4 Bar
Focus	Not active			Set Dry Heater	180 °C
Scan Begin	50 m/z	Set Capillary	4500 V	Set Dry Gas	4.0 l/min
Scan End	1550 m/z	Set End Plate Offset	-500 V	Set Divert Valve	Source

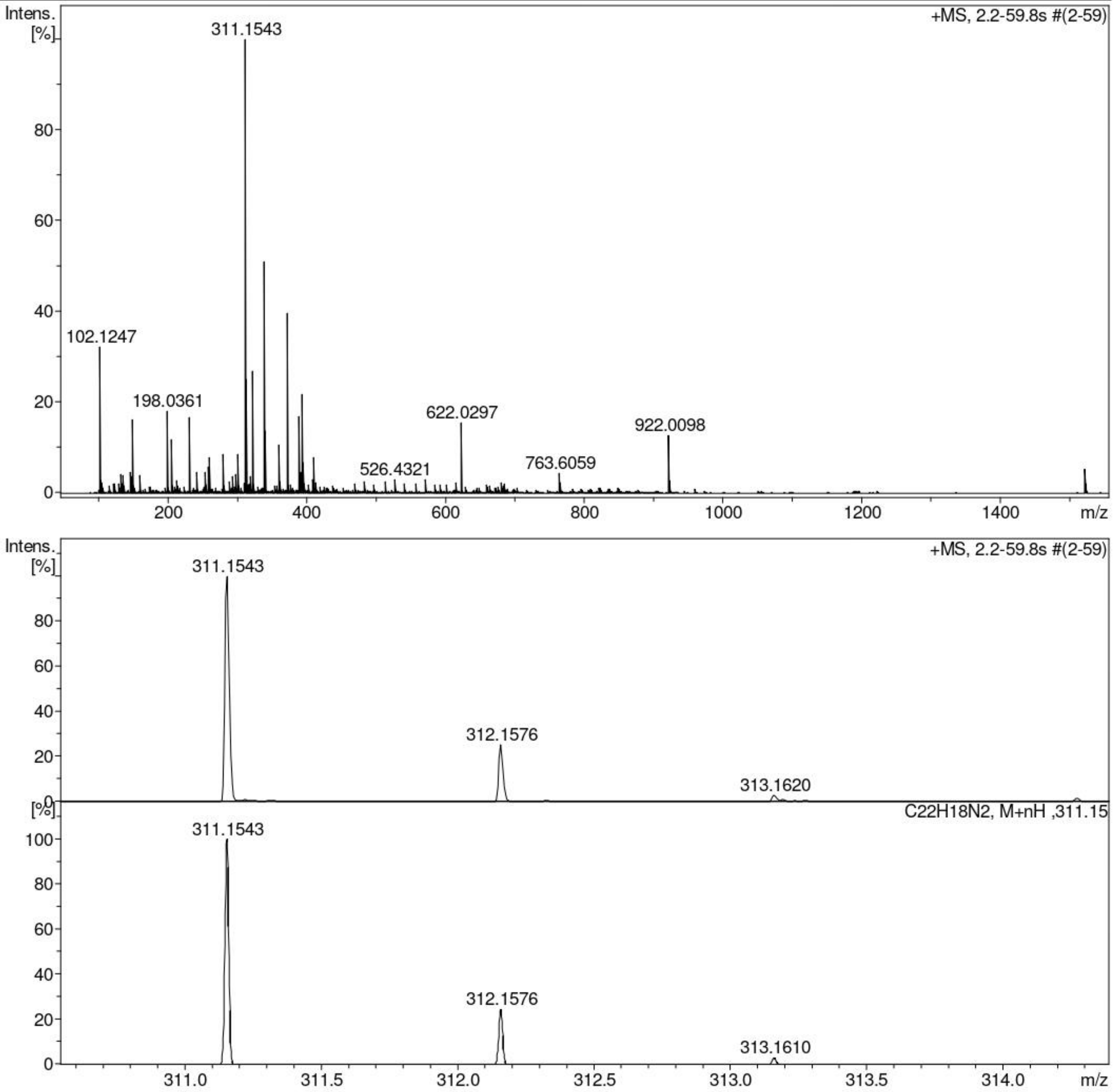

Bruker Compass DataAnalysis 4.0 printed: 01.11.2022 13:38:54 Page 1 of 1

HRMS of 3b

Display Report

Analysis Info

Analysis Name	D:\Data\Chizhov\Terent'ev\Vil'\sb-533_&clblow.d	Acquisition Date	01.11.2022 12:59:14
Method	tune_low_1550.m	Operator	BDAL@DE
Sample Name	/TERN SB-533	Instrument / Ser#	maXis 43
Comment	CH3CN 100 %, dil. 2000, calibrant added		

Acquisition Parameter

Source Type	ESI	Ion Polarity	Positive	Set Nebulizer	0.4 Bar
Focus	Not active			Set Dry Heater	180 °C
Scan Begin	50 m/z	Set Capillary	4500 V	Set Dry Gas	4.0 l/min
Scan End	1550 m/z	Set End Plate Offset	-500 V	Set Divert Valve	Source

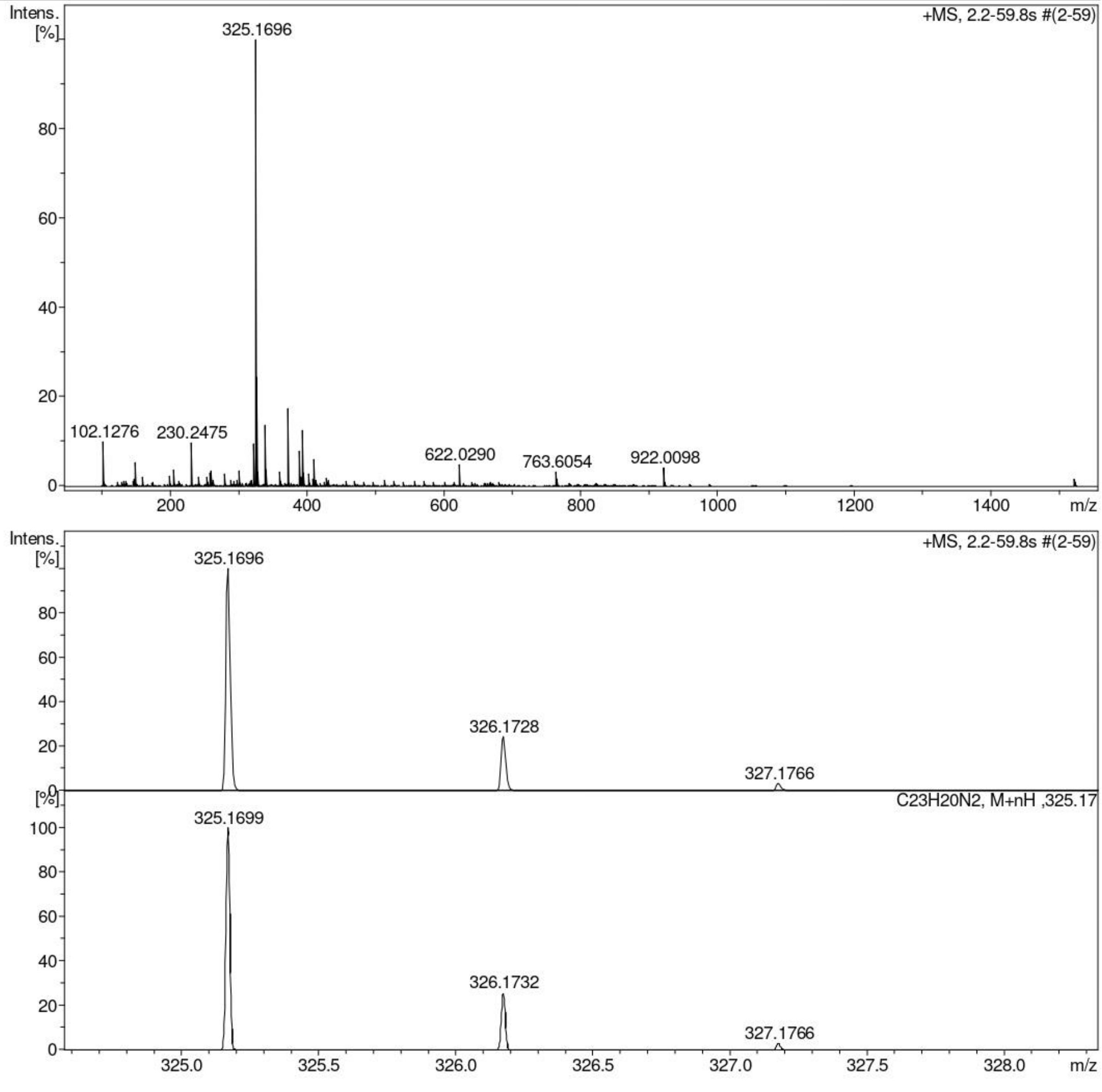

HRMS of 3c

Display Report

Analysis Info

Analysis Name	D:\Data\Chizhov\Terent'ev\Vil'\sb-530_&clblow.d	Acquisition Date	01.11.2022 12:49:57
Method	tune_low_1550.m	Operator	BDAL@DE
Sample Name	/TERN SB-530	Instrument / Ser#	maXis 43
Comment	CH3CN 100 %, dil. 2000, calibrant added		

Acquisition Parameter

Source Type	ESI	Ion Polarity	Positive	Set Nebulizer	0.4 Bar
Focus	Not active			Set Dry Heater	180 °C
Scan Begin	50 m/z	Set Capillary	4500 V	Set Dry Gas	4.0 l/min
Scan End	1550 m/z	Set End Plate Offset	-500 V	Set Divert Valve	Source

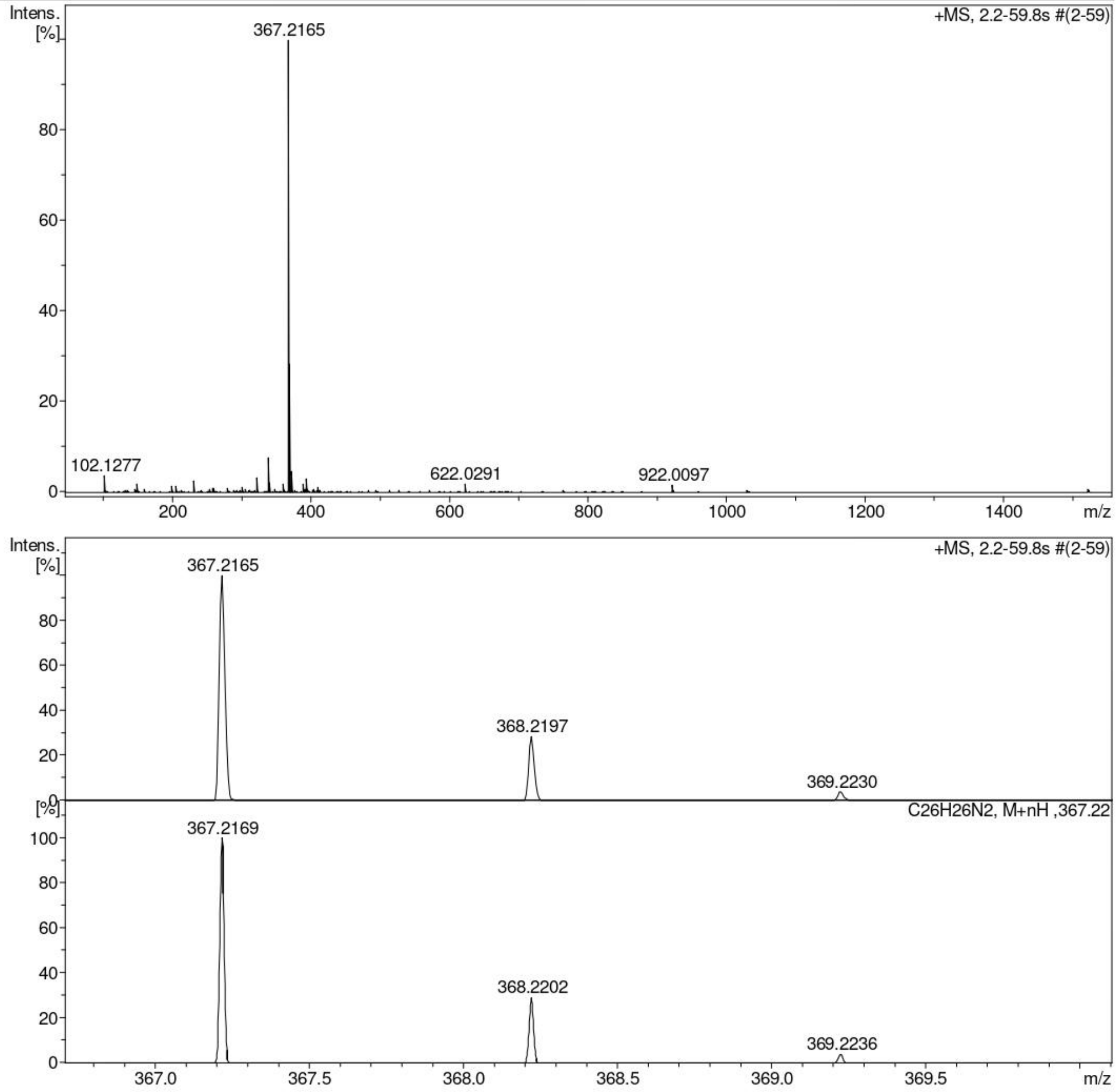

HRMS of 3d

Display Report

Analysis Info

Analysis Name	D:\Data\Chizhov\Terentiev\Wil\sb-536_&clblow.d	Acquisition Date	31.10.2022 17:33:58
Method	tune_low.m	Operator	BDAL@DE
Sample Name	/TERN SB-536	Instrument / Ser#	micrOTOF 10248
Comment	CH3CN 100 %, dil. 2000, calibrant added		

Acquisition Parameter

Source Type	ESI	Ion Polarity	Positive	Set Nebulizer	0.4 Bar
Focus	Not active			Set Dry Heater	180 °C
Scan Begin	50 m/z	Set Capillary	4500 V	Set Dry Gas	4.0 l/min
Scan End	3000 m/z	Set End Plate Offset	-500 V	Set Divert Valve	Waste

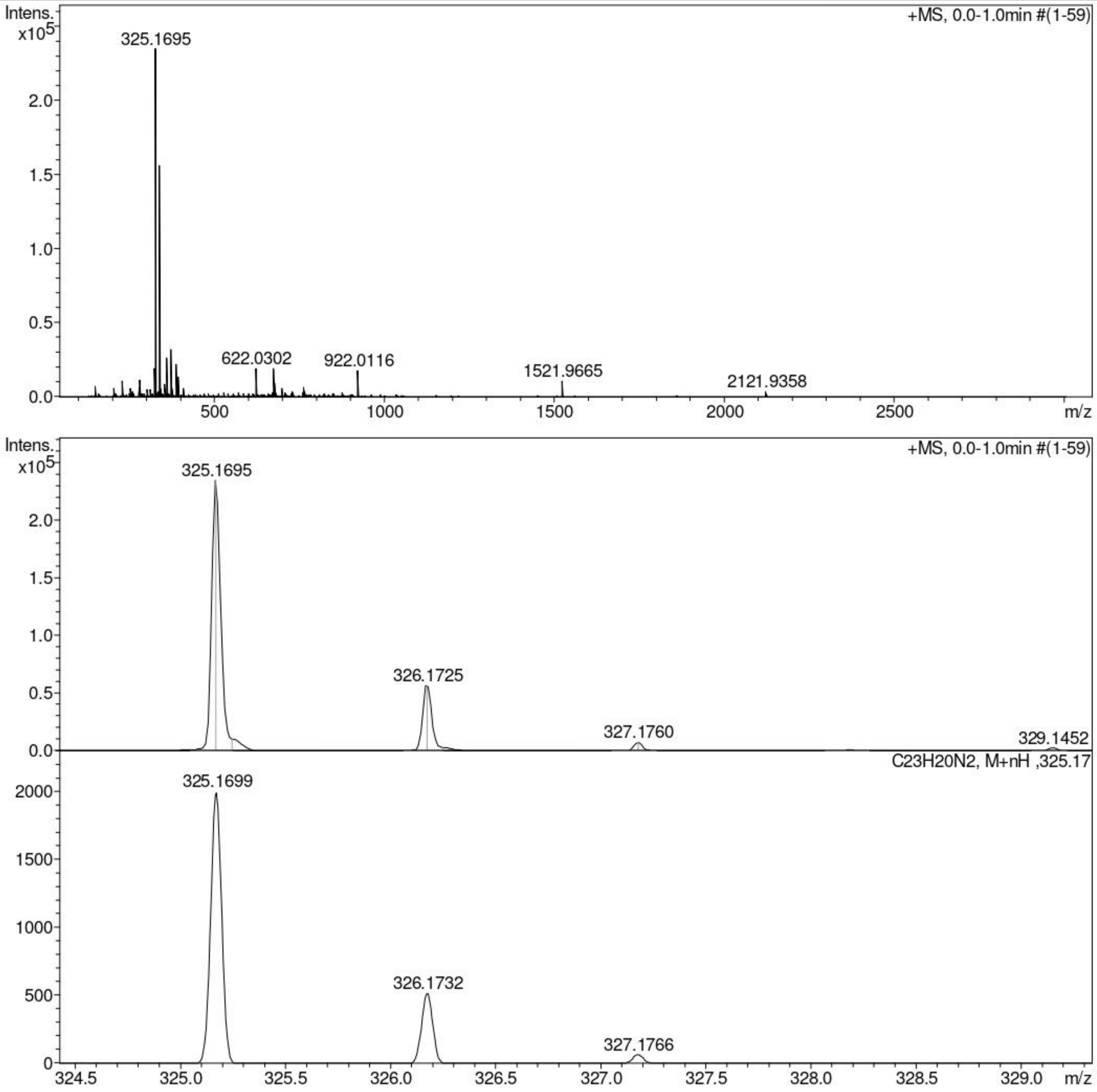

Bruker Compass DataAnalysis 4.0 printed: 31.10.2022 17:42:13 Page 1 of 1

HRMS of 3e

Display Report

Analysis Info

Analysis Name	D:\Data\Kolotyrkina\2022\Vil\1101031.d	Acquisition Date	01.11.2022 17:32:25
Method	tune_low.m	Operator	BDAL@DE
Sample Name	/TERN SG541	Instrument / Ser#	micrOTOF 10248
Comment	C23H20N2O mH 341.1648 calibrant added, CH3CN		

Acquisition Parameter

Source Type	ESI	Ion Polarity	Positive	Set Nebulizer	0.4 Bar
Focus	Not active			Set Dry Heater	180 °C
Scan Begin	50 m/z	Set Capillary	4500 V	Set Dry Gas	4.0 l/min
Scan End	2500 m/z	Set End Plate Offset	-500 V	Set Divert Valve	Waste

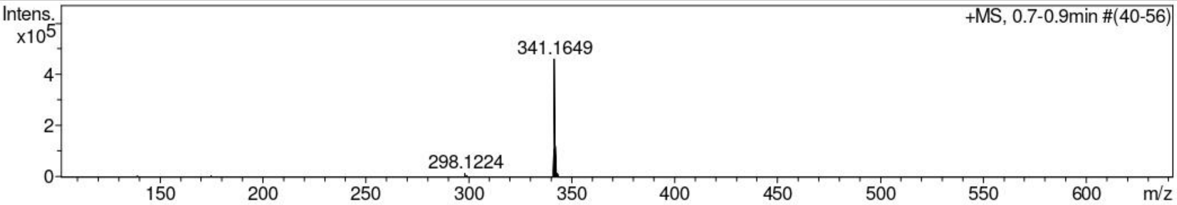

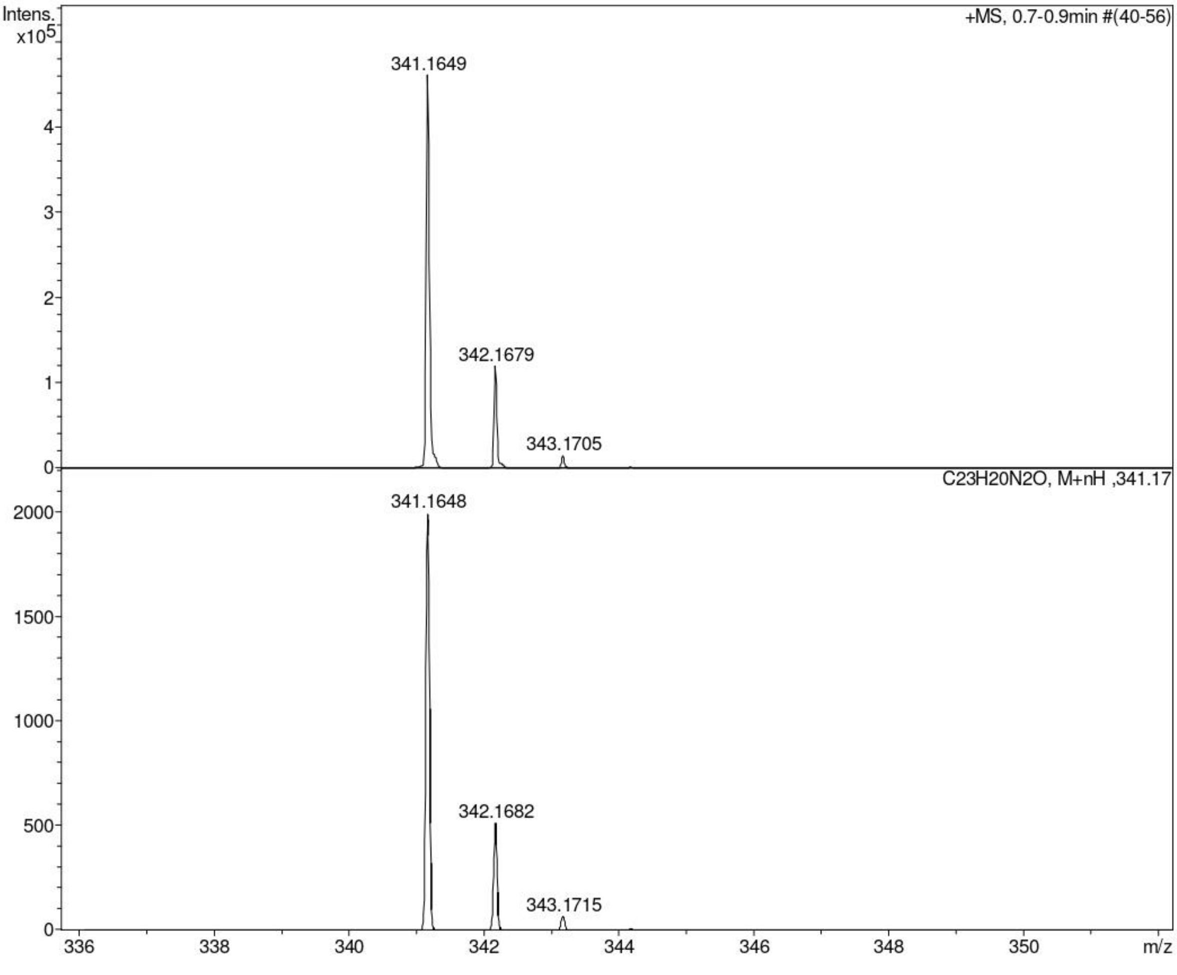

Bruker Compass DataAnalysis 4.0 printed: 01.11.2022 17:35:13 Page 1 of 1

Display Report

Analysis Info

Analysis Name	D:\Data\Chizhov\Terentiev\Wil\sb-512_&clblow.d	Acquisition Date	31.10.2022 17:29:44
Method	tune_low.m	Operator	BDAL@DE
Sample Name	/TERN SB-512	Instrument / Ser#	micrOTOF 10248
Comment	CH3CN 100 %, dil. 2000, calibrant added		

Acquisition Parameter

Source Type	ESI	Ion Polarity	Positive	Set Nebulizer	0.4 Bar
Focus	Not active			Set Dry Heater	180 °C
Scan Begin	50 m/z	Set Capillary	4500 V	Set Dry Gas	4.0 l/min
Scan End	3000 m/z	Set End Plate Offset	-500 V	Set Divert Valve	Waste

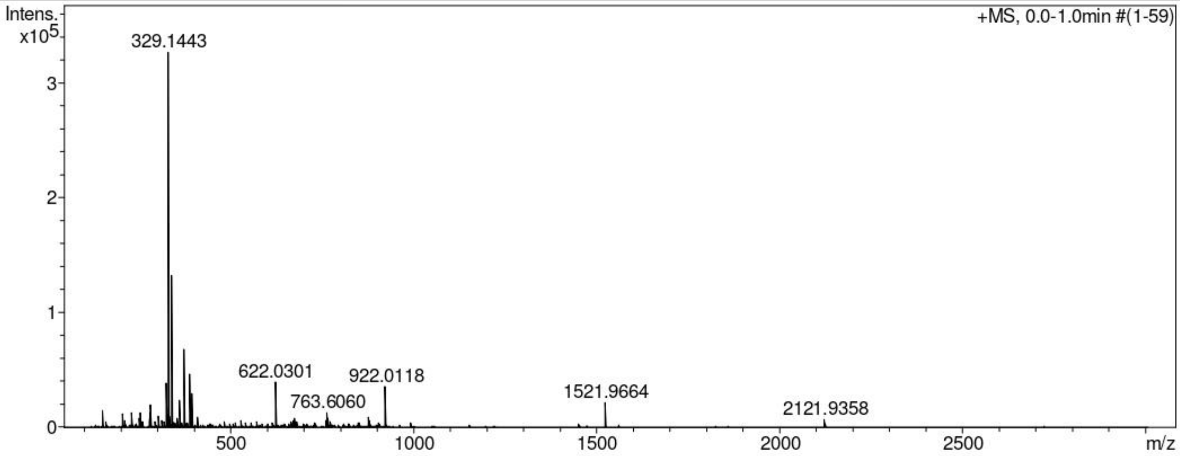

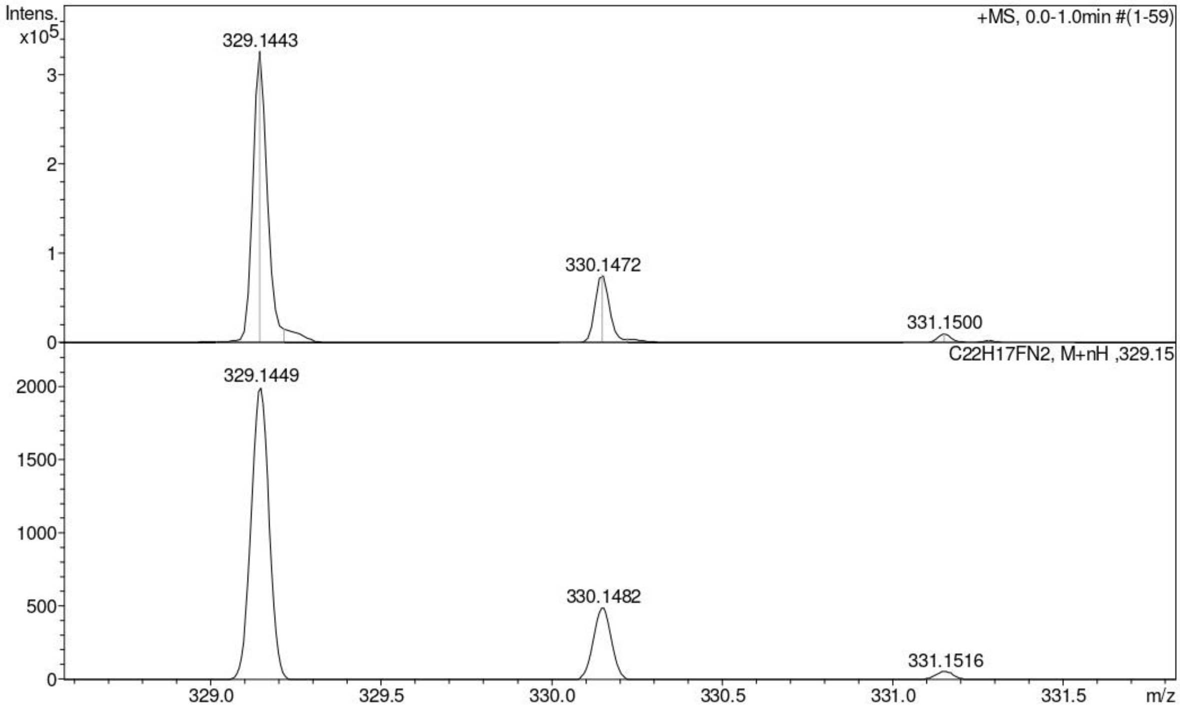

HRMS of 3g

Display Report

Analysis Info

Analysis Name	D:\Data\Chizhov\Terent'ev\Vil'\sb-519_&clblow.d	Acquisition Date	01.11.2022 12:41:19
Method	tune_low_1550.m	Operator	BDAL@DE
Sample Name	/TERN SB-519	Instrument / Ser#	maXis 43
Comment	CH3CN 100 %, dil. 2000, calibrant added		

Acquisition Parameter

Source Type	ESI	Ion Polarity	Positive	Set Nebulizer	0.4 Bar
Focus	Not active			Set Dry Heater	180 °C
Scan Begin	50 m/z	Set Capillary	4500 V	Set Dry Gas	4.0 l/min
Scan End	1550 m/z	Set End Plate Offset	-500 V	Set Divert Valve	Source

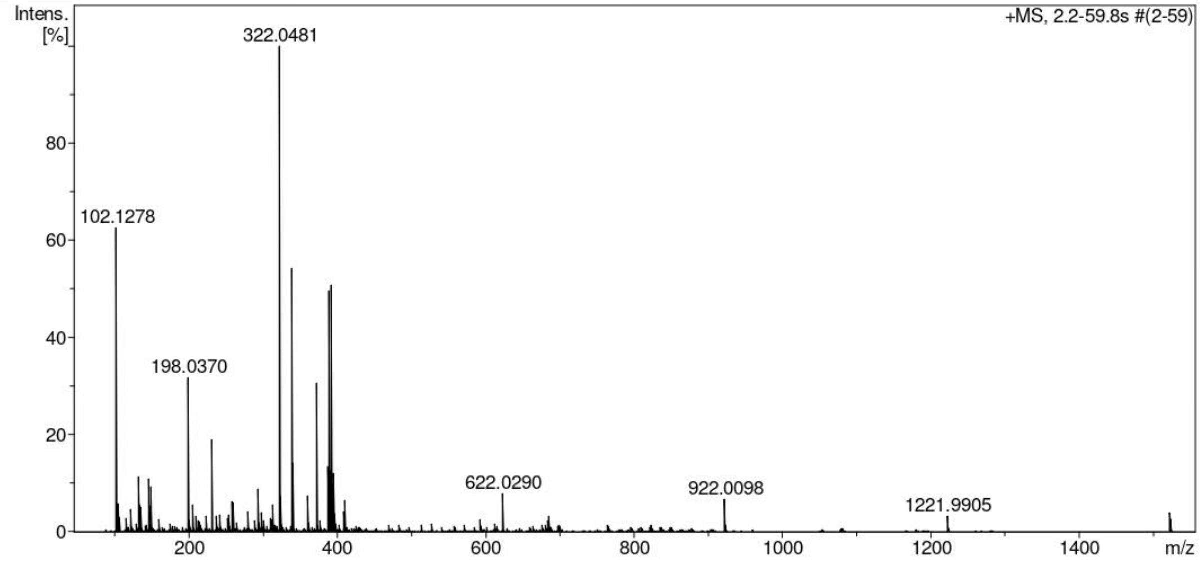

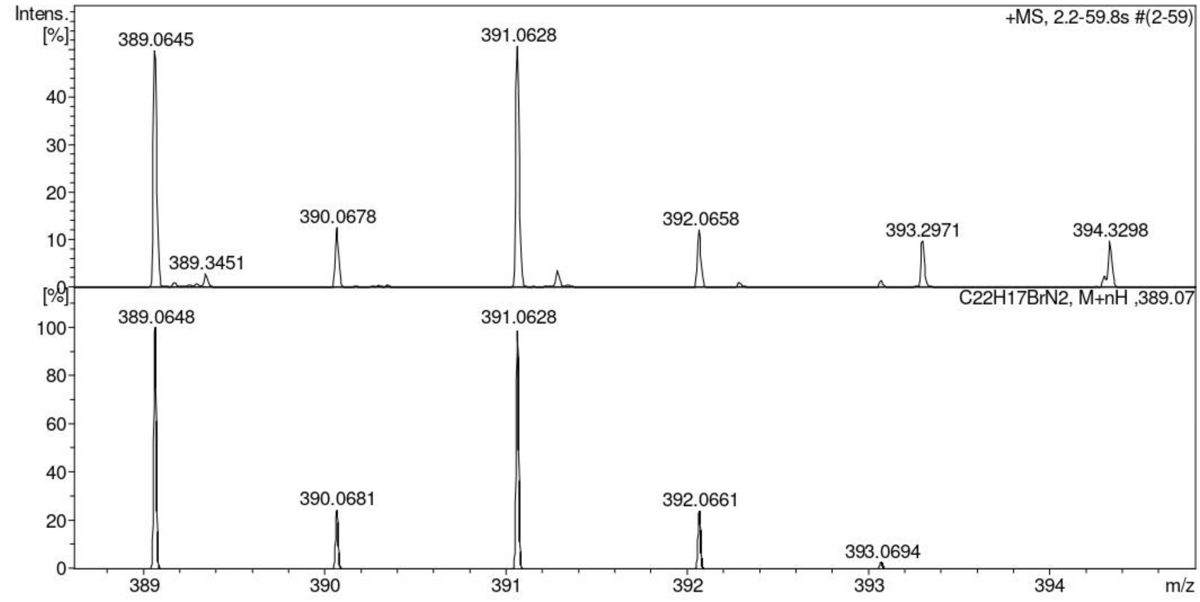

Bruker Compass DataAnalysis 4.0 printed: 01.11.2022 13:37:11 Page 1 of 1

Display Report

Analysis Info

Analysis Name	D:\Data\Chizhov\Terentiev\Wil\sg-550_&clblow.d	Acquisition Date	30.06.2022 16:34:43
Method	tune_low.m	Operator	BDAL@DE
Sample Name	/TERN SG-550	Instrument / Ser#	micrOTOF 10248
Comment	CH3CN 100 %, dil. 20000, calibrant added		

Acquisition Parameter

Source Type	ESI	Ion Polarity	Positive	Set Nebulizer	0.4 Bar
Focus	Not active			Set Dry Heater	180 °C
Scan Begin	50 m/z	Set Capillary	4500 V	Set Dry Gas	4.0 l/min
Scan End	3000 m/z	Set End Plate Offset	-500 V	Set Divert Valve	Waste

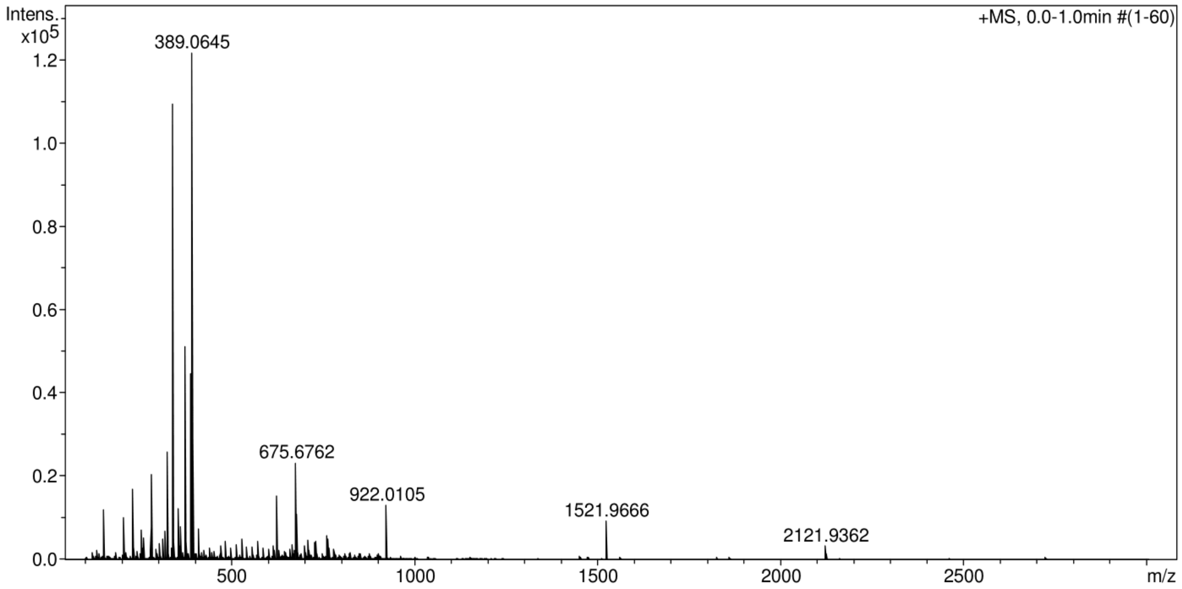

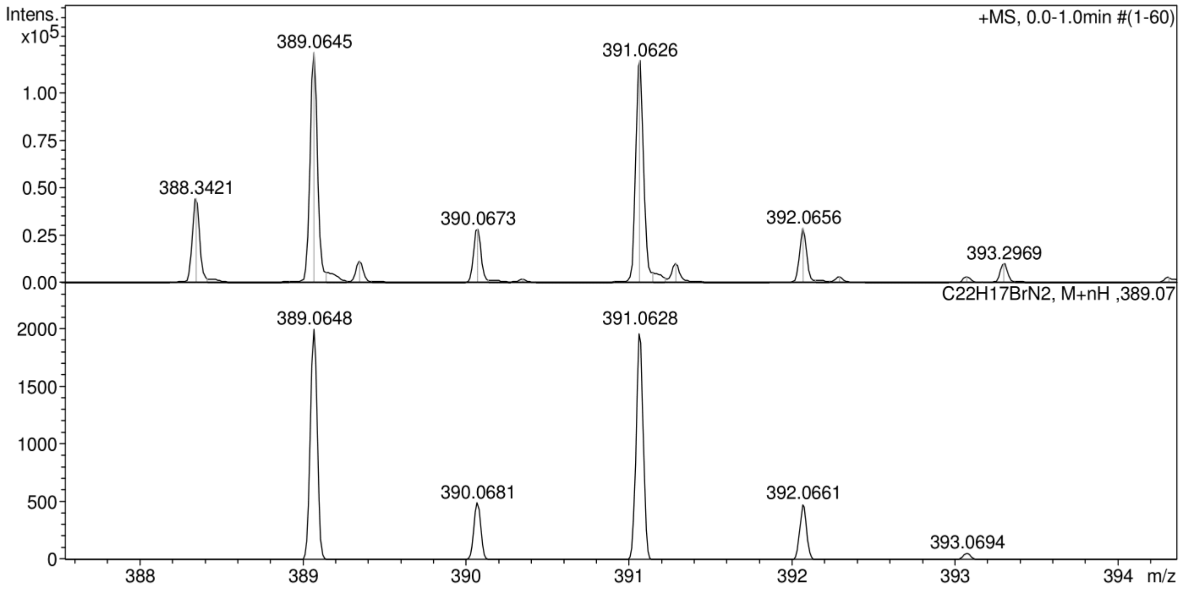

HRMS of 3i

Display Report

Analysis Info

Analysis Name	D:\Data\Chizhov\Terentiev\Wil\sb-544_&clblow.d	Acquisition Date	31.10.2022 17:10:30
Method	tune_low.m	Operator	BDAL@DE
Sample Name	/TERN SB-544	Instrument / Ser#	micrOTOF 10248
Comment	CH3CN 100 %, dil. 2000, calibrant added		

Acquisition Parameter

Source Type	ESI	Ion Polarity	Positive	Set Nebulizer	0.4 Bar
Focus	Not active			Set Dry Heater	180 °C
Scan Begin	50 m/z	Set Capillary	4500 V	Set Dry Gas	4.0 l/min
Scan End	3000 m/z	Set End Plate Offset	-500 V	Set Divert Valve	Waste

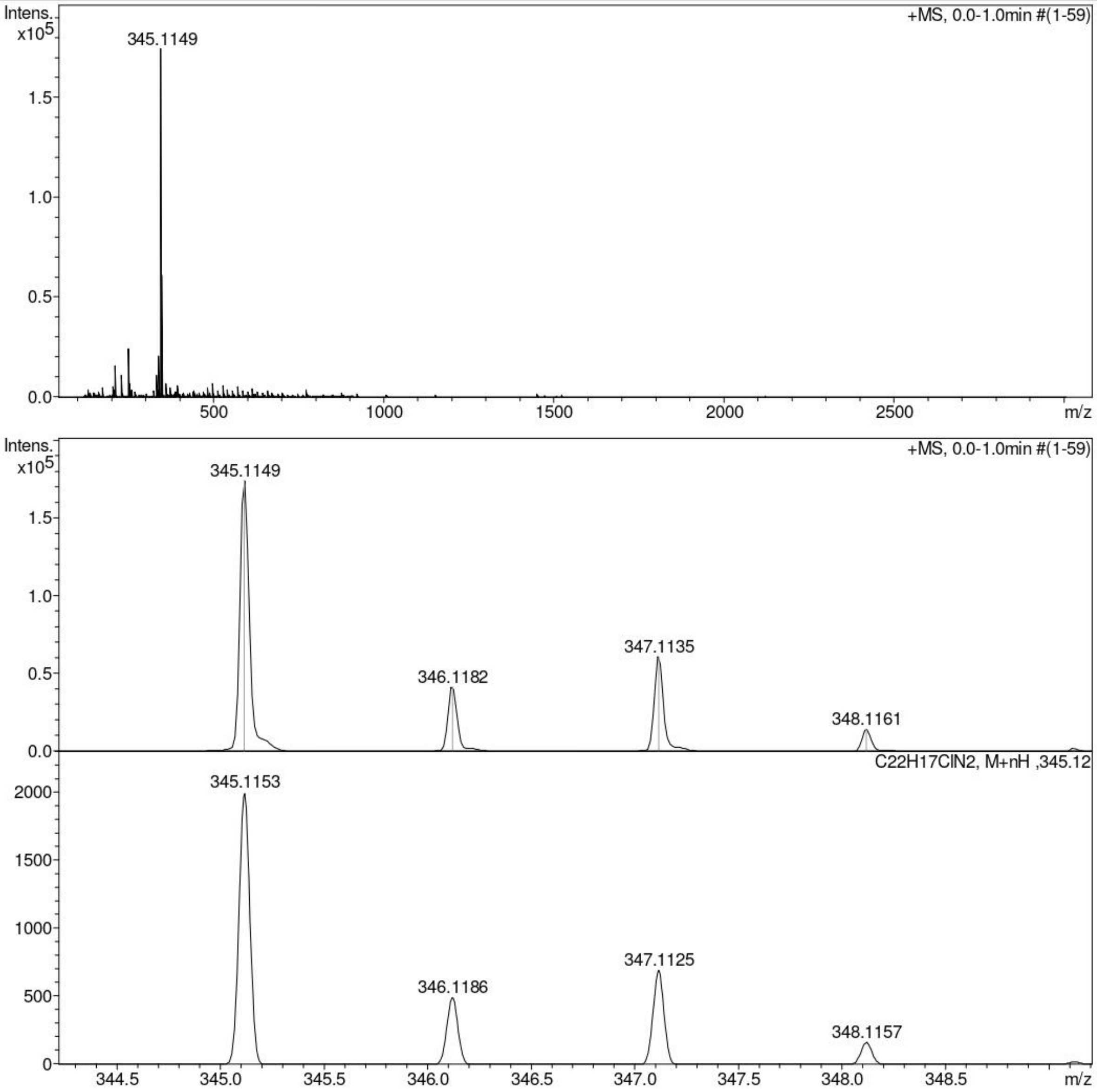

Bruker Compass DataAnalysis 4.0 printed: 31.10.2022 17:18:18 Page 1 of 1

HRMS of 3j

Display Report

Analysis Info

Analysis Name	D:\Data\Chizhov\Terentiev\Wil\sg-514_&clblow.d	Acquisition Date	11.04.2022 12:16:49
Method	tune_low.m	Operator	BDAL@DE
Sample Name	/TERN SG-514	Instrument / Ser#	micrOTOF 10248
Comment	CH3CN 100 %, dil. 2000, calibrant added		

Acquisition Parameter

Source Type	ESI	Ion Polarity	Positive	Set Nebulizer	0.4 Bar
Focus	Not active			Set Dry Heater	180 °C
Scan Begin	50 m/z	Set Capillary	4500 V	Set Dry Gas	4.0 l/min
Scan End	3000 m/z	Set End Plate Offset	-500 V	Set Divert Valve	Waste

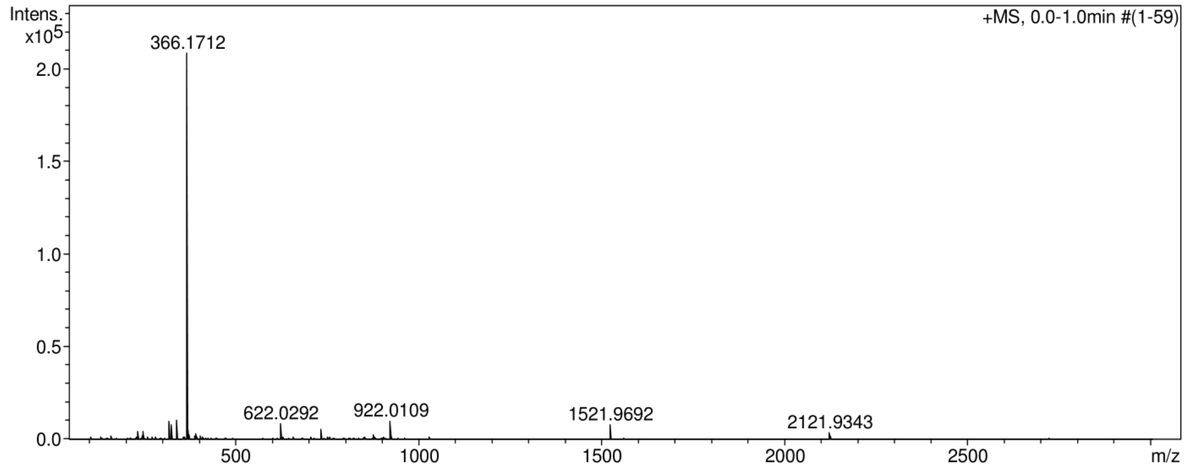

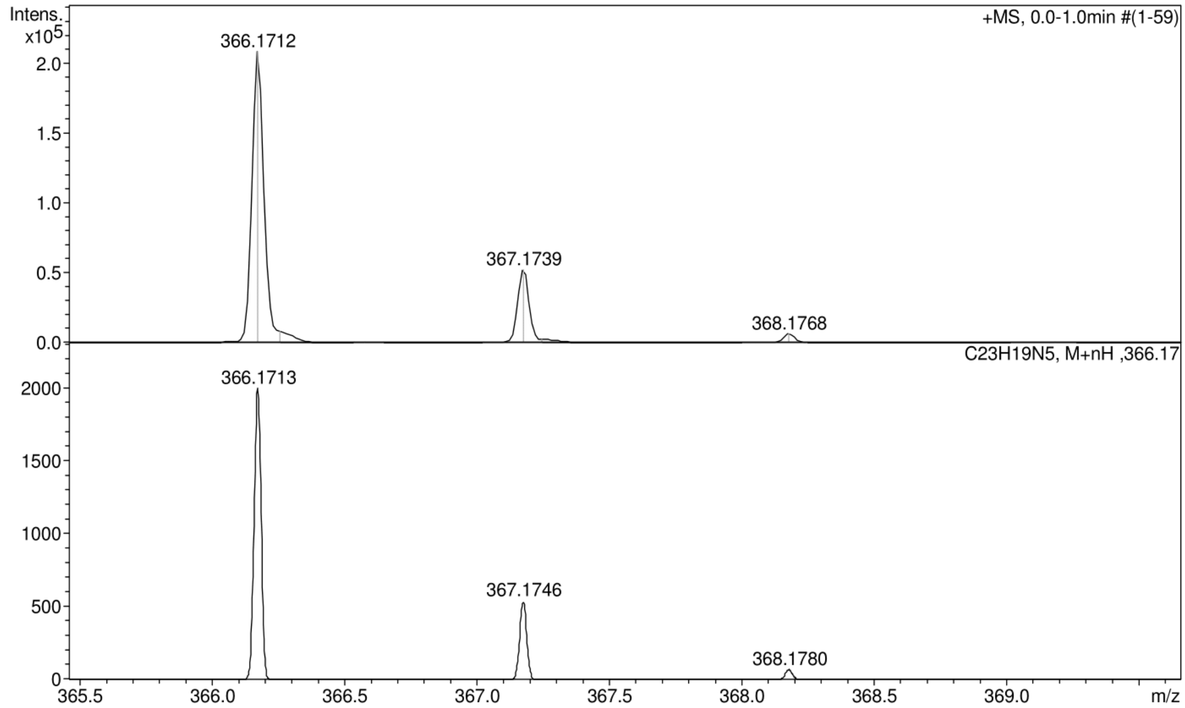

Bruker Compass DataAnalysis 4.0 printed: 11.04.2022 12:23:16 Page 1 of 1

HRMS of 3l

Display Report

Analysis Info

Analysis Name	D:\Data\Chizhov\Terent'ev\Vil'\sb-539_&clblow.d	Acquisition Date	01.11.2022 13:14:17
Method	tune_low_1550.m	Operator	BDAL@DE
Sample Name	/TERN SB-539	Instrument / Ser#	maXis 43
Comment	CH3CN 100 %, dil. 2000, calibrant added		

Acquisition Parameter

Source Type	ESI	Ion Polarity	Positive	Set Nebulizer	0.4 Bar
Focus	Not active			Set Dry Heater	180 °C
Scan Begin	50 m/z	Set Capillary	4500 V	Set Dry Gas	4.0 l/min
Scan End	1550 m/z	Set End Plate Offset	-500 V	Set Divert Valve	Source

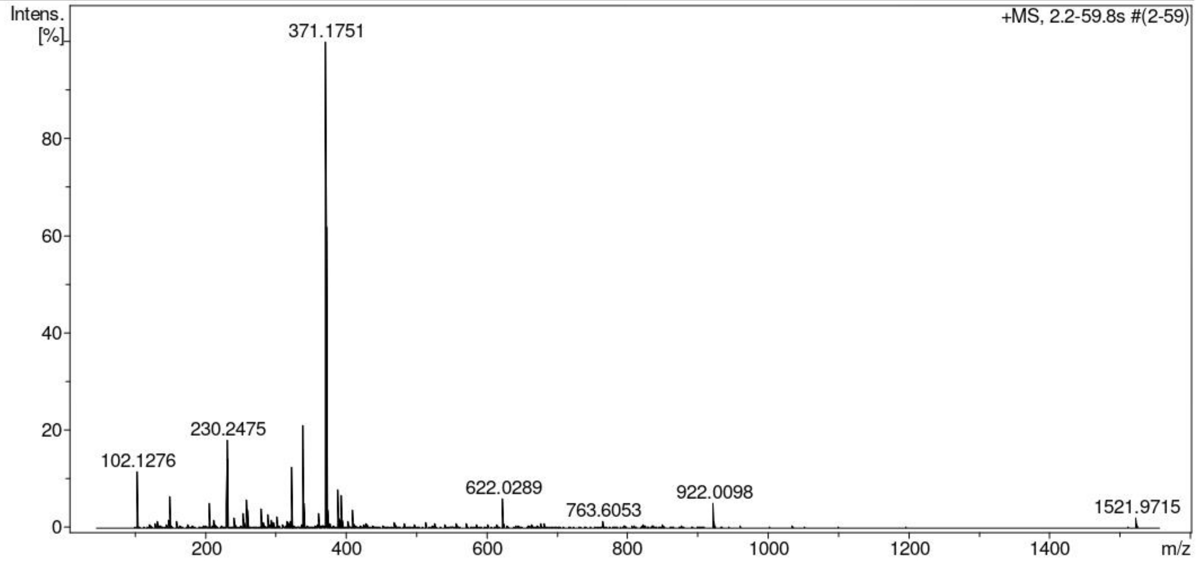

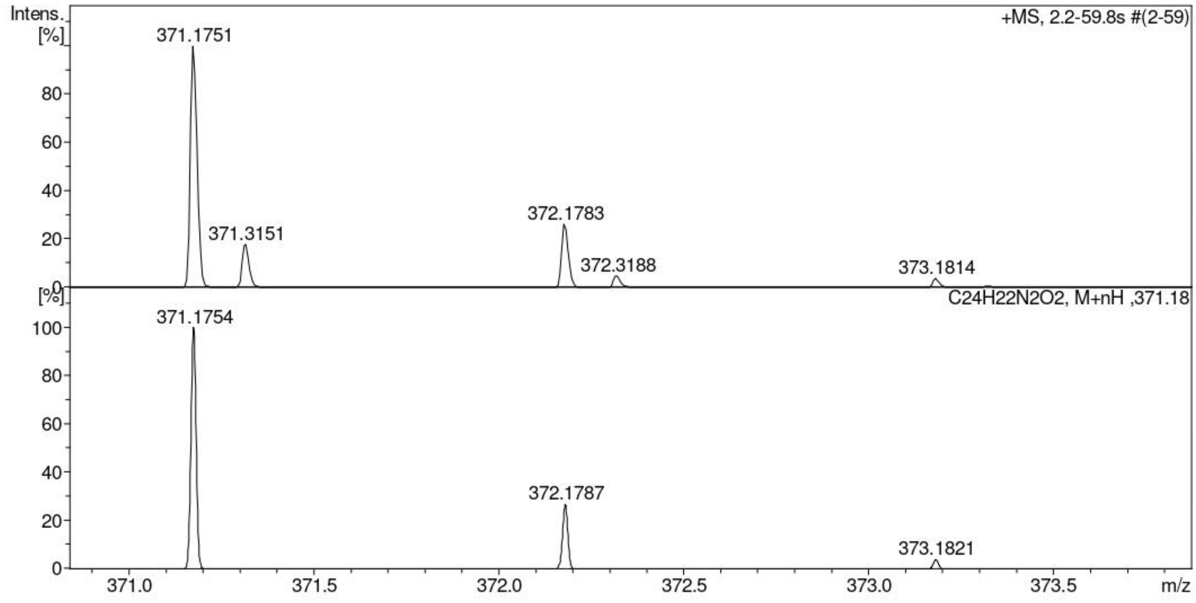

HRMS of 3m

Display Report

Analysis Info

Analysis Name	D:\Data\Chizhov\Terent'ev\Vil'\sb-545_&clblow.d	Acquisition Date	01.11.2022 13:18:24
Method	tune_low_1550.m	Operator	BDAL@DE
Sample Name	/TERN SB-545	Instrument / Ser#	maXis 43
Comment	CH3CN 100 %, dil. 2000, calibrant added		

Acquisition Parameter

Source Type	ESI	Ion Polarity	Positive	Set Nebulizer	0.4 Bar
Focus	Not active			Set Dry Heater	180 °C
Scan Begin	50 m/z	Set Capillary	4500 V	Set Dry Gas	4.0 l/min
Scan End	1550 m/z	Set End Plate Offset	-500 V	Set Divert Valve	Source

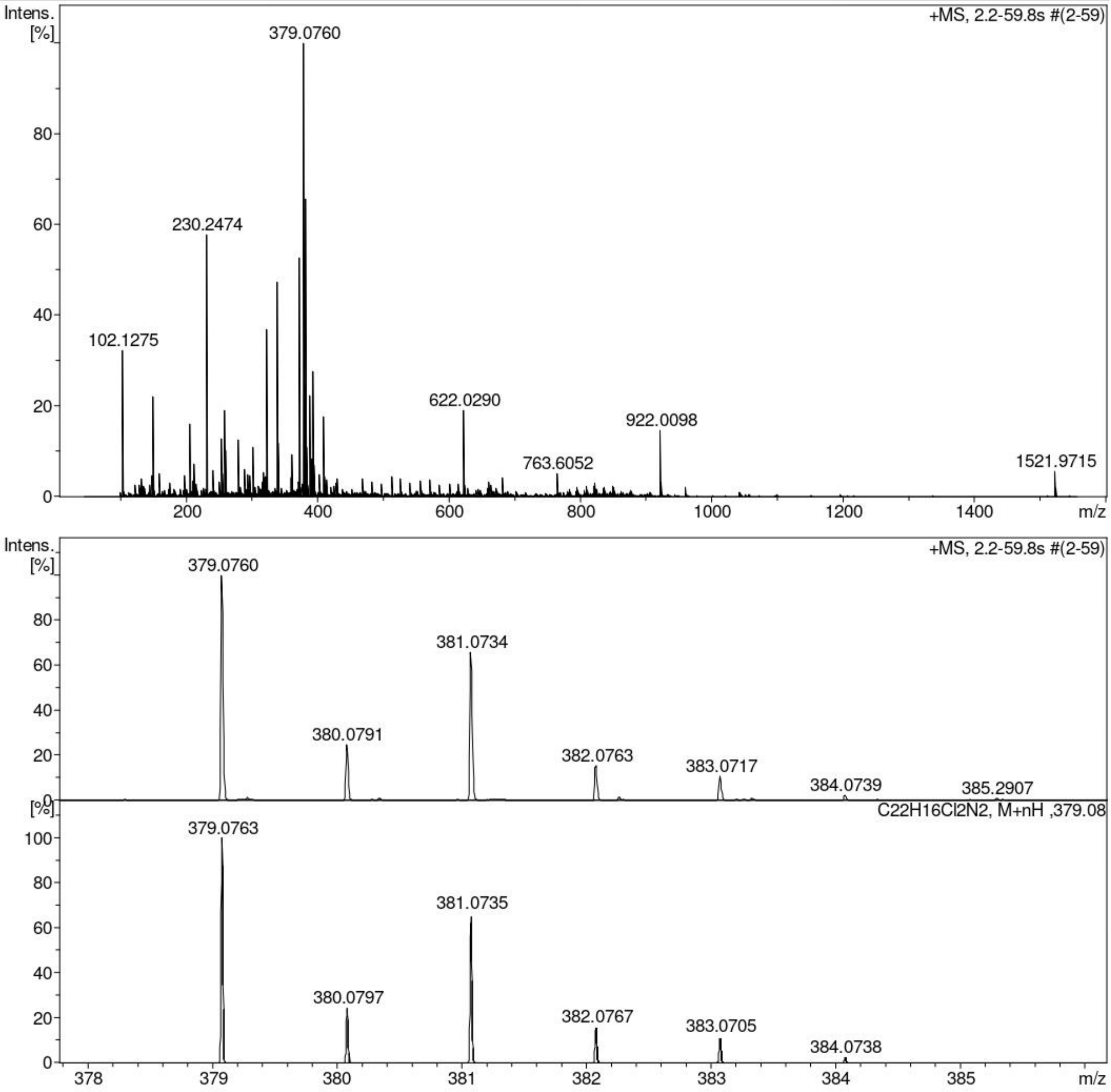

HRMS of 3n

Display Report

Analysis Info

Analysis Name	D:\Data\Chizhov\Terent'ev\Vil'\sb-536-2_&clblow.d	Acquisition Date	01.11.2022 13:03:24
Method	tune_low_1550.m	Operator	BDAL@DE
Sample Name	/TERN SB-536-2	Instrument / Ser#	maXis 43
Comment	CH3CN 100 %, dil. 2000, calibrant added		

Acquisition Parameter

Source Type	ESI	Ion Polarity	Positive	Set Nebulizer	0.4 Bar
Focus	Not active			Set Dry Heater	180 °C
Scan Begin	50 m/z	Set Capillary	4500 V	Set Dry Gas	4.0 l/min
Scan End	1550 m/z	Set End Plate Offset	-500 V	Set Divert Valve	Source

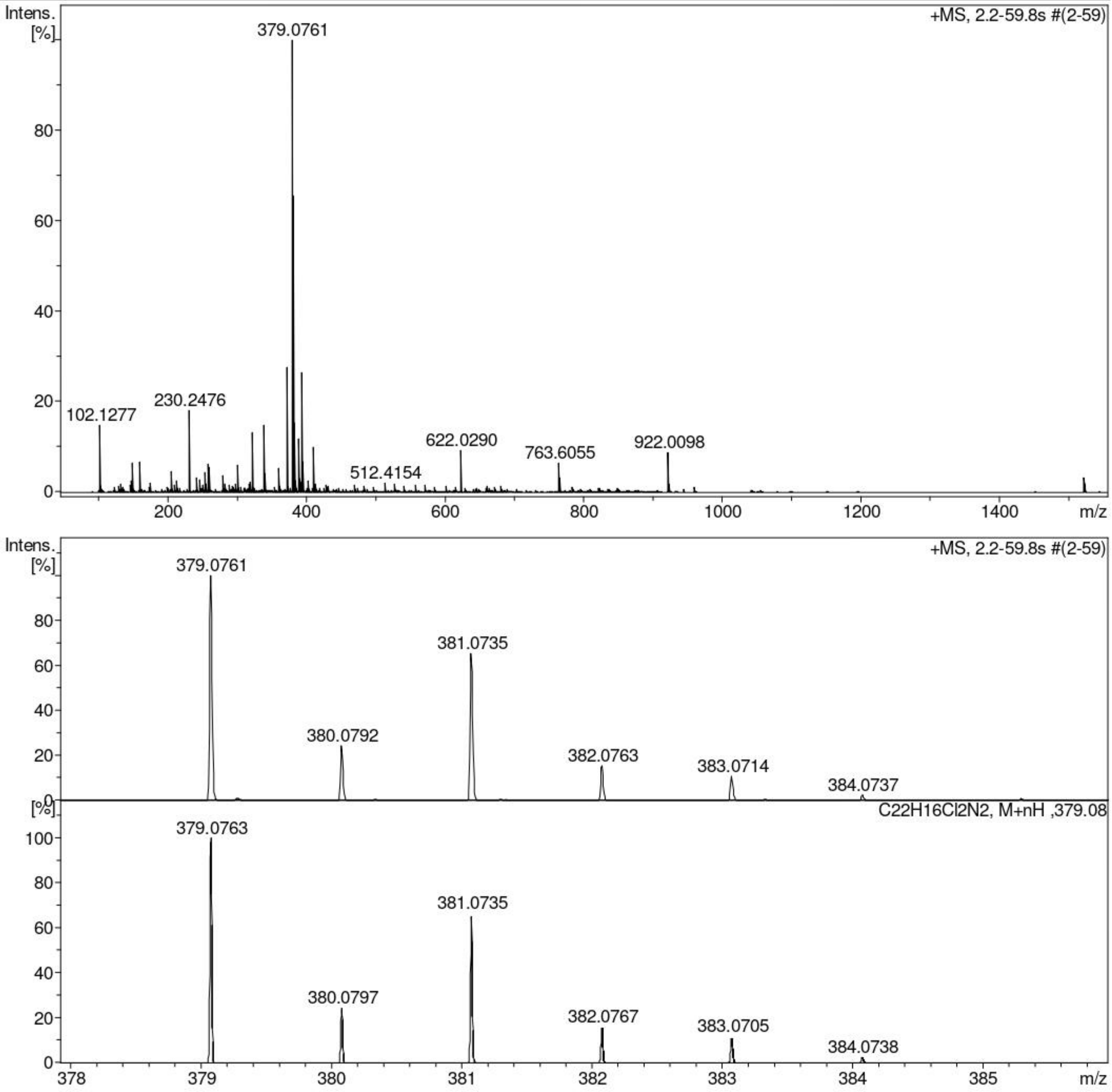

Bruker Compass DataAnalysis 4.0 printed: 01.11.2022 13:45:17 Page 1 of 1

HRMS of 3o

Display Report

Analysis Info

Analysis Name	D:\Data\Chizhov\Terentiev\Wil\sb-543_&clblow.d	Acquisition Date	31.10.2022 17:05:10
Method	tune_low.m	Operator	BDAL@DE
Sample Name	/TERN SB-543	Instrument / Ser#	micrOTOF 10248
Comment	CH3CN 100 %, dil. 2000, calibrant added		

Acquisition Parameter

Source Type	ESI	Ion Polarity	Positive	Set Nebulizer	0.4 Bar
Focus	Not active			Set Dry Heater	180 °C
Scan Begin	50 m/z	Set Capillary	4500 V	Set Dry Gas	4.0 l/min
Scan End	3000 m/z	Set End Plate Offset	-500 V	Set Divert Valve	Waste

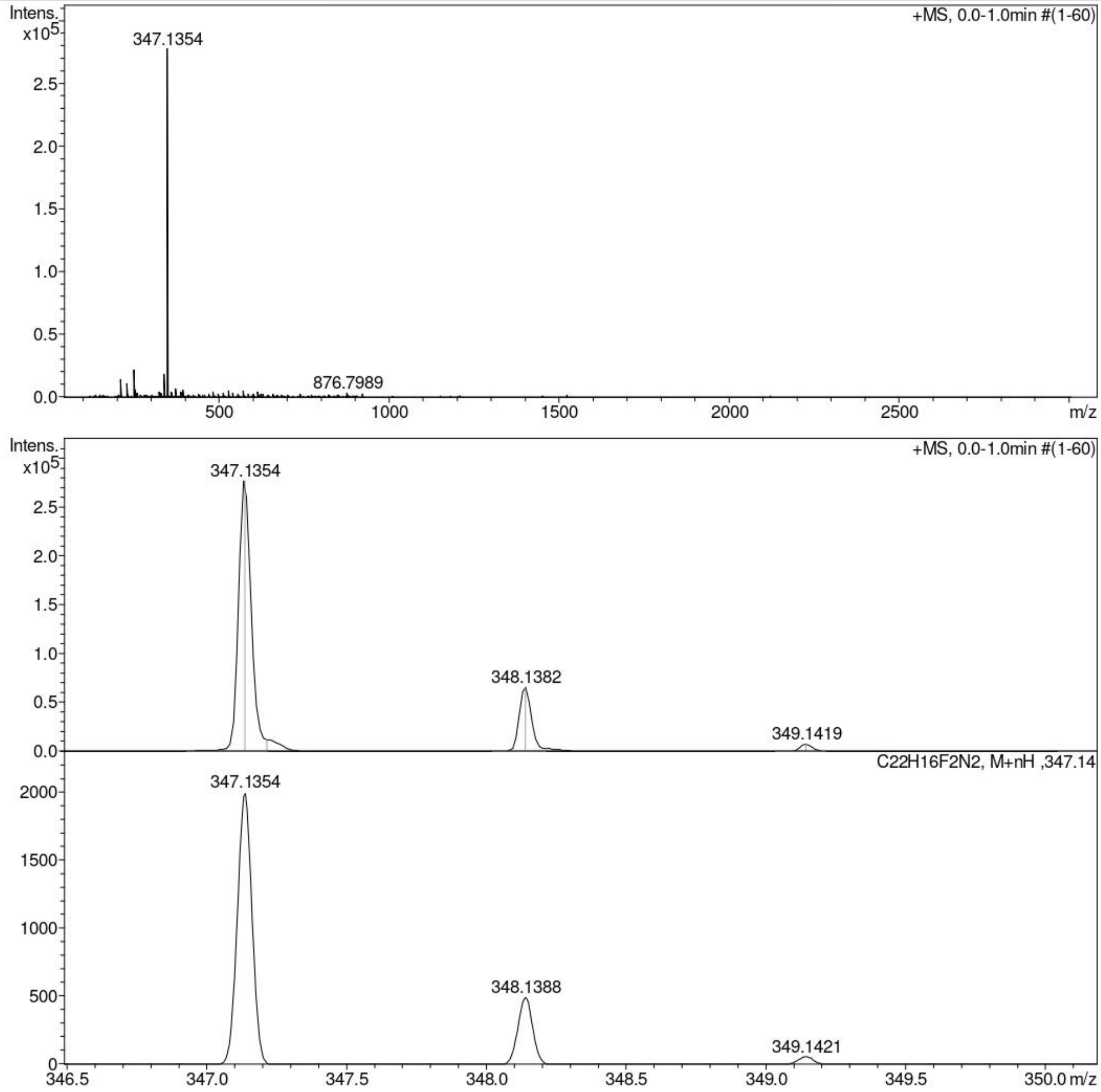

Bruker Compass DataAnalysis 4.0 printed: 31.10.2022 17:15:23 Page 1 of 1

HRMS of 3p

Display Report

Analysis Info

Analysis Name	D:\Data\Chizhov\Terentiev\Wil\sg-540_&clblow.d	Acquisition Date	20.06.2022 16:19:31
Method	tune_low.m	Operator	BDAL@DE
Sample Name	/TERN SG-540	Instrument / Ser#	micrOTOF 10248
Comment	CH3CN 100 %, dil. 2000, calibrant added		

Acquisition Parameter

Source Type	ESI	Ion Polarity	Positive	Set Nebulizer	0.4 Bar
Focus	Not active			Set Dry Heater	180 °C
Scan Begin	50 m/z	Set Capillary	4500 V	Set Dry Gas	4.0 l/min
Scan End	3000 m/z	Set End Plate Offset	-500 V	Set Divert Valve	Waste

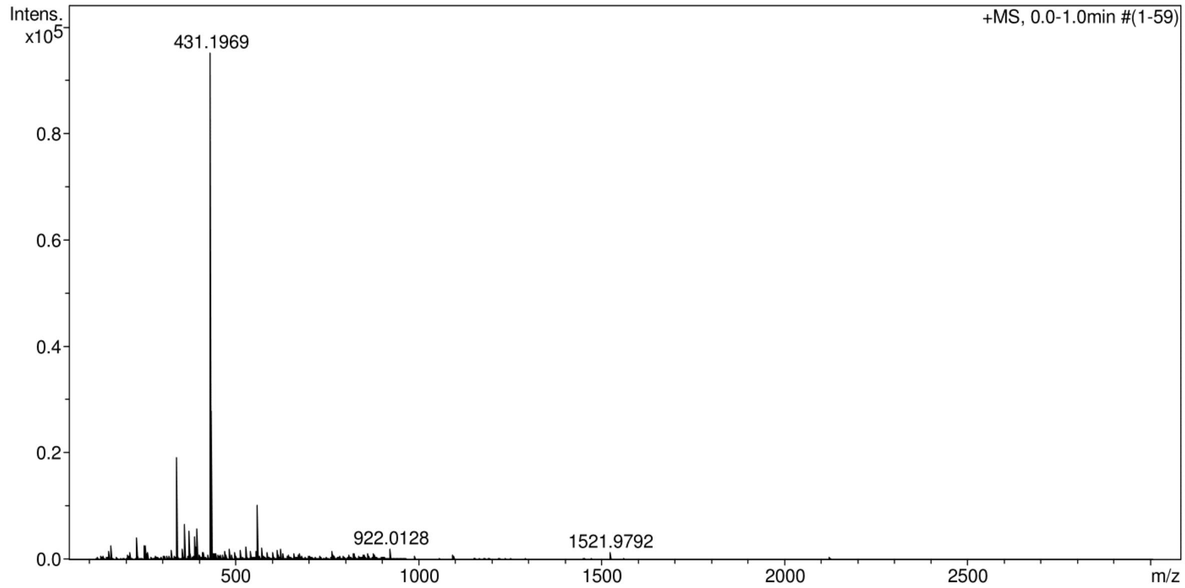

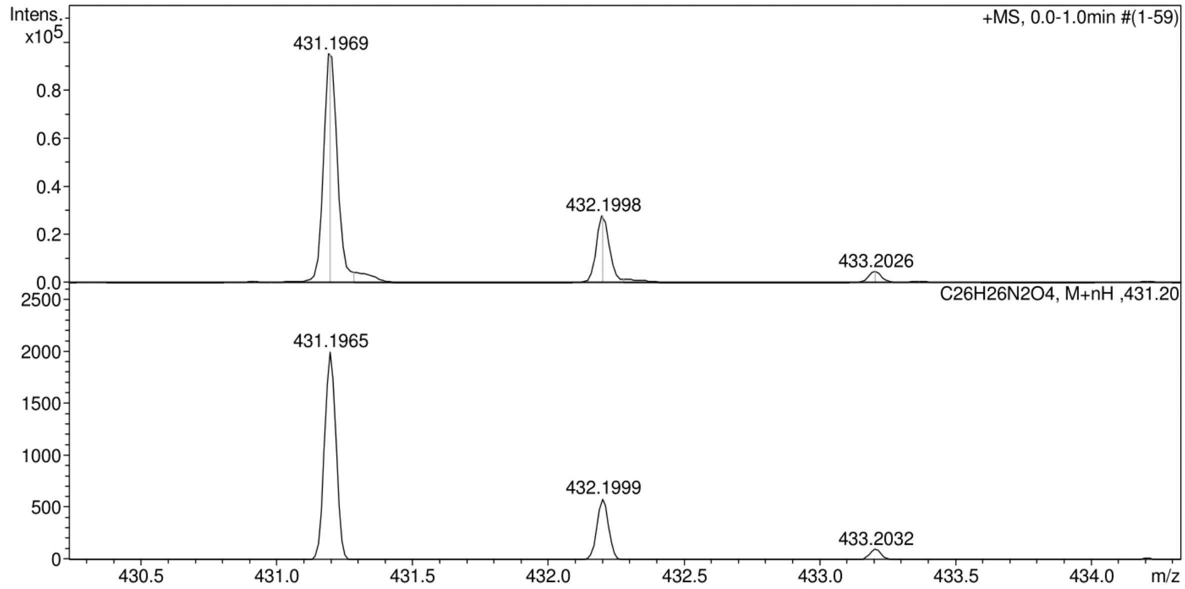

Bruker Compass DataAnalysis 4.0 printed: 20.06.2022 16:41:11 Page 1 of 1

HRMS of 3q

Display Report

Analysis Info

Analysis Name	D:\Data\Chizhov\Terent'ev\Vil'\sb-538_&clblow.d	Acquisition Date	01.11.2022 13:09:02
Method	tune_low_1550.m	Operator	BDAL@DE
Sample Name	/TERN SB-538	Instrument / Ser#	maXis 43
Comment	CH3CN 100 %, dil. 20000, calibrant added		

Acquisition Parameter

Source Type	ESI	Ion Polarity	Positive	Set Nebulizer	0.4 Bar
Focus	Not active			Set Dry Heater	180 °C
Scan Begin	50 m/z	Set Capillary	4500 V	Set Dry Gas	4.0 l/min
Scan End	1550 m/z	Set End Plate Offset	-500 V	Set Divert Valve	Source

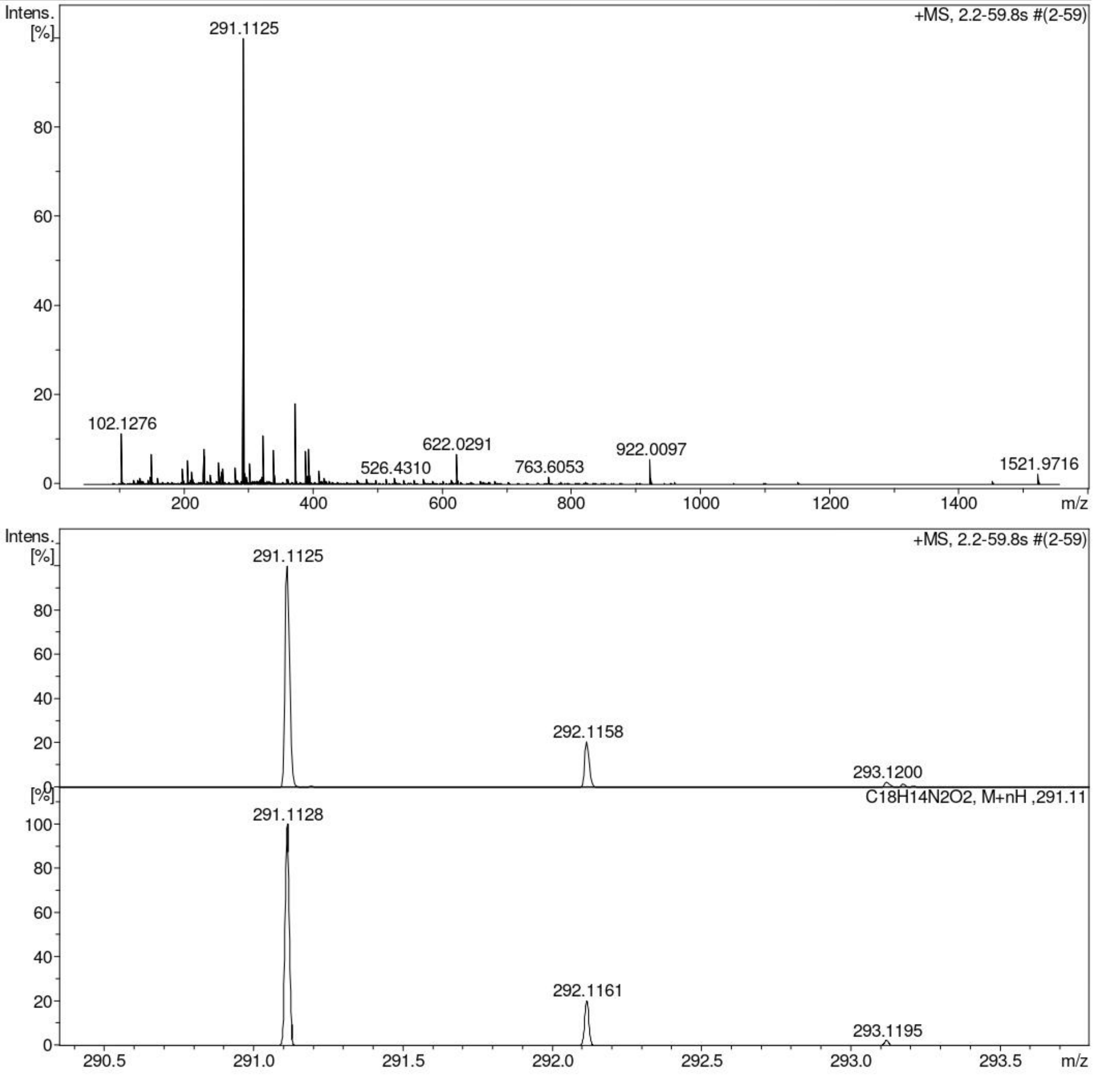

HRMS of 3r

Display Report

Analysis Info

Analysis Name	D:\Data\Chizhov\Terentiev\Wil\sg-549_&clblow.d	Acquisition Date	27.06.2022 15:49:24
Method	tune_low.m	Operator	BDAL@DE
Sample Name	/TERN SG-549	Instrument / Ser#	micrOTOF 10248
Comment	CH3CN 100 %, dil. 200, calibrant added		

Acquisition Parameter

Source Type	ESI	Ion Polarity	Positive	Set Nebulizer	0.4 Bar
Focus	Not active			Set Dry Heater	180 °C
Scan Begin	50 m/z	Set Capillary	4500 V	Set Dry Gas	4.0 l/min
Scan End	3000 m/z	Set End Plate Offset	-500 V	Set Divert Valve	Waste

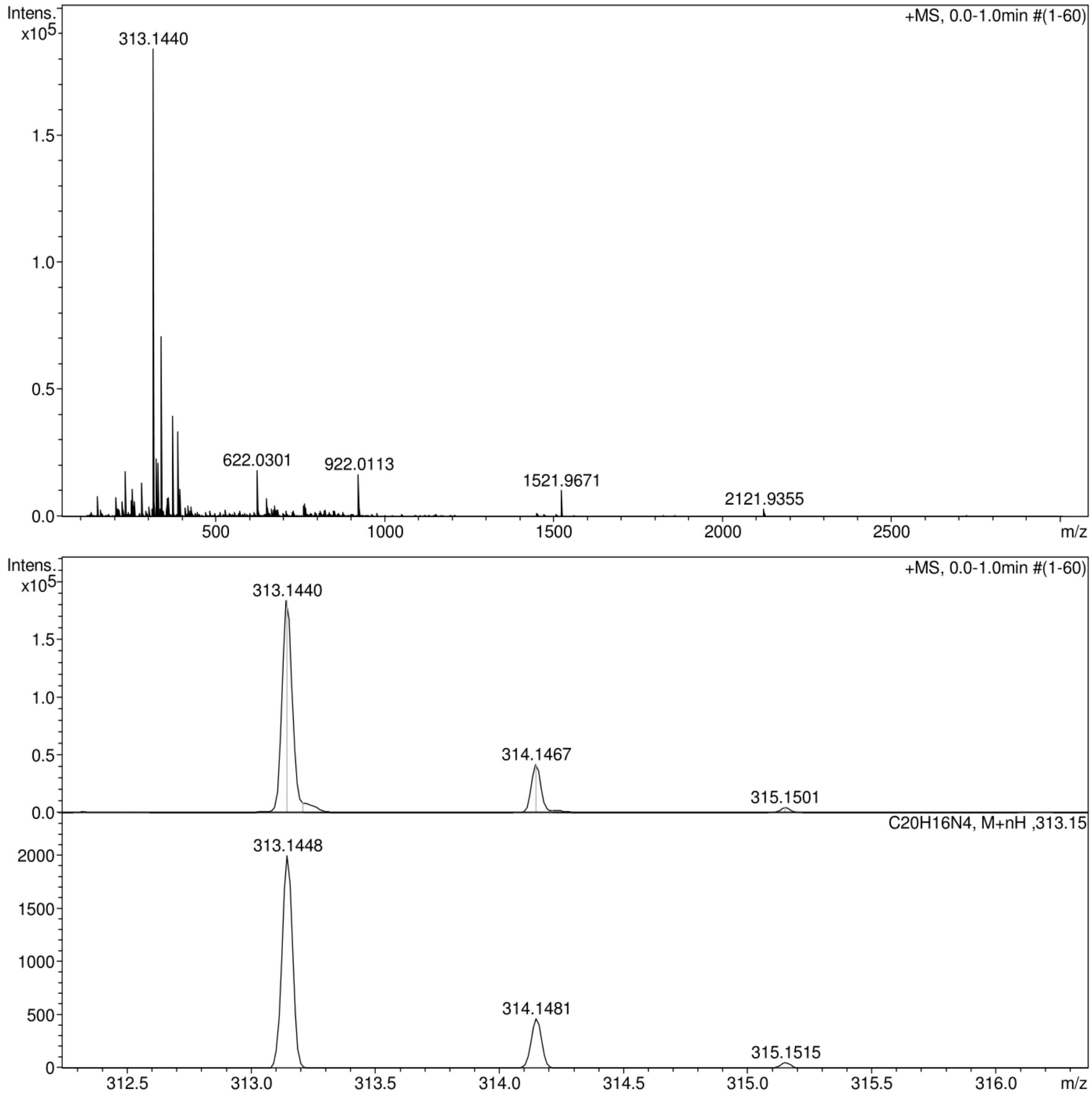

Bruker Compass DataAnalysis 4.0 printed: 27.06.2022 15:55:11 Page 1 of 1

IR spectra of synthesized compounds

IR of 3a

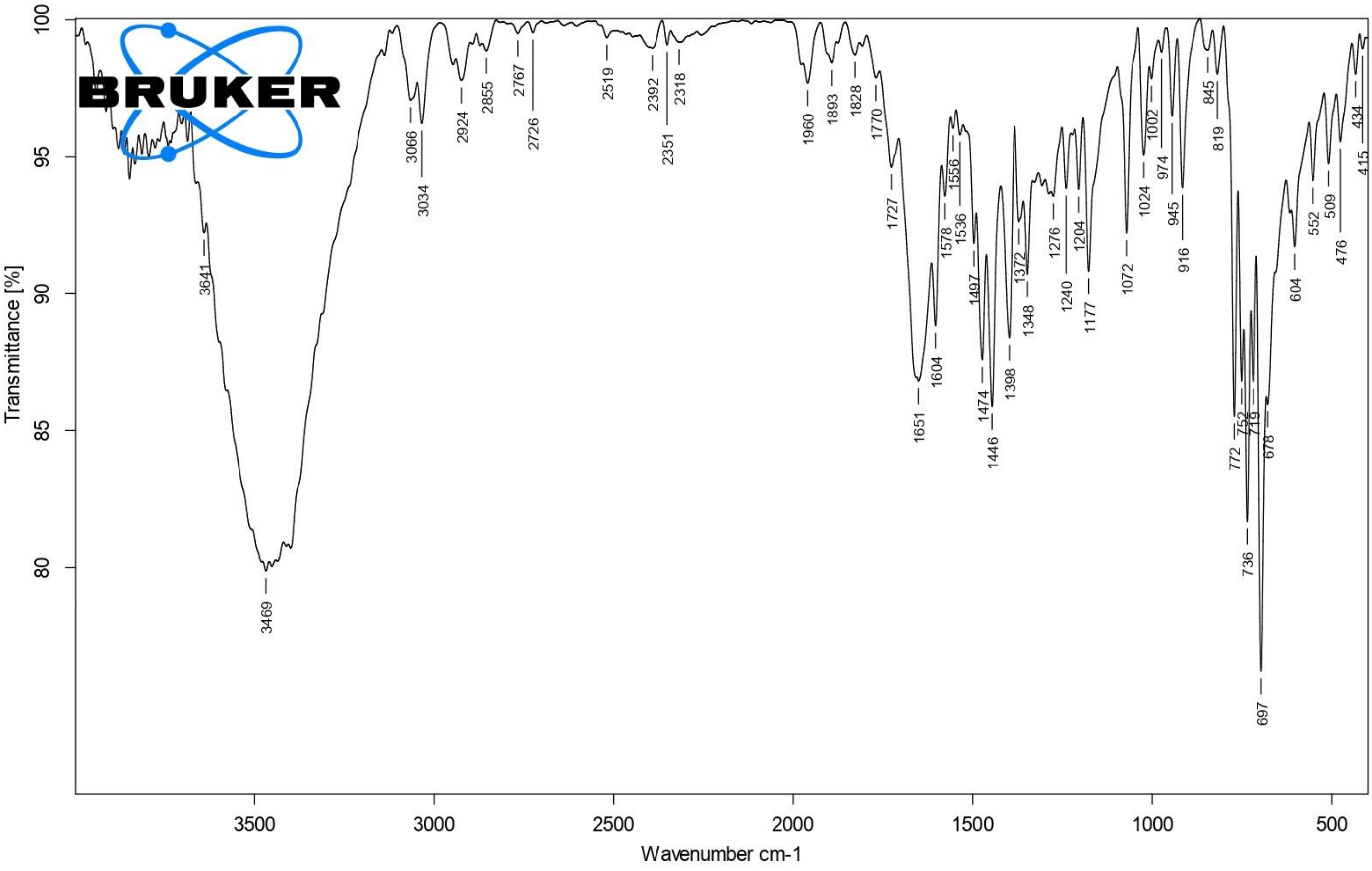

IR of 3b

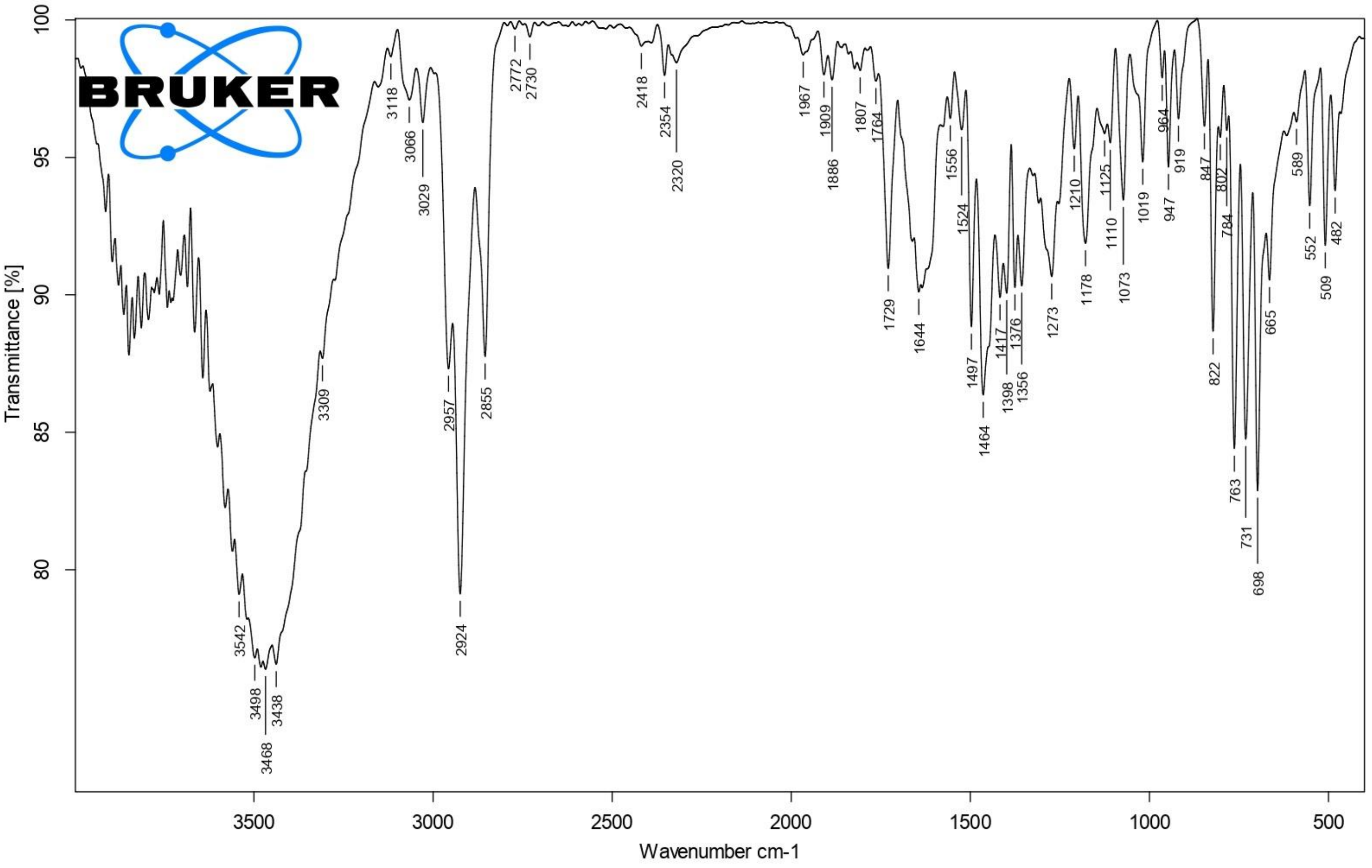

IR of 3c

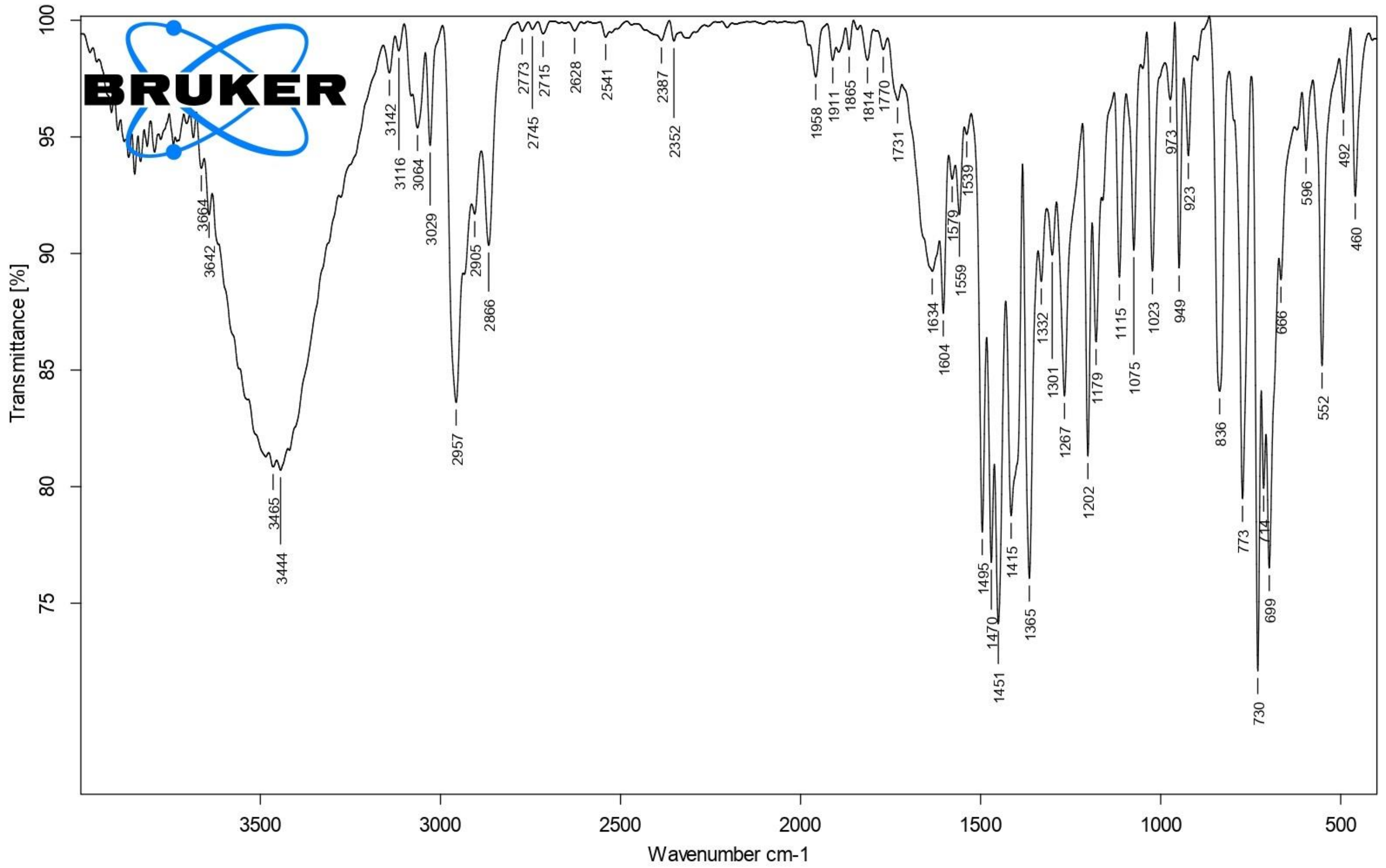

IR of 3d

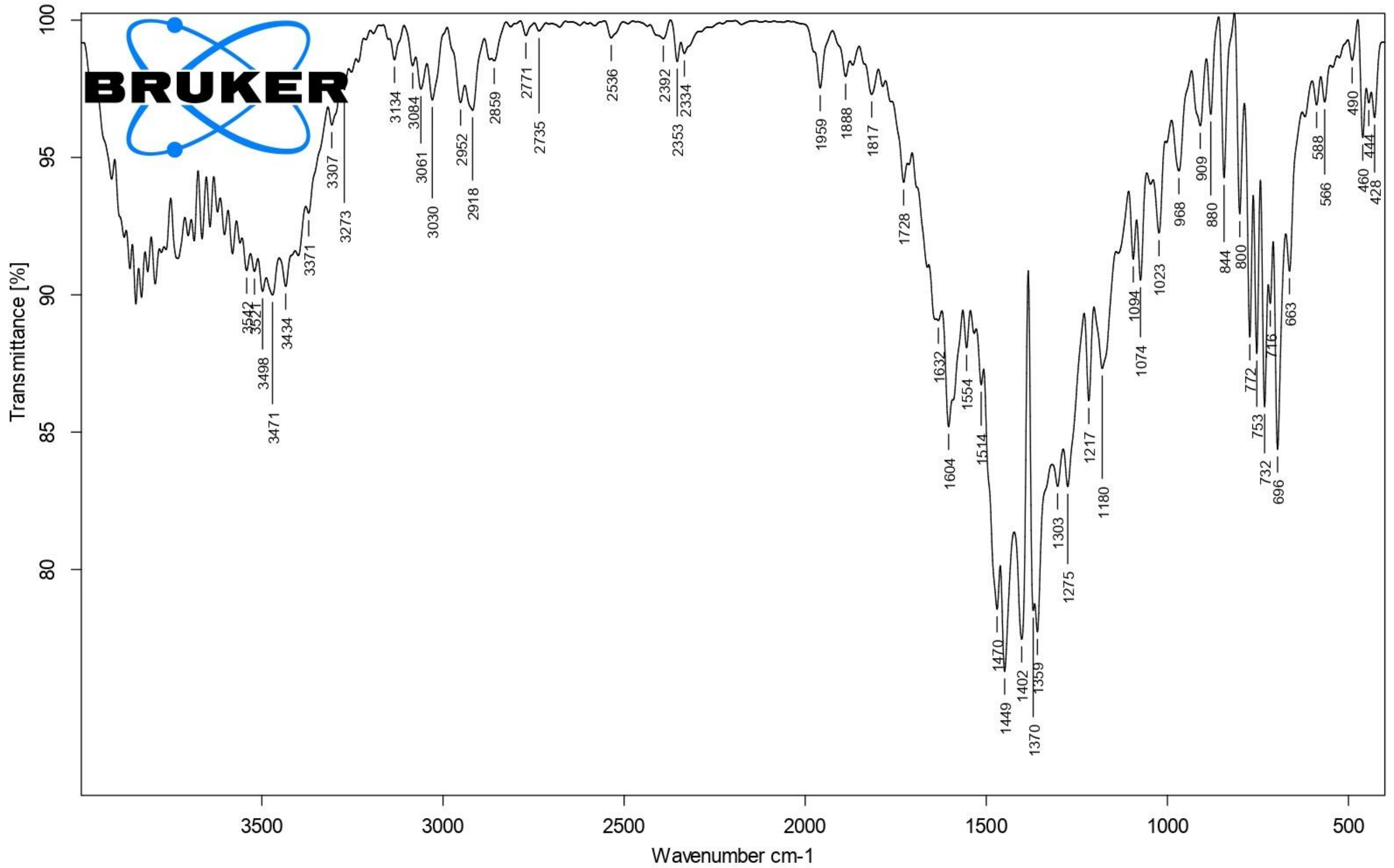

IR of 3e

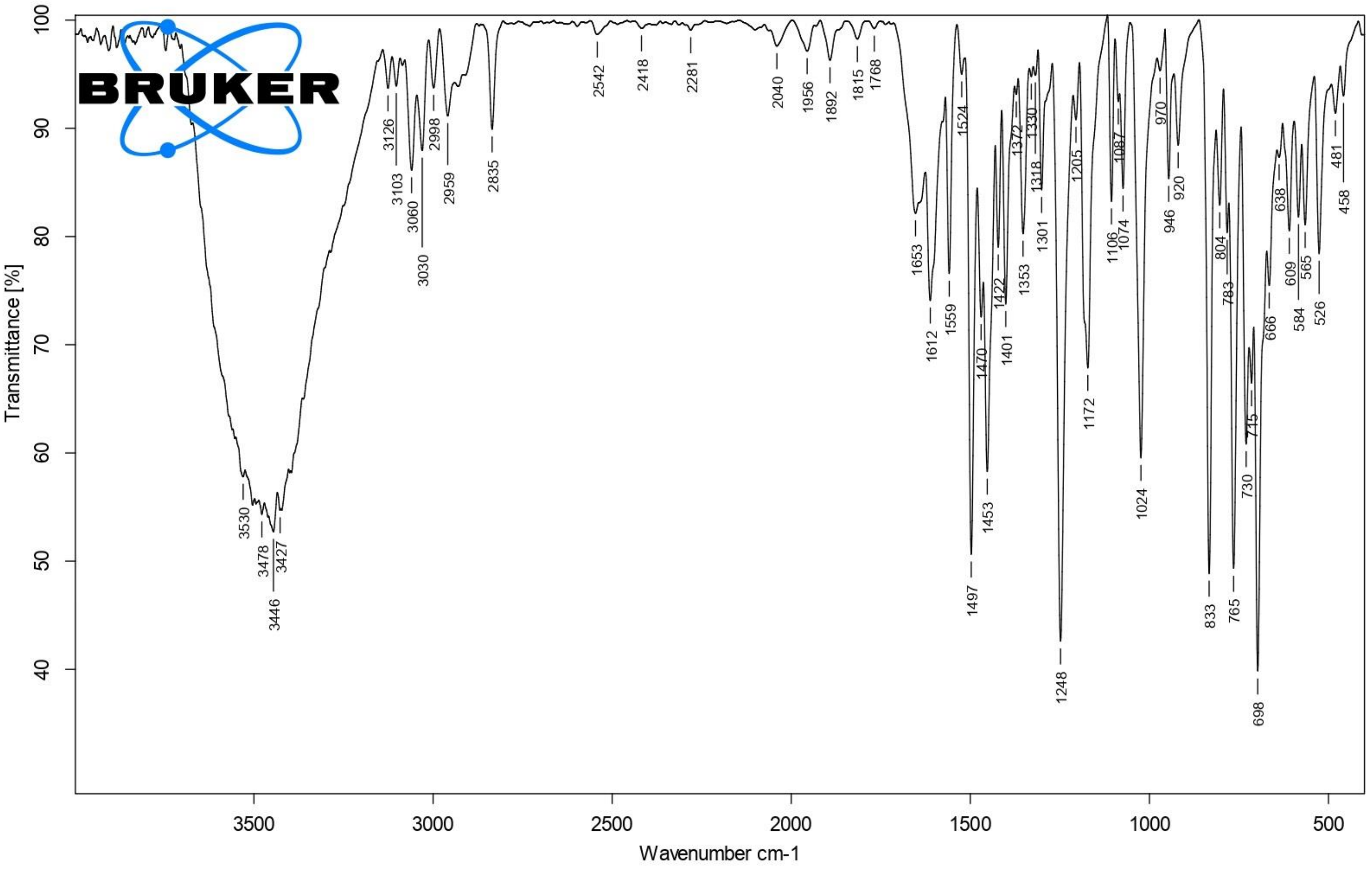

IR of 3f

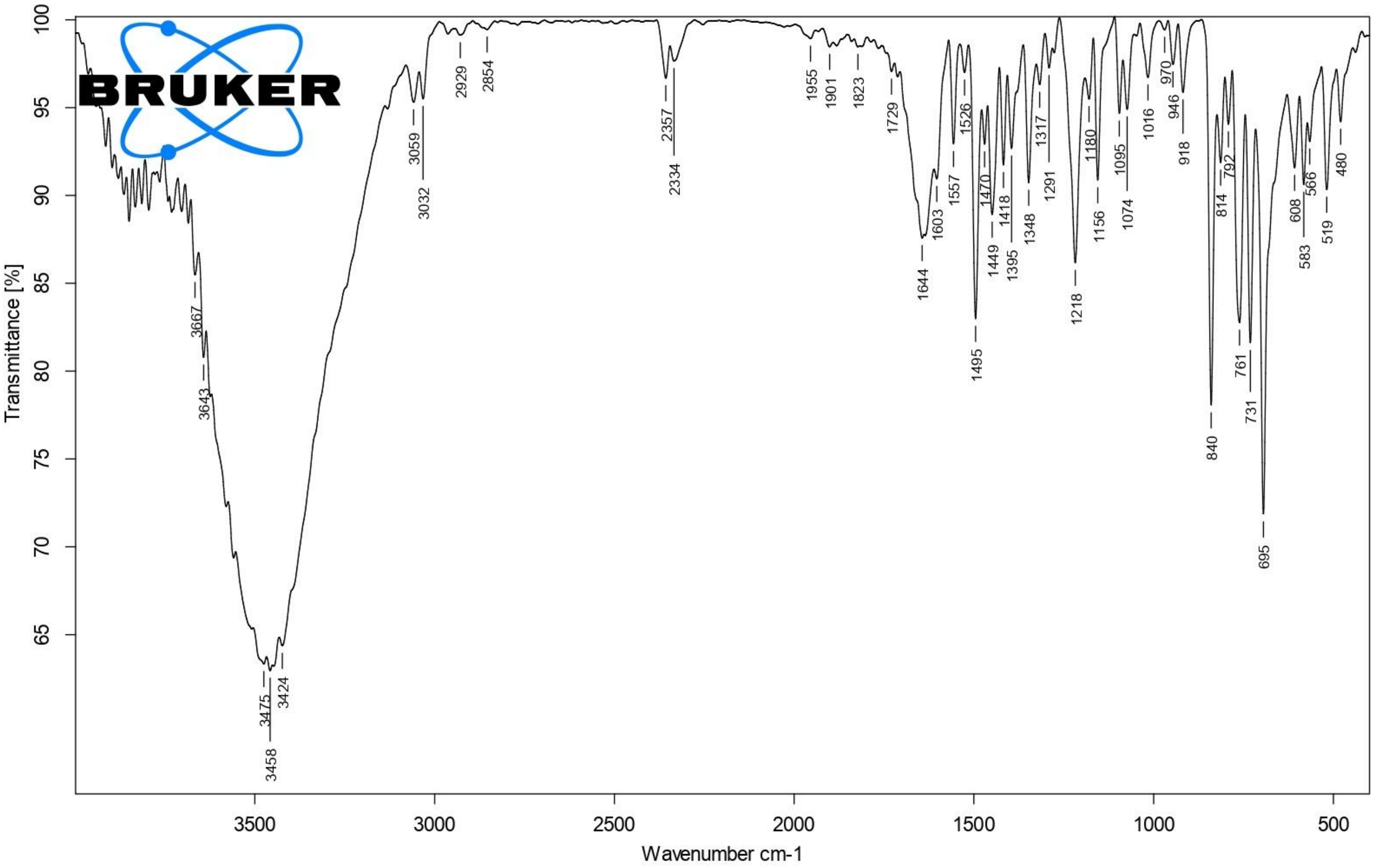

IR of 3g

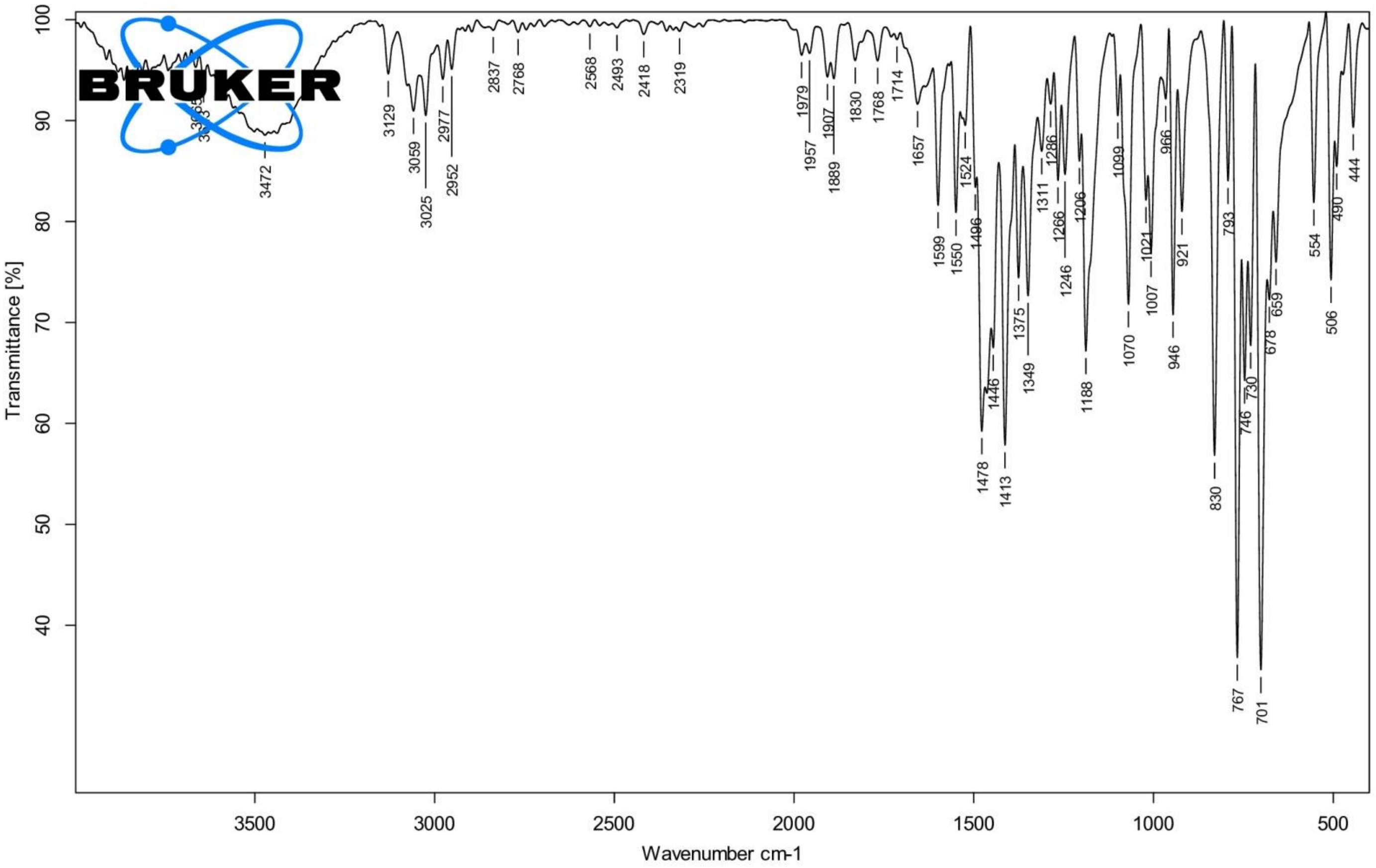

IR of 3h

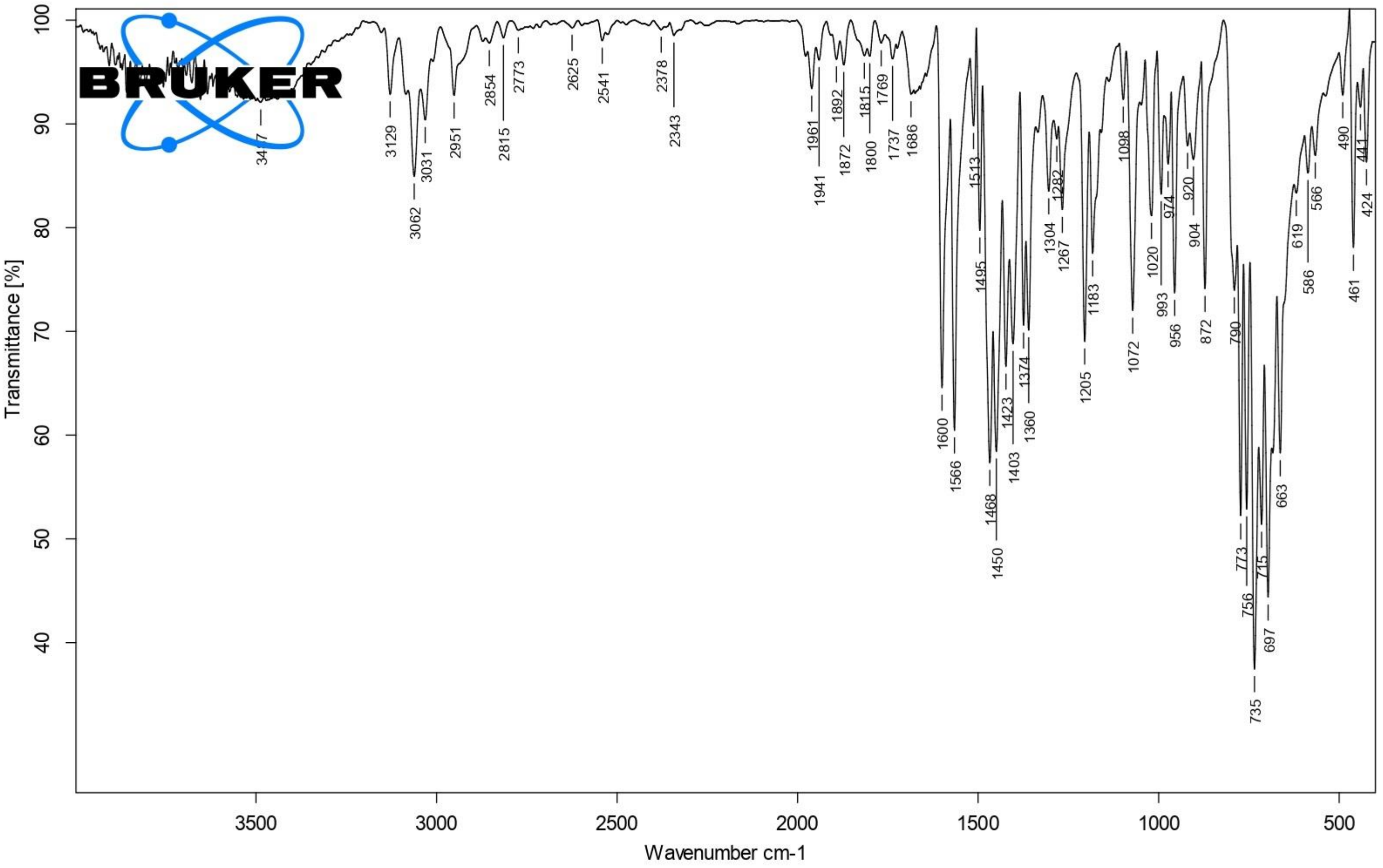

IR of 3i

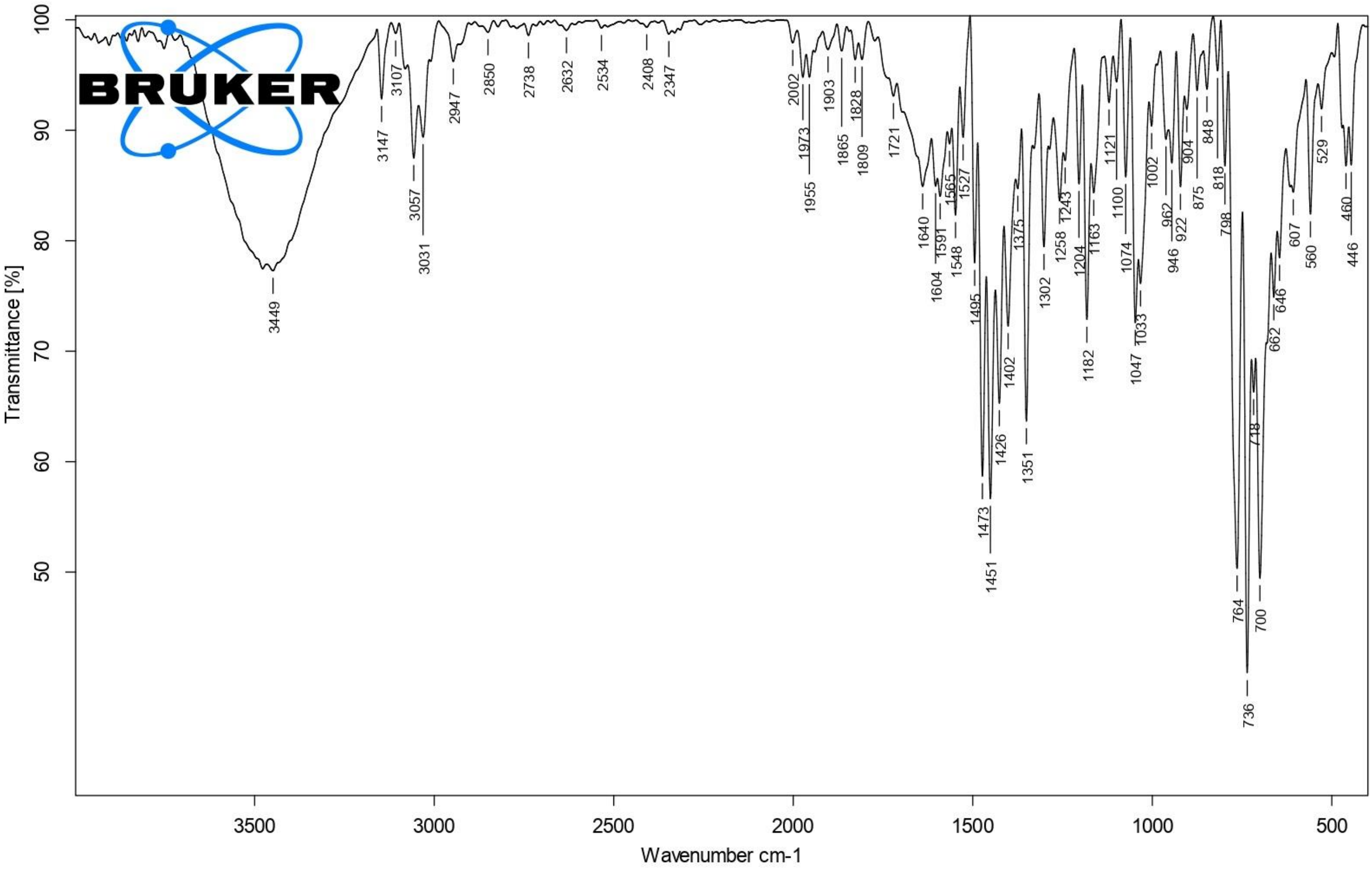

IR of 3j

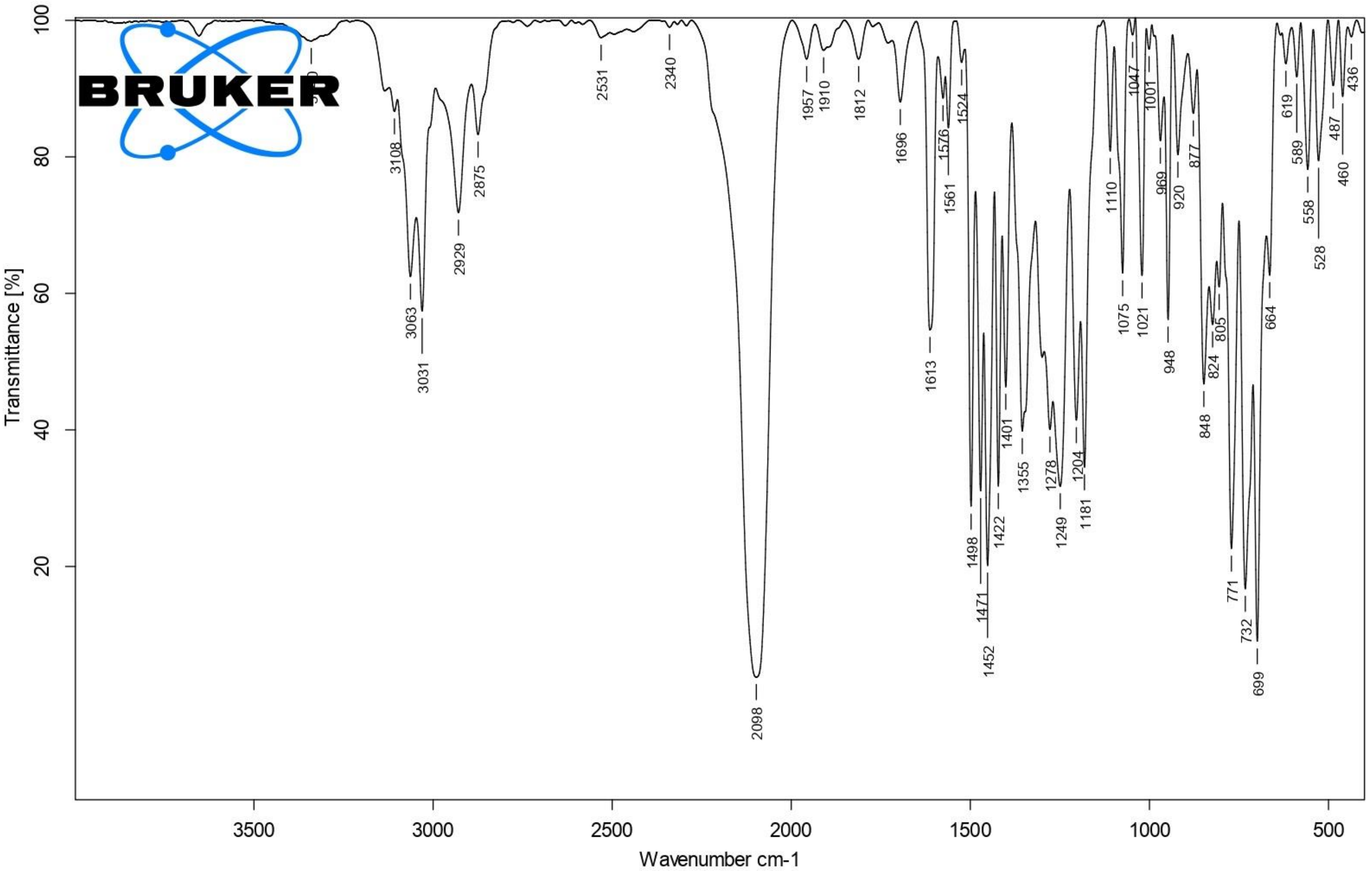

IR of 3l

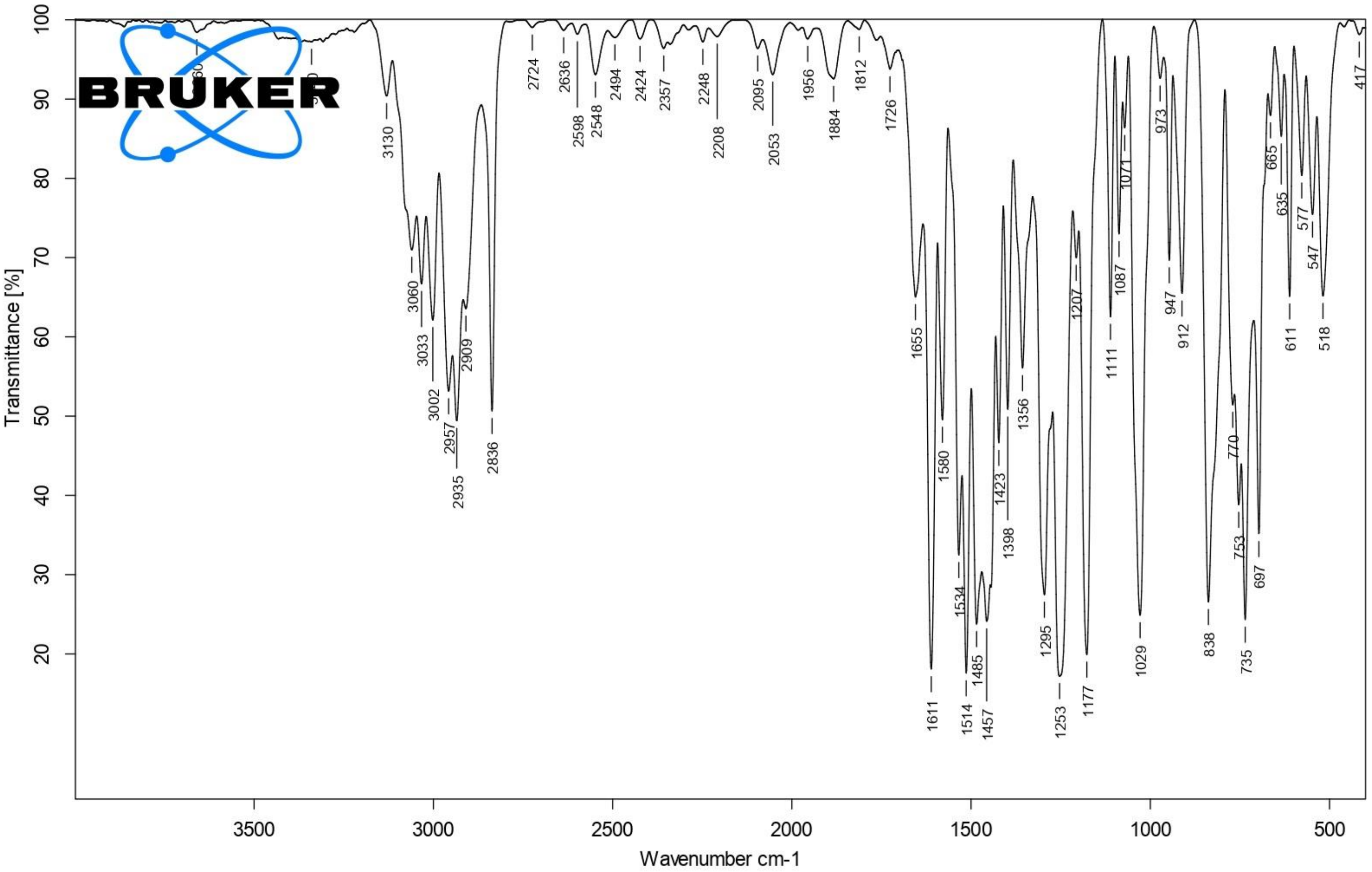

IR of 3m

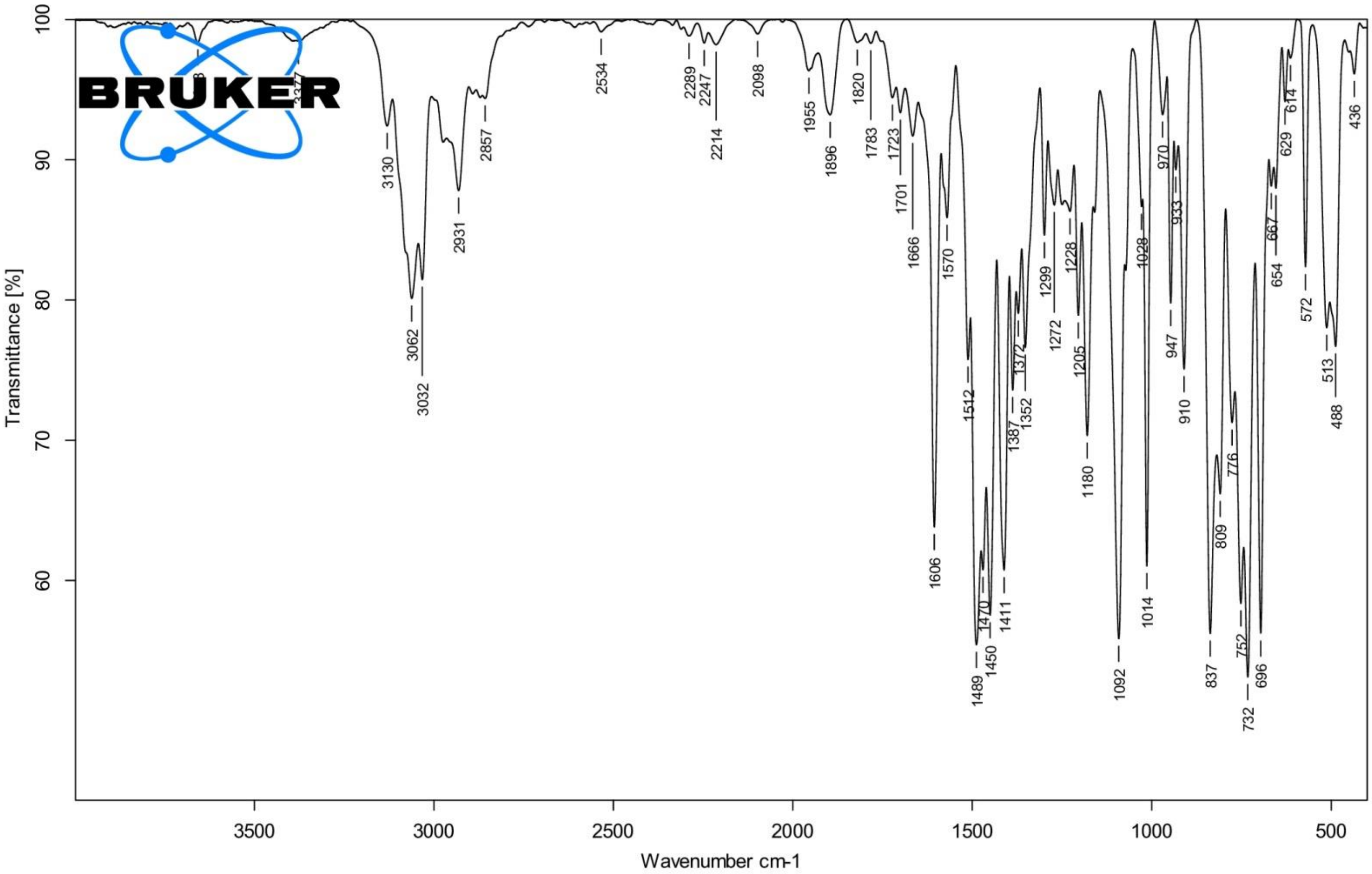

IR of 3n

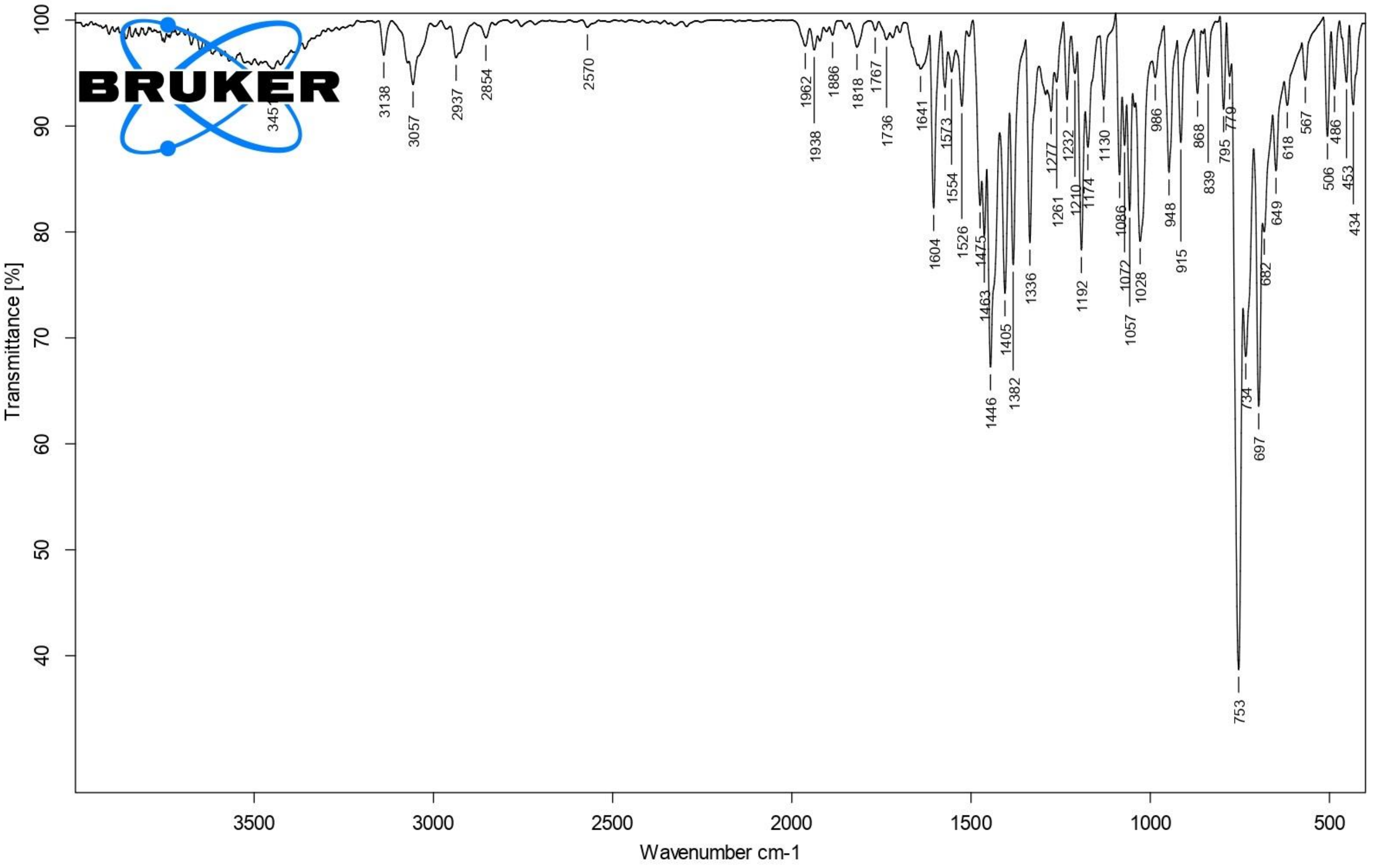

IR of 3o

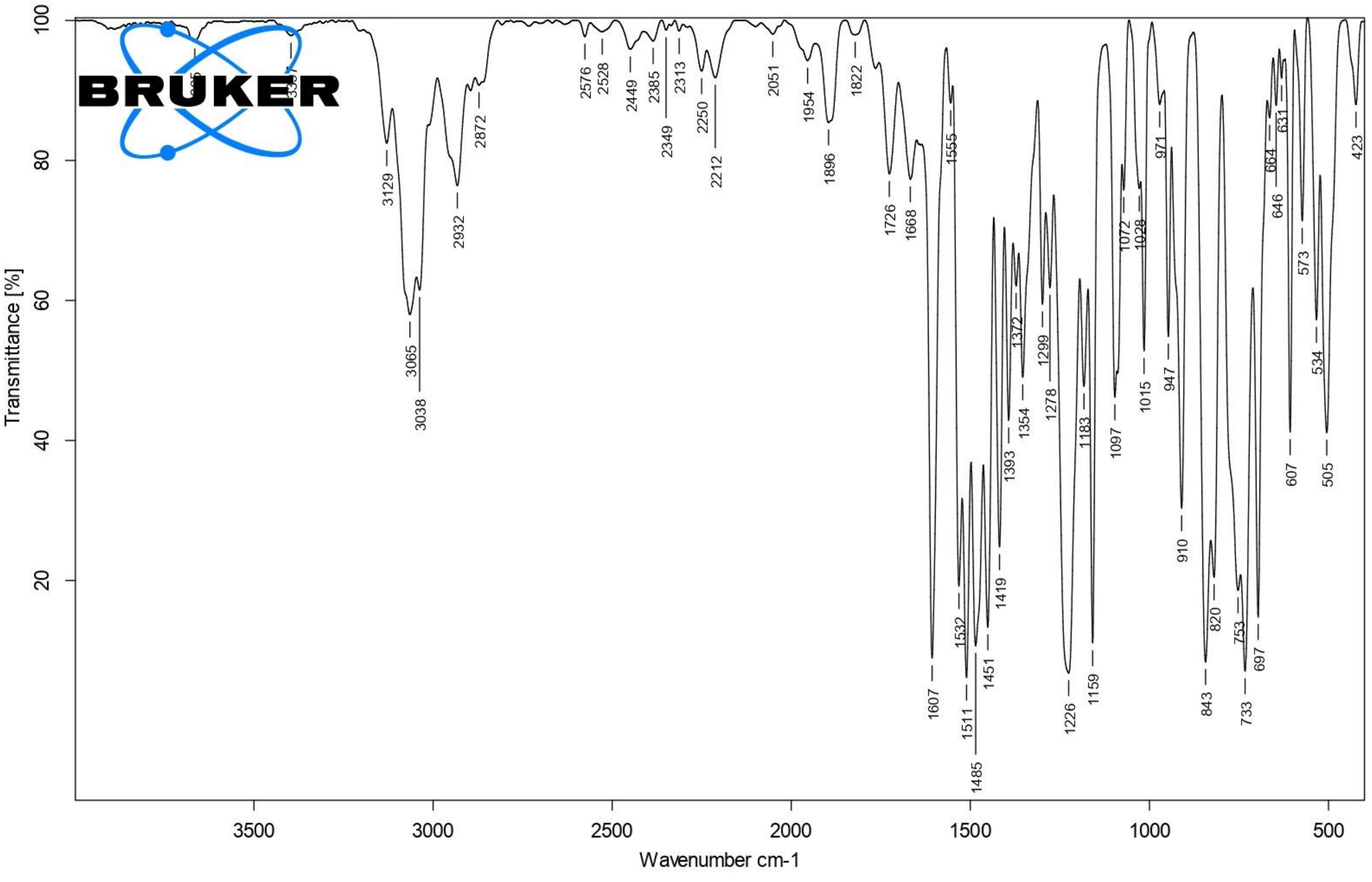

IR of 3p

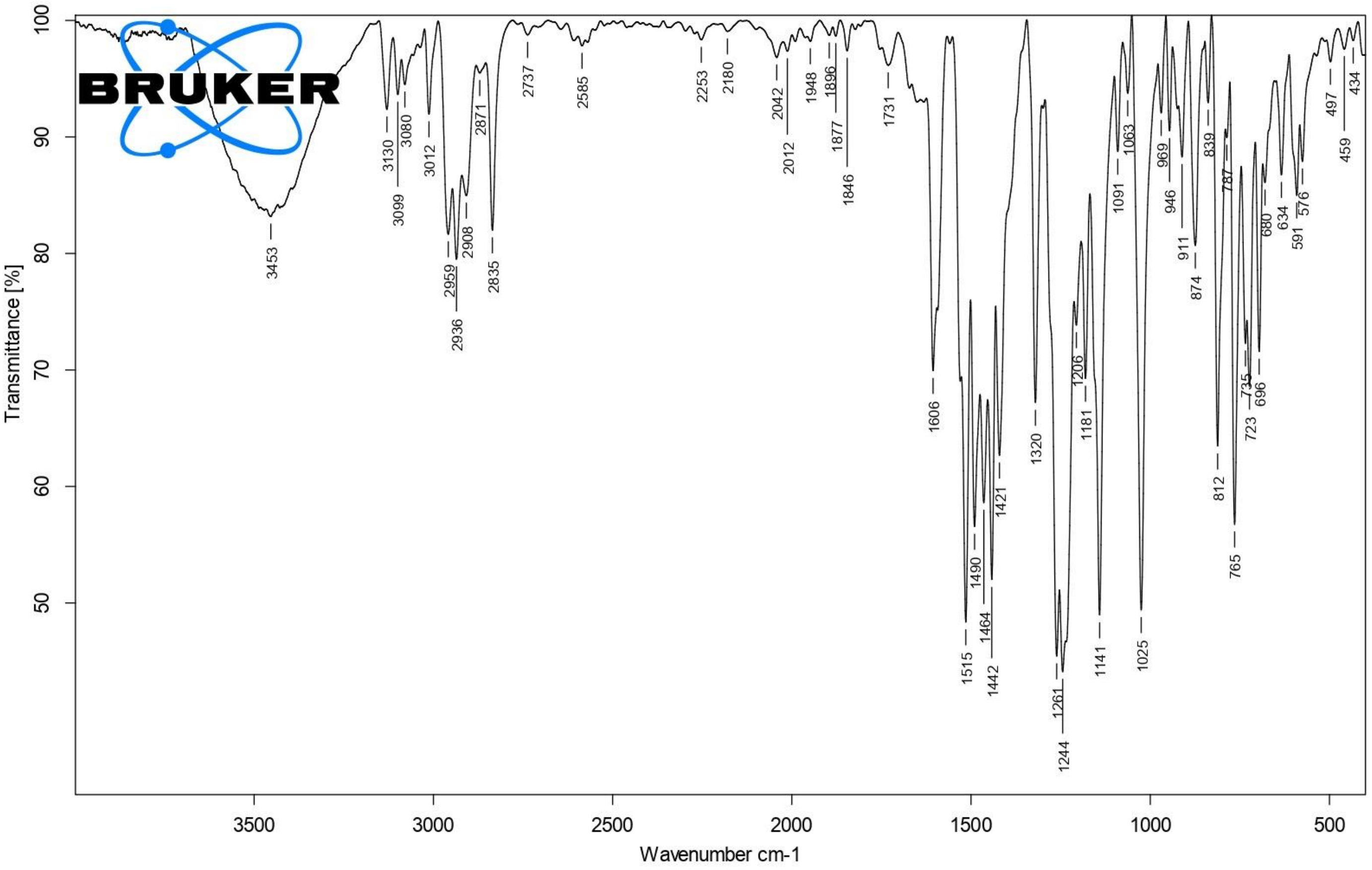

IR of 3q

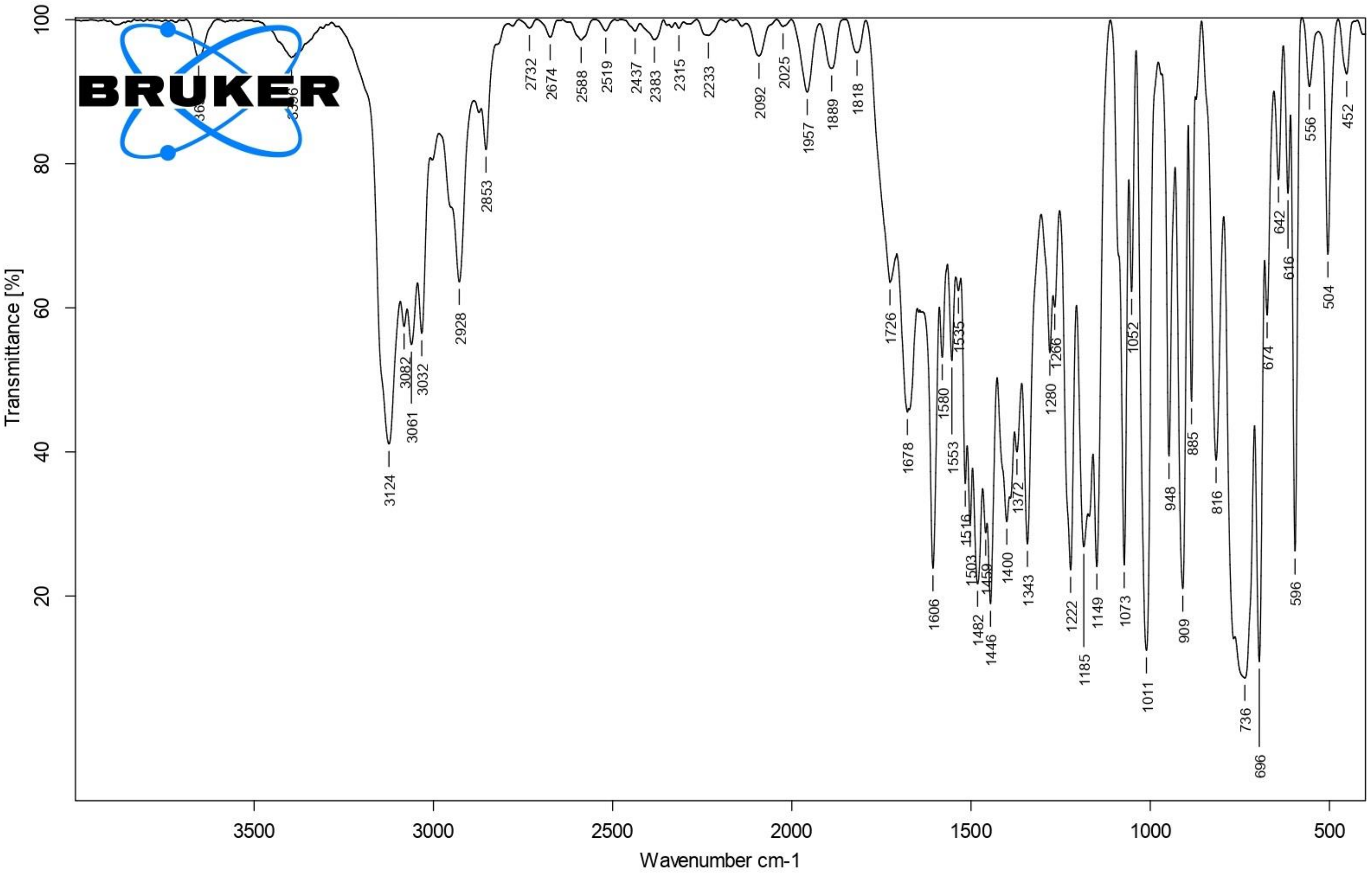

IR of 3r

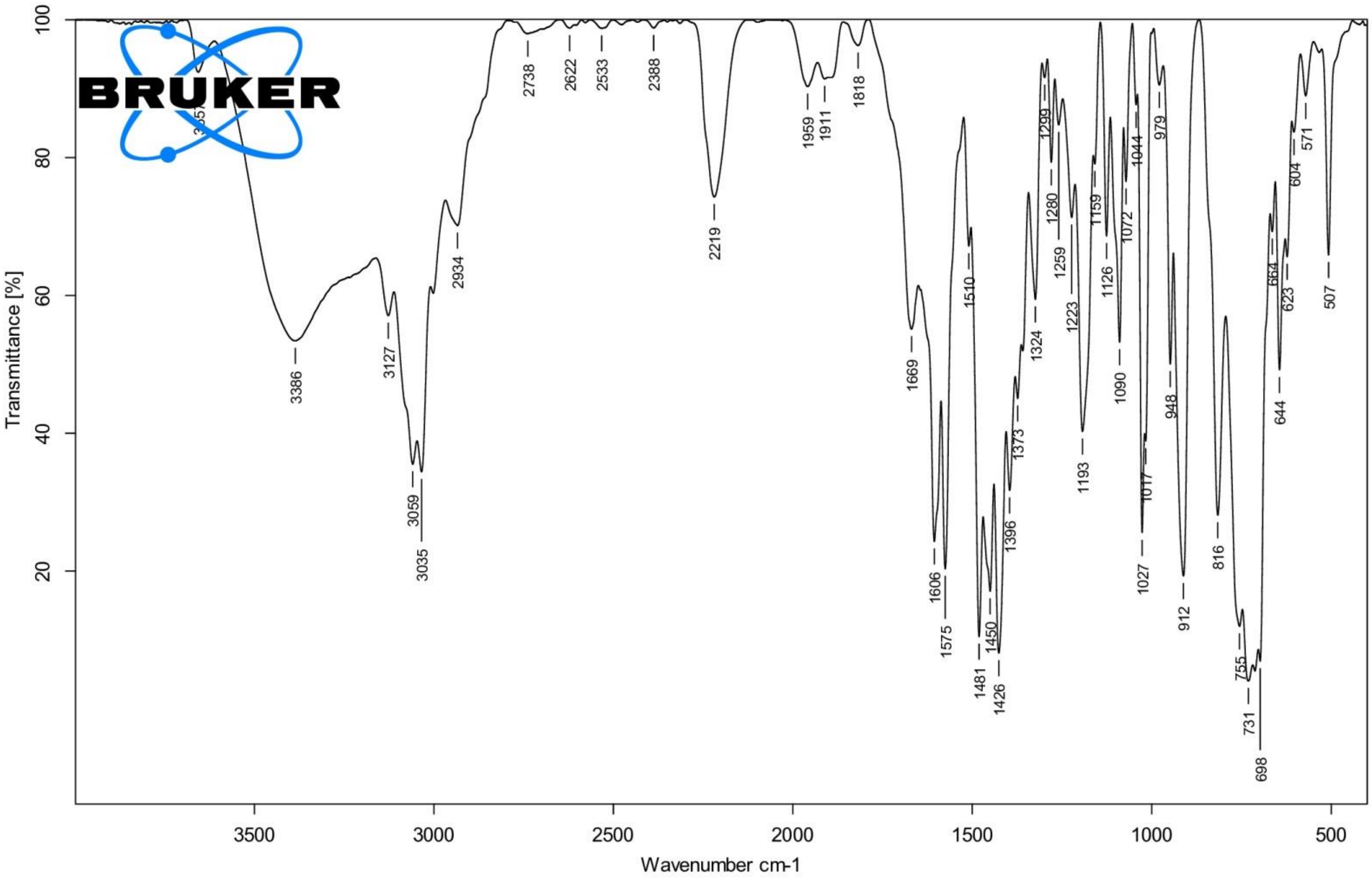

Supplement: Supplementary file 1 [file molecules-27-07721-s001.zip › molecules-2019375-supplementary.pdf]
